# Half‐Pipe Melt Electrowritten Scaffolds Support Engineering of an Immunocompetent Hydrogel‐Embedded Intestine‐on‐a‐Chip

**DOI:** 10.1002/advs.202507132

**Published:** 2025-07-10

**Authors:** Robine Janssen, Henrike S. Schulze, Claire M. L. Nelissen, Marta G. Valverde, Andrei Hrynevich, Govardus A. H. de Jong, Anne Metje van Genderen, Jos Malda, Shanna Bastiaan‐Net, Linette E. M. Willemsen, Rosalinde Masereeuw

**Affiliations:** ^1^ Department of Pharmaceutical Sciences Pharmacology Utrecht University Utrecht 3584 CG The Netherlands; ^2^ Department of Orthopaedics University Medical Center Utrecht Heidelberglaan 100 Utrecht 3584 CX The Netherlands; ^3^ Department of Clinical Sciences Faculty of Veterinary Science Utrecht University Yalelaan 108 Utrecht 3584 CM The Netherlands; ^4^ Wageningen Food & Biobased Research Wageningen University & Research Wageningen 6708 WG The Netherlands

**Keywords:** 3D printing, biofabrication, food allergies, immunocompetent intestine‐on‐a‐chip, in vitro models

## Abstract

In vitro models that mimic intestinal mucosal tissue inflammation and assess the sensitizing capacity of food proteins are essential for understanding food allergy mechanisms and improving safety assessments. Current 2D models lack spatial epithelial‐immune cell interactions, including dendritic cell (DC) migration and DC–T cell crosstalk. Intestine‐on‐a‐chip (IoC) models are used for infections and inflammatory bowel disease (IBD) but are not yet widely used in food allergy research. Here, a 3D immunocompetent IoC model is presented using extrusion‐based bioprinting and melt electrowriting (MEW). The system integrates human intestinal epithelial cells (Caco‐2) seeded on half‐pipe‐shaped MEW scaffolds and co‐cultured with hydrogel‐embedded monocyte‐derived DCs (moDCs) inside the printed device. Subsequent moDC–T cell interactions are studied separately in a hydrogel‐embedded system. IoCs exhibited leak‐tight epithelial barriers comparable to transwell‐like systems, while demonstrating higher metabolic and brush border enzyme activity, and lower LDH leakage. After food allergens (peanut, milk, and egg), non‐allergen (Rubisco) or pro‐inflammatory stimuli (toxin A and LPS) exposure, distinguishable effects on epithelial barrier integrity and moDC driven Th1/Th2 immune responses are observed. The IoC model presents a significant step toward 3D in vitro systems that mimic the intestinal mucosa's compartments to study food allergen sensitization and inflammatory diseases.

## Graphical abstract

The immunocompetent Intestine‐on‐a‐Chip (IoC) is developed using melt electrowritten (MEW) half‐pipes. Intestinal epithelial cell (IEC) culture is optimized in the MEW‐based IoC, followed by co‐culture with monocyte‐derived dendritic cells (moDCs) and T cells in 3D hydrogels. This model supports IECs with a porous membrane, and provides a base for mimicking the step‐wise immune response as well as facilitating cell–cell interactions.

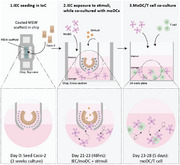

## 1 Introduction

Intestine‐on‐a‐chip (IoC) technologies have advanced significantly, yet challenges remain in replicating the organ's natural curvature, 3D structures, and physiological relevant incorporation of immune cells, including their migration within extracellular matrix (ECM)‐like hydrogels.^[^ ^1^ ^]^ Traditional as well as advanced in vitro models often lack these complex features, limiting their ability to fully emulate (mucosal) intestinal physiology or fall short in the inclusion of a dynamic immune cell component. Recently developed biofabrication methods, such as melt electrowriting (MEW), offer promising solutions to these limitations by enabling precise construction of 3D architectures, thereby facilitating the development of more physiological relevant in vitro tissue models. In MEW, a molten medical‐grade polymer, like polycaprolactone (PCL), is extruded through a nozzle using air pressure while a high voltage is applied between the nozzle and the collector. The produced fiber can be deposited with exceptional accuracy, both in precisely controlled fiber shape and alignment.^[^ ^2^ ^]^ This allows forming intricate 2D layers that can be systematically stacked to create complex 3D structures, such as tubular geometries.^[^ ^3^ ^]^ These scaffolds are typically explored for their capacity to guide cell growth and behavior^[^ ^3^, ^4^ ^]^ and to enhance hydrogel reinforcement^[^ ^5^ ^]^ in both tissue engineering and in vitro modeling. Fused filament fabrication (FFF), a method involving the extrusion of a biocompatible thermoplastic polymer like polylactic acid (PLA) through a nozzle onto a plate to form a 3D object by stacking layers of materials,^[^ ^6^ ^]^ is well suited for creating microfluidic platforms (organ‐on‐a‐chip (OoC)). The convergence of sophisticated biofabrication techniques with IoC systems holds potential for creating more accurate and functional in vitro models,^[^ ^7^, ^8^ ^]^ including an immunocompetent intestine.

Customizable MEW scaffolds to create IoC models with physiological mimicry are particularly relevant for studying intestinal diseases like infection,^[^ ^9^ ^]^ inflammatory bowel disease (IBD),^[^ ^10^ ^]^ and food allergies,^[^ ^11^ ^]^ where the interaction between epithelial and immune cells plays a critical role. Toxins, such as toxin A from *Clostridioides difficile* (formerly *Clostridium difficile; C difficile*), are well‐established disruptors of the intestinal epithelial barrier as was previously utilized in an IoC model to assess epithelial barrier integrity and cellular responses to bacterial toxins.^[^ ^12^ ^]^ Moreover, type 1 driving stimulants like lipopolysaccharide (LPS) are commonly applied in IoC models to induce a generic Th1‐skewed inflammation via LPS induced monocyte‐derived dendritic cell (moDC) maturation. Type 1 inflammation plays a key role in host defense during infections and a pathologic role in IBD.^[^ ^13^, ^14^ ^]^ The combination of LPS stimulation with epithelial barrier disruption would further enhance the simulation of inflammatory conditions in IoCs by promoting the translocation of bacterial components into the intestinal tissue, thereby mimicking pathophysiological processes more accurately.^[^ ^10^ ^]^ Beyond inflammatory diseases, IoC models also offer valuable platforms to study food allergies, which have increasingly been recognized as a growing public health issue, impacting individual patients and leading to significant healthcare costs.^[^ ^15^ ^]^ Common food allergens include proteins present in hen's egg, milk, peanut, soy, nuts, shellfish, wheat, fish, and crustaceans.^[^ ^16^ ^]^ Allergenic proteins often survive intestinal digestion, and beyond environmental triggers (such as pollutants and bacterial toxins), these allergens themselves may have the intrinsic capacity to induce allergic sensitization. Allergens can disrupt the epithelial barrier allowing these intact proteins or large peptides to access the mucosal immune compartment, and/or trigger epithelial activation leading to release of type 2 driving danger signals, cytokines, and chemokines (e.g., TSLP, IL33, CCL22) that activate dendritic cells (DCs). Activated DCs process the allergens, upregulate OX40L, and present antigens via major histocompatibility complex (MHC) II to naive T cells, promoting their differentiation into T helper 2 (Th2) cells and the release of Th2 cytokines (e.g., IL13, IL4 and IL5).^[^ ^17^ ^]^

Current research into food allergen sensitization processes relies on animal models, which raise ethical concerns according to the 3R principles and are limitedly translational to humans due to species‐specific differences. In vitro models combining epithelial cells and immune cells to assess the sensitizing capacity of food allergens have been developed,^[^ ^18^, ^19^, ^20^, ^21^ ^]^ predominantly based on 2D Transwell or transwell‐like systems (TW) assays, while more advanced IoC models are frequently applied to study infections, inflammation, or IBD features, but not yet to investigate food allergy. Intestinal epithelial cells (IECs) can be co‐cultured with immune cells in vitro, however, integrating functional immune components like cell migration and cell–cell interactions remains challenging. IECs sequentially co‐cultured with DCs, T cells, and B cells could simulate food allergen sensitization after hen's egg protein ovalbumin (OVA) exposure.^[^ ^19^, ^20^ ^]^ Despite their robustness and reproducibility, direct immune cell migration and IEC interaction could not be modeled, since IEC and immune cells are cultured in cell culture medium in separate compartments. Therefore, advanced 3D in vitro models are needed to better replicate the complex tissue dynamics of food allergies. For this, bio‐fabricated and ‐printed models hold promise of providing new in‐depth mechanistic insights into both allergic sensitization and tolerance processes, or other immunologic mechanistic processes, thereby advancing our capacity to create novel therapeutics. Furthermore, such models could serve as valuable tools to assess the sensitizing allergenicity of both existing and newly developed food proteins, thus contributing to a safe food supply for a growing global population.

With the aim to replicate the first steps in food allergic sensitization, alongside a general IEC inflammatory response, half‐pipe MEW scaffolds were printed and inserted into a device to develop a MEW‐based immunocompetent IoC. To validate our IoC model, we compared the IoC system to the conventional TW model. We used Caco‐2 cells in our model, a widely adopted human cell line in intestine‐on‐chip (IoC) systems due to their reliable differentiation into a small intestinal phenotype. Their ability to form a strong, functional epithelial barrier makes these cells particularly suitable for studies involving barrier disruption. Next, we performed a preliminary study in which we combined moDCs embedded in 3D hydrogels with our IoC, followed by peanut, milk or egg allergen or toxin A combined with LPS exposure and subsequent 3D co‐culture of collected moDCs with T cells. Our 3D immune cell‐integrated IoC model presents a promising platform to more closely recapitulate the complexity of mucosal tissue architecture, while enabling the incorporation of dynamic immune cell interactions.

## 2 Results

### 2.1 IoC Materials are Biocompatible for Use in Immunomodulatory Studies

**Figure 1:**
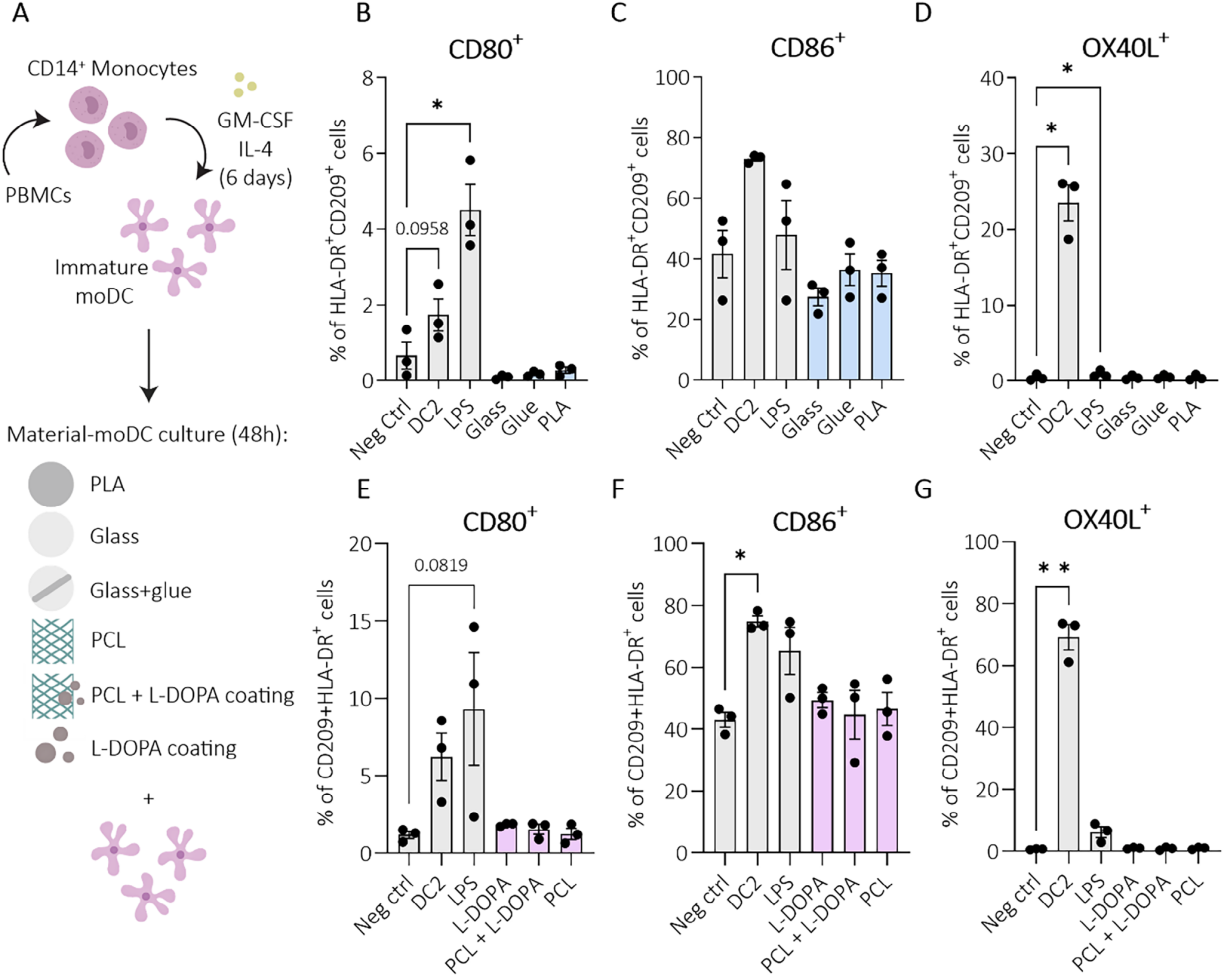
Effect of IoC materials on moDCs. A) Schematic overview of the experimental set‐up; monocytes were isolated from B–D) frozen (three independent donors, N = 3) or E–G) fresh peripheral blood mononuclear cell (PBMCs) (three independent donors, N = 3), followed by differentiation into moDCs and exposure to the IoC materials for 48 h. MoDC activation was analyzed by flow cytometry. B) CD80^+^, C) CD86^+,^ and D) OX40L^+^ were measured upon glass, glue, and PLA exposure. E) CD80^+^, F) CD86^+,^ and G) OX40L^+^ were measured upon L‐DOPA, PCL, and PCL+L‐DOPA exposure. Glue = Loctite adhesive on glass coverslip, DC2 = media supplemented with a type 2 polarizing DC2 cytokine mix (TNFα + IL1β + IL6 + prostaglandin E2), LPS = media supplemented with type 1 polarizing lipopolysaccharide. Data was statistically tested using repeated measures one‐way ANOVA with Geisser‐Greenhouse correction with Dunnett's post hoc, or Friedman's test with Dunn's post hoc. All conditions were tested against the negative control only. Error bars represent mean ± standard error of the mean (SEM). ^*^ = *p* ≤ 0.05; ^**^ = *p* ≤ 0.01. Immune cell illustrations were reproduced and adapted^[^ ^1^, ^8^ ^]^ with permission; permission conveyed through Copyright Clearance Center, Inc.

Devices of PLA‐based OoC technology and PCL‐based MEW structures both stand out for their excellent biocompatibility. To further validate PLA and PCL as suitable materials for immune studies, without directly activating moDCs, the immunogenicity of the various IoC materials was studied. The inertia of PLA (the IoC device), glass (the base), glue (the bonding agent between the IoC device and glass), PCL (MEW half‐pipe) and L‐3,4‐ dihydroxyphenylalanine (L‐DOPA^[^ ^12^, ^22^ ^]^; coating of the MEW half‐pipe) were proven by exposing these materials to moDC for 48 h (**Figure** **1**A). MoDC activation marker expressions were analyzed by flow cytometry (Figure 1B–G; Figures S1 and S2, Supporting Information). Cell viability, negligible expression of CD14 (monocyte marker), and dual positivity for CD209 and HLA‐DR confirmed successful moDC differentiation (Figures S1 and S2, Supporting Information). None of the materials (PLA, Glass, Glue, PCL, L‐DOPA) caused moDC activation. Although not further pursued in this study, polydimethylsiloxane (PDMS), the material commonly used in other OoC studies, was also evaluated, demonstrating no detectable activation of moDCs (Figure S1D–F, Supporting Information). MoDCs cultured out of monocytes isolated from either fresh or frozen peripheral blood mononuclear cells (PBMCs), showed increased CD80 expression in response to type 1 polarizing LPS exposure, followed by a similar trend for the type 2 polarizing DC2 mixture exposure (Figure **1**B, E). DC2 clearly demonstrated an increased CD86 and OX40L expression (Figure **1**C,D,F,G). None of the materials induced moDC maturation, as either type 1 or type 2 moDC maturation marker expression remained low at the level of medium controls. Together, these findings suggest that moDCs are not activated by the IoC materials, indicating their compatibility and suitability for use in immunocompetent IoC systems.

### 2.2 Characterization of the Intestine Half‐Pipe Scaffolds

**Figure 2:**
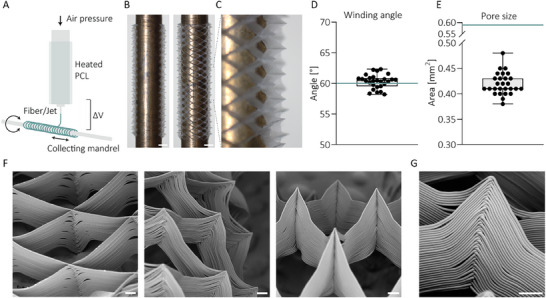
Production and characterization of the MEW half‐pipe scaffold. A) Schematic overview of the MEW process. B) Representative stereomicroscopy images of the bottom (left) and top (right) views of the printed MEW half‐pipe on a 3 mm wide mandrel (scale bar = 1 mm), including C) a close‐up view. The set values of pore size area and winding angle were characterized using stereomicroscopy, in which printed D) winding angle and E) pore size were measured in ImageJ. Blue lines indicate the theoretical set value for pore size and winding angle. Three melt electrowritten (MEW) half‐pipe constructs were analyzed, with each construct evaluated in three distinct regions. Within each region, three measurements of a specific parameter were obtained, resulting in a total of nine measurements per construct. The compiled data are presented as box‐and‐whisker plots, illustrating the distribution of all measurements across the three MEW half‐pipe samples. F) Representative scanning electron microscopy images of MEW fiber stacking (from left to right: 220x, 225x, and 240x magnification), including G) a close‐up view (500x magnification). Scale bars in scanning electron microscopy images = 100 µm.

The first phase in establishing an immunocompetent IoC platform involved the production and validation of intestinal half‐pipe scaffolds. These half‐pipe scaffolds simulate a more 3D structure of the small intestine by providing curvature, thereby offering a more physiologically relevant surface for IEC growth compared to a conventional flat, permeable membrane TW insert setup. Notably, utilizing only 75% of the tubular geometry improves accessibility to the inner luminal intestinal surface, thus simplifying the cell seeding process and transepithelial electrical resistance (TEER) measurements. The half‐pipe scaffolds were fabricated via MEW using optimized printing parameters (Figure S3, Supporting Information), in which microscale PCL fibers were deposited on a moving and rotating mandrel (**Figure** **2**A), resulting in the formation of PCL half‐pipe scaffolds with a rhombus‐pattern (Figure 2B,C). We observed that the average of the winding angle (60.31°) (Figure 2D) closely mirrored the preset values of 60°. The rhombus‐shaped pores span an area of ≈0.42 mm^2^, which is slightly smaller than the set value of 0.589 mm^2^ (Figure 2E). This discrepancy is likely caused by the fact that the curved 3D area of the pores was measured in a flat 2D image. Visual confirmation of the fibers’ stacking and alignment is provided by scanning electron microscopy images (Figure 2F), including a close‐up view of their intersection points (Figure 2G).

### 2.3 Design of the IoC Device

A microfluidic IoC device was fabricated using Fused Filament Fabrication (FFF), which houses the MEW half‐pipe scaffold (Figure S4A,B, Supporting Information). This device enables passive perfusion through the luminal (apical) side of the half‐pipe scaffold, thereby applying shear stress via a 3D rocker system. Moreover, it concurrently allows access to both the apical and basolateral compartments, which is instrumental for TEER measurements. Furthermore, access to the apical compartment (IEC luminal side) is pivotal for cell seeding and controlled introduction of compounds, such as allergens. By allowing easy access to the basolateral compartment, the system facilitates both the seamless integration of immune cells at a later stage and sampling at multiple timepoints. Central to the IoC's architecture is a scaffold holder accommodating the MEW half‐pipe scaffold, including a rectangle‐shaped opening for viewing and cell–cell interaction. The scaffold is encircled and supported by ledges to ensure stable placement. The holder's length is intentionally slightly shorter than the scaffold, inducing a mild compression that keeps the scaffold in place. Two square‐shaped chambers provide a reservoir for media and enable perfusion through channels. The two media chambers, along with the luminal side of the half‐pipe scaffold, constitute the apical compartment, while the space beneath and flanking the scaffold holder creates the basolateral compartment (Figure S4A–C, Supporting Information). In addition, the gravity driven flow ensures fast and efficient compound delivery to the scaffold, reaching apical equilibrium within 10 min (Figure S5, Supporting Information).

### 2.4 Collagen‐Loaded MEW Half‐Pipe Scaffolds Provide an ECM Base Support for IECs Culture

**Figure 3:**
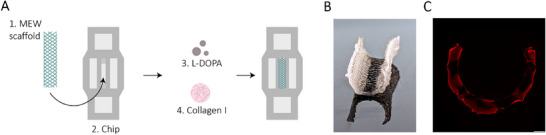
IoC biofunctionalization and collagen casting procedure. A) Schematic overview of MEW half‐pipe L‐DOPA coating and collagen casting workflow. The MEW half‐pipe scaffold was inserted inside the IoC. The MEW scaffold was coated with L‐DOPA and subsequently cast with collagen I hydrogel inside the IoC. B) Macro‐image of a collagen cast MEW half‐pipe scaffold front view (60 µL collagen) (half‐pipe ⌀ = 3 mm). C) Representative confocal image (compiled image of images taken at 4x magnification) of a cross section of a collagen loaded MEW half‐pipe scaffold (70 µL collagen I, in red) (scale bar = 500 µm).

Previous pilot experiments indicated that IECs (Caco‐2 cells) are incapable of independently spanning the rather large gaps between the PCL fibers to form a continuous monolayer (data not shown). As collagen I is the most abundant interstitial ECM protein and is often used in immune cell migration studies,^[^ ^23^, ^24^ ^]^ the MEW half‐pipes were cast with collagen I hydrogel. The IoC was first prepared by biofunctionalization using L‐DOPA. Subsequently, a thin layer of collagen I hydrogel was deposited onto the half‐pipe‐scaffold (**Figure** **3**A). A combination of surface tension and capillary forces facilitates the uniform dispersion of the collagen solution across the fibers, and various hydrogel volumes were tested (Figure S6, Supporting Information). A volume of 60—70 µL reliably filled the pores within the MEW half‐pipe and, at the same time, sealed the half‐pipe within the PLA holder (Figure 3B,C; Figure S6B, Supporting Information).

### 2.5 IECs Successfully form Monolayers on the MEW Half‐Pipe Scaffolds

**Figure 4:**
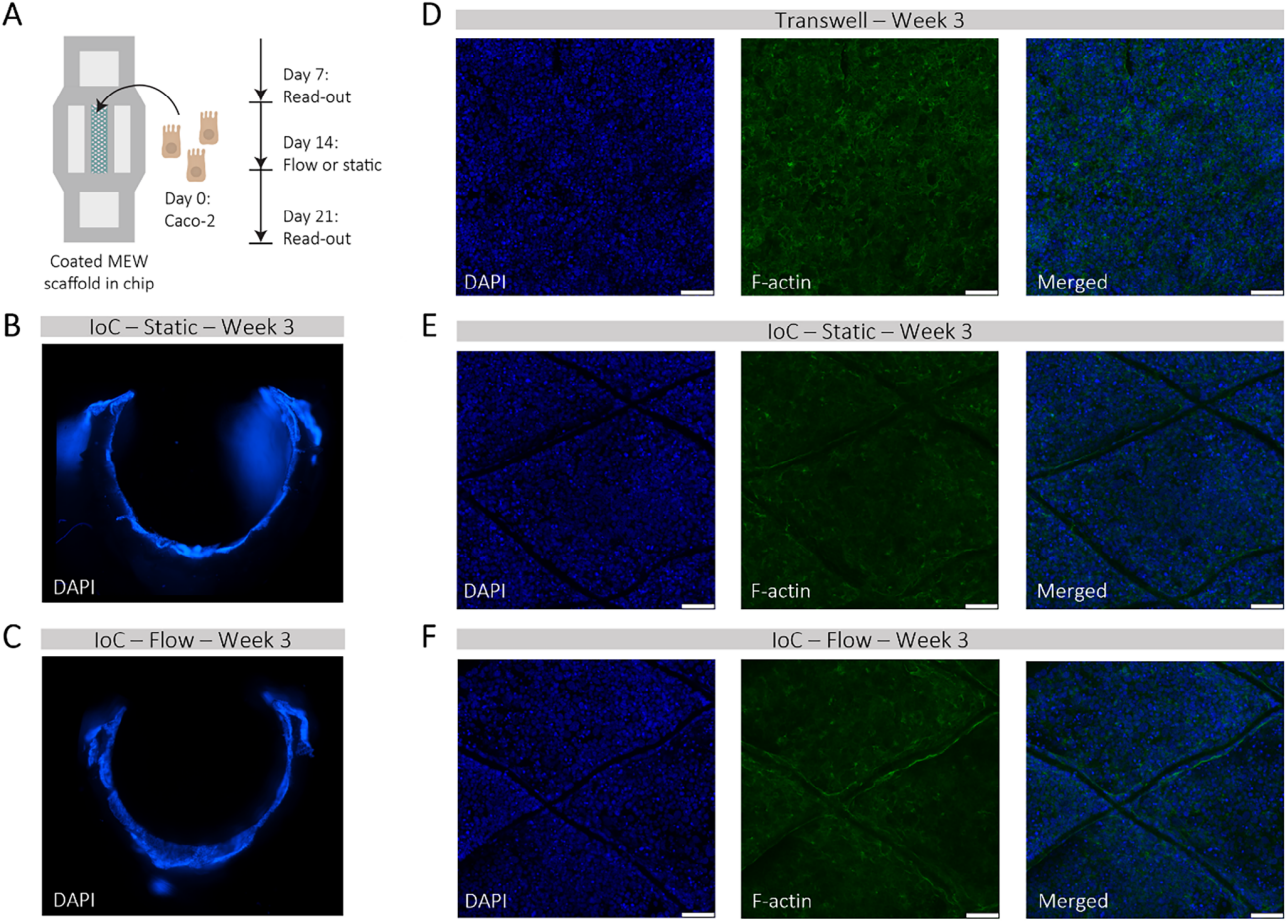
Full cell coverage of IECs on the collagen cast MEW half‐pipe scaffold in the IoC (flow and static conditions) in comparison to a standard TW model. A) Schematic presentation of the IEC seeding process and timeline. IECs form a monolayer within one week, followed by a two‐week maturation phase. Readouts were taken at week one and three, with flow applied or IoCs maintained in a static state from day 14 onward. Representative examples (of the biological replicates, N = 3) of the compiled fluorescent microscope images (of images taken at 4x magnification) show cross‐sections of the IEC half‐pipe (⌀ = 3 mm) after three weeks of culture under B) static or C) dynamic (flow‐applied) conditions. Cell nuclei were stained by DAPI. Representative examples (of the N = 3) of z‐stacks of confocal images (at 10x magnification) showing the top view of IECs onto D) TW filters, E) IoCs in a static environment, and F) IoCs with applied flow. All were cultured for three weeks. Cell nuclei were stained with DAPI (blue), and the cytoskeleton (F‐actin, green) was stained with phalloidin. Scale bar = 100 µm. Caco‐2 cell illustrations were reproduced and adapted^[^ ^1^ ^]^ with permission; permission conveyed through Copyright Clearance Center, Inc.

Following the successful integration of a collagen layer within the IoC system, the Caco‐2 cells were seeded onto the collagen loaded MEW scaffold (**Figure** **4**A). The ideal seeding concentration was determined in prior experiments (Figures S7 and S8, Supporting Information). Furthermore, a 60° angle tilting methodology was developed to ensure a fully covered scaffold (Figure S4D, Supporting Information). Complete monolayer coverage of a TW model is consistently attained within a week. Hence, synchronizing both modules (TW and IoC) allows comparisons and ensures that the systems share a comparable two‐week maturation timeframe to guarantee optimal differentiation toward a simulated small intestinal phenotype. In our IoC, Caco‐2 cells formed a continuous layer on the half‐pipe scaffold after one week, retaining this complete coverage throughout three weeks of culture, irrespective of the presence or absence of fluid flow (Figure 4B,C; Figure S9, Supporting Information). Further insights into cell morphology and coverage were obtained through imaging of the cell‐covered MEW scaffold center and the TW. The images revealed a seamless, gap‐free monolayer characterized by dense cell packing. Morphological differences were not observed between TW and IoC systems after either one or three weeks of culture, under both static and flow conditions (Figure 4D–F; Figure S10, Supporting Information).

### 2.6 IECs Show Increased Metabolic Activity and Reduced Cell Death when Cultured in IoCs Compared to TWs

**Figure 5:**
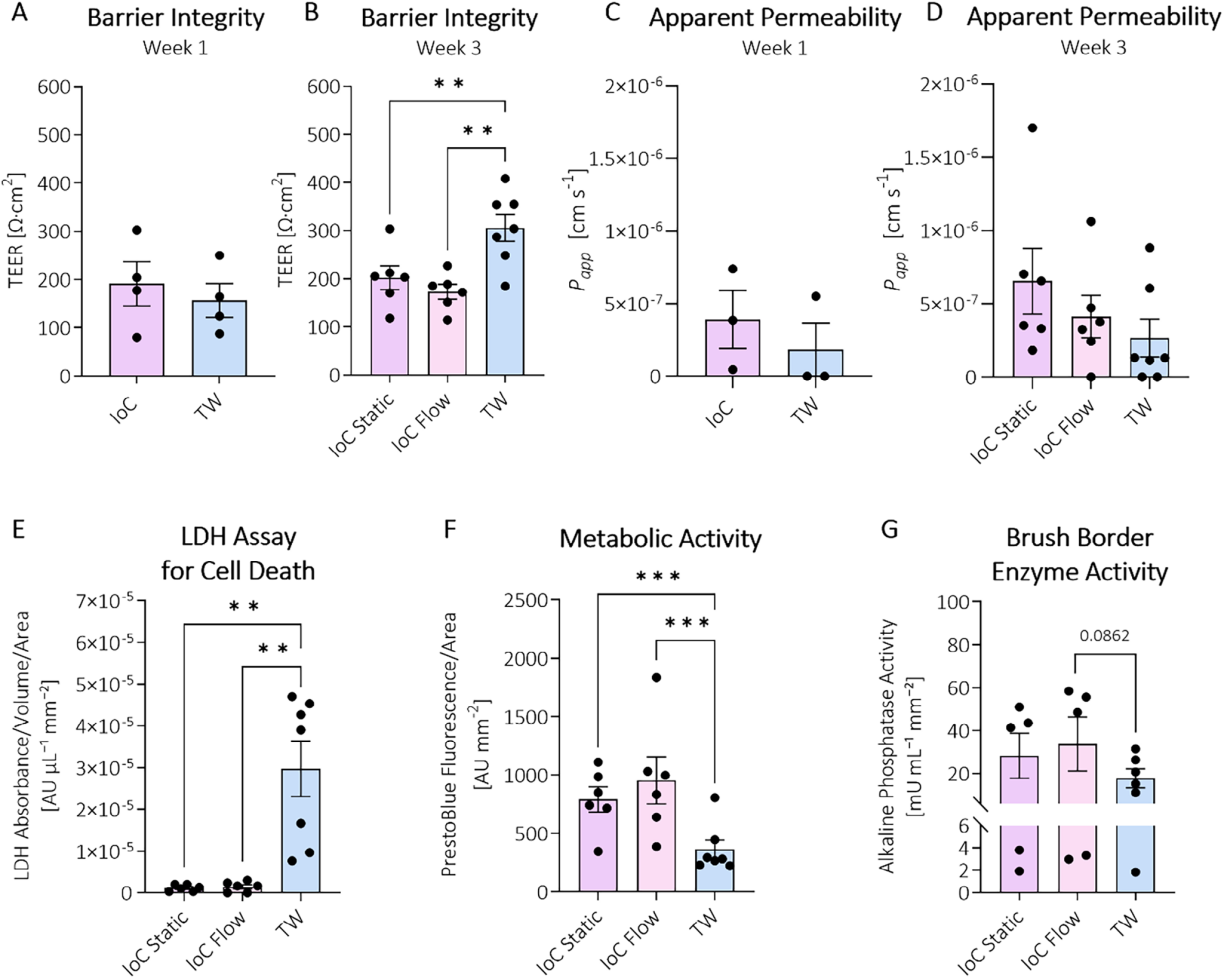
Functionality of IECs cultured on the collagen cast MEW half‐pipe scaffolds in the IoC (flow and static conditions) for one and three weeks in comparison to a standard TW model. Barrier integrity measured by transepithelial electrical resistance (TEER) after A) one week (N = 4) and after B) three weeks (IoC N = 6, TW N = 7) of IEC culture in IoC or TW systems corrected for empty (no cells) controls and cell growth area (Ω·cm^2^). Apparent permeability (*P_app_*) was determined after C) one week (N = 3) and after D) three weeks (IoC N = 6, TW N = 7), both over a 240 min time period of 4 kDa FITC‐dextran exposure. Cell functionality was assessed after three weeks by E) LDH absorbance to measure natural cell death (IoC N = 6, TW N = 7) corrected for apical volume (µL) and cell growth area (mm^2^) of the two different systems, F) PrestoBlue to assess metabolic activity (IoC N = 6, TW N = 7) corrected for cell growth area (mm^2^) and G) alkaline phosphatase enzyme activity assay to measure brush border enzyme activity (IoC N = 5, TW N = 6) corrected for cell growth area (mm^2^) of IECs cultured in either IoCs and TWs. IoC = intestine‐on‐a‐chip, TW = transwell‐like system (ThinCert). Paired *t*‐test, Wilcoxon matched‐pairs signed‐rank test or mixed‐effects ANOVA with Tukey's post hoc was performed. Error bars represent mean ± SEM. ^**^ = *p* ≤ 0.01; ^***^ = *p* ≤ 0.001.

To achieve a more comprehensive comparison between the IoC and TW platforms, barrier and functionality tests were conducted. The barrier integrity was monitored through TEER measurements, after one and three weeks of culture on collagen hydrogel. The barrier integrity of IoCs remained comparable to TWs after one week (Figure 5A; Figure S11A,B, Supporting Information). In contrast, after three weeks of culture, TWs (≈300 Ω·cm^2^) (TEER filter subtracted) developed higher TEER values compared to both IoC static and flow conditions (≈200 Ω·cm^2^) (Figure 5B; Figure S11C,D, Supporting Information). No differences were observed for IoC between static and flow conditions. Additional experiments confirmed lower TEER values (≈50%) once IECs were cultured on collagen hydrogels compared to directly on the TW filter (Figure S12, Supporting Information). Barrier integrity was further assessed via the apparent permeability by using the 4 kDa FITC‐dextran leakage assay. No differences in leakage between TW and IoC were observed after one week (Figure 5C). Similarly, after three weeks of IoC static or flow and TW culture, no differences between the two models were detected following 240 min of 4 kDa FITC‐dextran exposure (Figure 5D). In addition, apparent permeability of both IoC and TW remained low compared to their corresponding controls after one week (Figure S11E,F, Supporting Information) and three weeks (Figure S11G,H, Supporting Information) of IEC culture. Next, cell functionality was assessed, revealing less LDH leakage, which could reflect cell shedding or viability, in IoCs cultured for three weeks compared to TW (Figure 5E; Figure S13A–D, Supporting Information). The metabolic activity of IECs cultured for three weeks in the IoC system was more than two‐fold higher under both static and flow conditions compared to the TW system (Figure 5F; Figure S13E,F, Supporting Information), with no differences observed between the IoC static and flow conditions. In addition, a trend toward higher brush border enzyme activity was observed for IoCs cultured statically compared to TWs, and a similar pattern was observed for IoC under flow conditions (Figure 5G; Figure S13G, Supporting Information).

### 2.7 Epithelial Exposure to Food Allergens does not Affect Barrier Integrity Nor Cell Functionality in the IEC/moDC IoC nor TW

**Figure 6:**
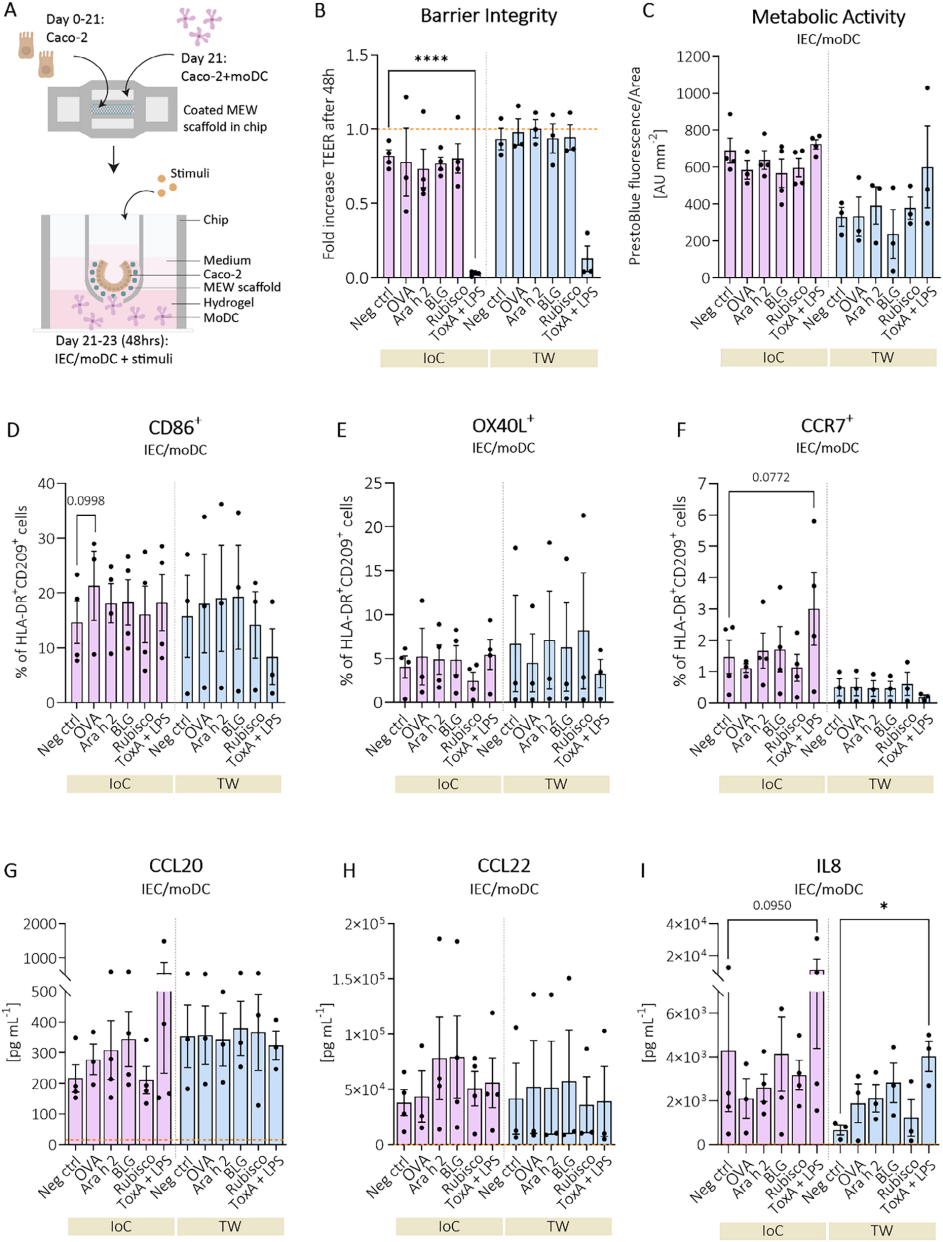
IEC/moDC co‐culture in IoCs is more responsive to toxin A combined with LPS than to food allergens. A) Schematic overview of the inclusion of moDCs and subsequently co‐cultured with T cells. After a three‐week culture of IECs in IoCs and TWs, the systems were co‐cultured with moDCs and subsequently exposed to allergens and hypo‐allergens for an additional 48 h. After these 48 h, the IEC barrier and moDC functionality were assessed. B) A fold increase in barrier integrity after 48 h of allergen exposure in IoC and TW was determined by TEER corrected for empty (no cell) controls and cell growth area (Ω·cm^2^). C) Metabolic activity determined by PrestoBlue assay corrected for cell growth area (mm^2^). Costimulatory moDC markers D) CD86, type 2‐associated moDC marker E) OX40L, and moDC migration marker F) CCR7 measured by flow cytometry. IEC/moDC chemokine G) CCL20, H) CCL22, and I) IL8 secretion, measured by ELISA. IoC = intestine‐on‐a‐chip, TW = transwell‐like system (ThinCert), IEC = intestinal epithelial cell, moDC = monocyte‐derived dendritic cell, Neg ctrl = negative control, ToxA = toxin A, LPS = lipopolysaccharide. Allergens: OVA = ovalbumin (allergen from egg), Ara h 2 (allergen from peanut), and BLG = β‐lactoglobulin (allergen from milk). Hypo‐allergen Rubisco. Gray dotted lines represent datasets that were statistically analyzed separately. The orange dotted line in B at fold increase 1, serve as a reference for comparison of values above or below the baseline. Orange dotted lines in G–I represent the detection lower limits of ELISA assay kits. Data represents three or four individual immune cell donors, each co‐cultured with different biological replicates of Caco‐2 cells. Data was statistically tested using repeated measures one‐way ANOVA with Dunnett's post hoc, mixed‐effects ANOVA with Dunnett's post hoc, or Friedman's test with Dunn's post hoc. All conditions were tested against the negative control only. Error bars represent mean ± SEM. ^*^ = *p* ≤ 0.05, ^****^ = *p* ≤ 0.0001. Caco‐2 and immune cell illustrations were reproduced and adapted^[^ ^1^, ^8^ ^]^ with permission; permission conveyed through Copyright Clearance Center, Inc.

The next step toward the development of a gut‐immune‐axis model for food allergen sensitization was studying the crosstalk between IECs and moDCs following food allergen exposure (Figure 6A). To achieve this, the food allergens OVA, BLG, and Ara h 2, the hypo‐allergen Rubisco, and toxin A combined with LPS (used to induce barrier disruption and a type 1‐polarizing response) were tested. After 48 h of IEC/moDC co‐culture and exposure to stimuli, moDCs were retrieved from the co‐cultures, and IEC barrier functionality tests were performed for both IoC and TW systems. In most IoC conditions, none of the allergens or the hypo‐allergen affected barrier integrity, while exposure to toxin A and LPS resulted in complete loss of TEER in IoCs with similar patterns for TWs (Figure 6B; Figure S14A,B, Supporting Information). Metabolic activity, cell viability, and brush border enzyme activity of IECs (in co‐culture with moDCs) in either the IoC or TW were not affected by any of the allergens, hypo‐allergen or toxin A combined with LPS (Figure 6C; Figure S14 C–H, Supporting Information).

### 2.8 IEC/moDC Co‐Cultures are Sensitive to Toxin A with LPS, but Minimally Affected by Food Allergens

Since no major differences were observed between static and dynamic (flow) IoCs, we proceeded with the static IoC for further studies. MoDCs, differentiated from freshly isolated CD14^+^ monocytes (Figure S15, Supporting Information), were co‐cultured with IECs in both IoCs and TWs. After 48 h, moDCs were retrieved from the IEC/moDC co‐cultures (Figure 6A) and stained to analyze their phenotype and activation state by flow cytometry (Figure S16A, Supporting Information) as well as through analysis of cytokine/chemokine secretion. MoDC viability, co‐expression of HLA‐DR^+^CD209^+^ and absence of the CD14 marker remained comparable between all conditions, indicating that the DC‐phenotype of the cells was maintained in both IoC and TW models (Figure S17A–C, Supporting Information). Co‐stimulatory marker CD86 (Figure 6D) along with the type 2‐associated marker OX40L (Figure 6E), was unaffected except for OVA, which tended to increase the percentage of CD86‐expressing moDCs in the IoC system (p = 0.0998). None of the allergens or hypo‐allergens induced migration marker CCR7 on moDC, while toxin A with LPS showed a strong tendency (p = 0.0772) toward increased CCR7 expression in the IoC system (Figure 6F). Furthermore, secretion of CCL20 (Figure 6G) and CCL22 (Figure 6H) remained comparable between the different stimuli. Toxin A and LPS exposure induced IL8 secretion in the TW system with a similar pattern in the IoC (p = 0.0950; Figure 6I). This effect was further supported by the shift in the CCL22/IL8 ratio, showing a trend toward higher type 1‐associated IL8 levels compared to type 2‐associated CCL22 levels following stimulation with toxin A combined with LPS (p = 0.0819; Figure S17D, Supporting Information). CD80 expression, IL12p70, and IL10 secretion (Figure S17E–G, Supporting Information) showed no differences between conditions, whereas IL33 and TSLP were mostly under the detection limit (Figure S17H,I, Supporting Information).

### 2.9 OVA Exposure Increases Type 2 Over Regulatory Balance, while Toxin A Combined with LPS Shifts Toward Type 1 in moDC/IEC Co‐Cultures

**Figure 7:**
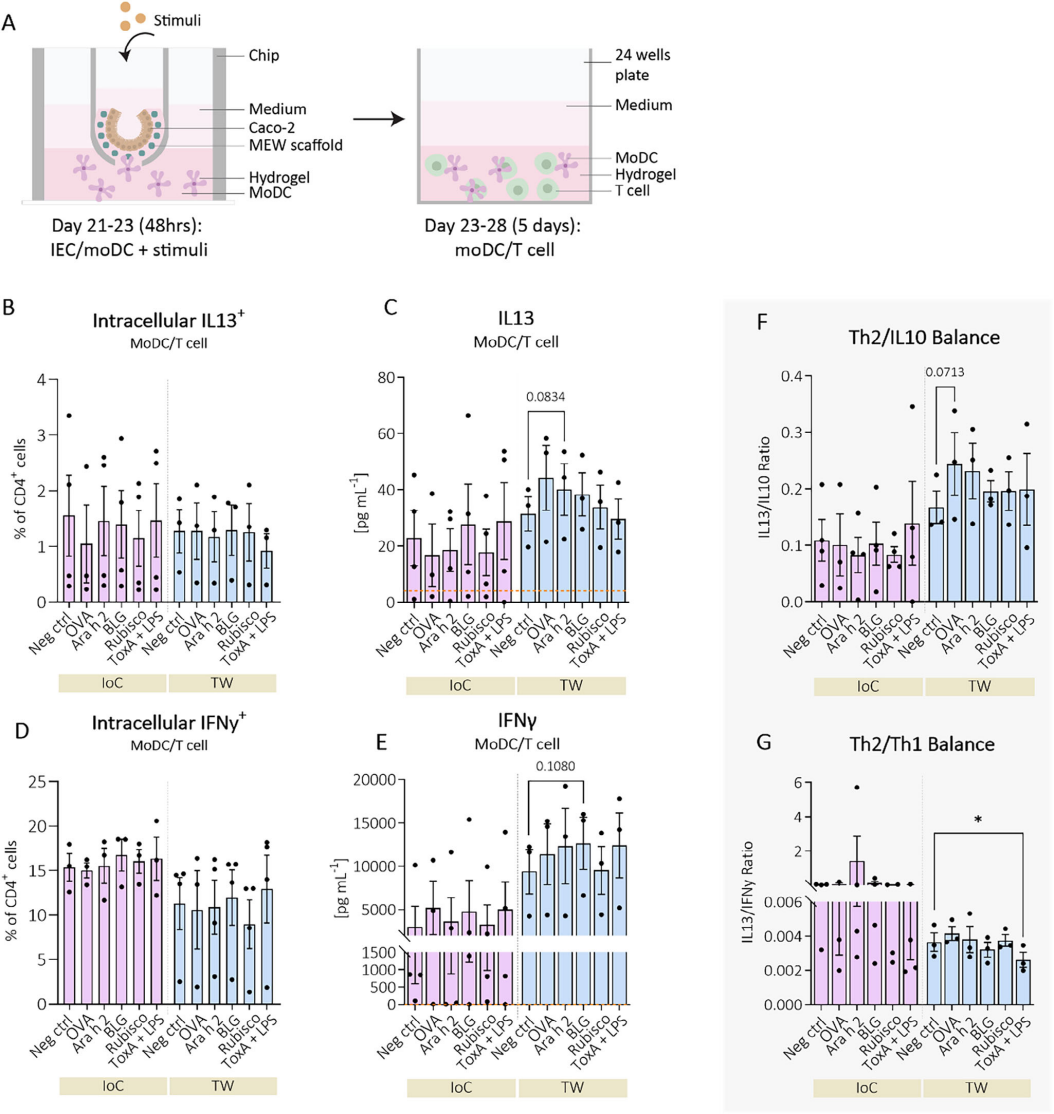
T cells co‐cultured with moDCs derived from TWs respond to both food allergens as well as toxin A combined with LPS. A) Schematic overview of the co‐culture of T cells together with moDCs derived from IoC or TWs IEC/moDC co‐cultures. After a three‐week culture of IECs in IoCs and TWs, the systems were co‐cultured with moDCs in hydrogel, and subsequently IECs were exposed to allergens and hypo‐allergens for an additional 48 h. After these 48 h, moDCs were retrieved from both IoC and TW IEC/moDC co‐cultures and subsequently co‐cultured with T cells in hydrogel for 5 days. After 5 days, T cell functionality was assessed. Intracellular expression of B) IL13 and secreted C) IL13 were measured by flow cytometry and ELISA, respectively. Similarly, intracellular D) IFNγ and secreted E) IFNγ levels were assessed using the same methods. F) Th2/regulatory balance calculated by the IL13/IL10 ratio (IL10 was measured by ELISA, Figure S18H, Supporting Information) and G) Th2 over a Th1 response calculated by IL13/IFNγ ratio. IoC = intestine‐on‐a‐chip, TW = transwell‐like system (ThinCert), IEC = intestinal epithelial cell, moDC = monocyte‐derived dendritic cell, Neg ctrl = negative control, ToxA = toxin A, LPS = lipopolysaccharide. Allergens: OVA = ovalbumin (allergen from egg), Ara h 2 (allergen from peanut), and BLG = β‐lactoglobulin (allergen from milk). Hypo‐allergen Rubisco. Gray dotted lines represent datasets that were statistically analyzed separately. Orange dotted lines represent the detection lower limits of ELISA assay kits. Data represents three or four independent immune cell donors. Data was statistically tested using repeated measures one‐way ANOVA with Dunnett's post hoc, Geisser‐Greenhouse correction with Dunnett's post hoc, mixed‐effects ANOVA with Dunnett's post hoc, or Friedman's test with Dunn's post hoc. All conditions were tested against the negative control only. Error bars represent mean ± SEM. ^*^ = *p* ≤ 0.05. Caco‐2 and immune cell illustrations were reproduced and adapted^[^ ^1^, ^8^ ^]^ with permission; permission conveyed through Copyright Clearance Center, Inc.

After 48 h of IEC/moDC exposure, the moDCs were extracted from the IoC and TW systems and subsequently co‐cultured for 5 days with naive CD4^+^ T cells in a 3D hydrogel (**Figure** **7**A). After 5 days, T cell phenotype was determined by flow cytometry (Figure S16B, Supporting Information), which revealed unaltered viability, CD4 expression, and surface marker expression of the type 2‐associated CD294 (CRTH2) and type 1‐associated CD183 (CXCR3) (Figure S18A–D, Supporting Information). Intracellular IL13 expression in Th2 cells remained comparable across stimuli (Figure 7B). In contrast, exposure to Ara h 2 in the TW system showed a trend toward increased IL13 secretion (p = 0.0834; Figure 7C), with a similar upward pattern observed following OVA and BLG exposures. Intracellular type 1 IFNγ was not affected (Figure 7D), whereas secreted IFNγ tended to increase in the TW system upon BLG exposure (p = 0.1080; Figure 7E). The ratio of secreted Th2 derived IL13 to regulatory IL10 showed a trend toward a Th2‐skewed response over a regulatory response upon OVA stimulation in the TW system (p = 0.0713; Figure 7F). Additionally, the ratio of secreted IL13 to IFNγ indicated a shift toward a Th1‐dominant response following stimulation with toxin A combined with LPS in the TW system (Figure 7G). Intracellular IL10 and IL4, as well as secreted IL10, IL4, and IL5 (Figure S18E–I, Supporting Information), remained unaffected.

## 3 Discussion

In the present study, we introduce a novel MEW‐based IoC featuring a half‐pipe MEW scaffold, fully covered with functional IECs. Building on this innovative model, its complexity was advanced by incorporating moDCs within 3D hydrogels, followed by moDC‐T cell interactions in subsequent 3D hydrogel cultures. We explored barrier disruption and intestinal inflammation using toxin A combined with LPS to drive immune cells toward a type 1 immune response. In addition, we applied the first critical steps for food allergen sensitization in the model, using allergens and a hypo‐allergen to provoke IEC/moDC activation and, consequently, studied if these moDCs gained immunogenic properties and could drive type 2 T cell activation.

To ensure successful immune cell integration, it was crucial to assess whether the IoC's materials and L‐DOPA coating activated moDCs. While previous studies indicate that electrospun PCL scaffolds do not provoke DC responses,^[^ ^25^ ^]^ the impact of material hydrophilicity or hydrophobicity remains controversial. Some research suggested that hydrophobic surfaces enhance DC maturation,^[^ ^26^ ^]^ whereas others reported that hydrophilic PCL promotes DC adhesion and proliferation.^[^ ^27^ ^]^ Given these conflicting findings, we directly evaluated moDC interactions with all IoC materials, including PDMS (not further used in our IoC), PCL, and the L‐DOPA coating. Our results confirmed that none of the IoC materials, MEW structures or biofunctionalization (L‐DOPA) altered moDC phenotype or activation, effectively ruling out material‐induced effects.

After optimizing the design of the MEW half‐pipe scaffolds and IoC devices, we successfully integrated the MEW half‐pipes into the IoC and sealed all pores in the half‐pipes with collagen I hydrogel. Optimization of cell seeding concentration and methodology resulted in a fully grown IEC monolayer on the MEW half‐pipe scaffolds within the IoC, which was maintained over a time span of three weeks. Unlike many organ‐on‐chip (OoC) studies using commercial microfluidic chips,^[^ ^13^, ^28^, ^29^ ^]^ our custom‐built IoC device with bioprinted MEW half‐pipes balances flexibility and complexity. While commercial systems operate at a microphysiological scale, our macroscopic design allows better access to and control over apical and basolateral compartments, with higher cell yield and larger sample volumes for bioanalyses. Furthermore, the design allows extension to multiple compartments to support multi‐organ interactions and enables easy handling, TEER measurements, compound exposure, and immune cell co‐culture.

We acknowledge that the comparison between the chip and the TW system, with regard to studying epithelial function and barrier properties, is not a direct one‐to‐one equivalence. However, we selected a TW‐like system as a reference model due to its widespread use and well‐established performance in intestinal epithelial research. While using another OoC system might also be beneficial, the wide variety of IoC models available presents its own challenges. Each system differs in design and functionality, which could potentially introduce more variability or lead to less meaningful comparisons, depending on the specific functional outcomes being assessed. Therefore, validation against the TW system, following appropriate adjustments such as apical volume and cell surface area, was considered the most appropriate approach in this study.

Natural barrier integrity was confirmed by the IoC system's TEER as well as leak‐tightness, critical for toxin, drugs or allergen exposure studies. TEER did not increase between one‐week and three‐week cultures in the IoC system, possibly due to faster differentiation of the Caco‐2 cells as reported earlier for microfluidic devices.^[^ ^30^, ^31^ ^]^ The trend toward higher brush border enzyme activity in IoCs suggests improved polarization, aiding Caco‐2 cell monolayers in regulating microbiota, inflammation, and permeability.^[^ ^32^ ^]^ Dynamic conditions like flow and shear stress enhance IEC morphology, viability, and villi formation, promoting intestinal model maturation.^[^ ^14^, ^28^, ^33^ ^]^ In our IoC, perfusion did not affect viability, TEER, nor permeability, but a slight increase in brush border enzyme activity was observed. We applied similar shear stress parameters to our newly developed IoC, as previously studied in another gut‐fiber‐oC model.^[^ ^12^ ^]^ That model suggested villi formation at 0.006 dyne cm^−^^2^, however, these parameters may have been suboptimal in our IoC due to differences in device architecture and therefore a limitation of the current study. In future studies, fluid dynamics simulations could optimize shear conditions, by adjusting duration, timing, and intensity.^[^ ^31^, ^33^ ^]^ Despite suboptimal flow conditions, the MEW half‐pipe scaffolds in the IoC demonstrated good IEC functionality, as reflected by lower LDH release and higher metabolic activity compared to TWs. This suggests that the biomimetic, porous structure of the IoC, allowing direct contact with the immune cell containing hydrogel, without the structural constraints of filter‐based TWs, may influence epithelial cell behavior differently, highlighting the importance of system architecture in shaping cellular responses.

In both IoC and TW models, collagen I based hydrogels were used as migration matrices for moDCs and T cells. While collagen I is abundant in connective tissues and common in advanced intestinal models,^[^ ^34^ ^]^ native ECMs are more complex. The intestinal ECM comprises a diverse mixture of basement membrane components, including collagen IV, laminins, and proteoglycans^[^ ^35^, ^36^ ^]^ while the lamina propria ECM contains collagens, fibronectins, laminins, and glycosaminoglycans (GAGs), such as heparan sulfate proteoglycans.^[^ ^35^ ^]^ Despite this, collagen I is widely used in 3D immune cell migration studies^[^ ^23^, ^37^ ^]^ and OoC systems.^[^ ^24^ ^]^ Since our focus was on device development and cellular interactions, we chose a simple yet physiologically relevant collagen‐based hydrogel. Although this was advantageous for our study design, the dissolution step using collagenase could be optimized in future studies. For example, employing thermosensitive hydrogels that undergo gelation at 37 °C and liquefy at 4 °C could reduce handling time and cellular stress.^[^ ^38^ ^]^ Such an approach could help streamlining the transition between the IEC/moDC and moDC/T cell assays while minimizing mechanical disruption during immune cell retrieval; however, its application in immune cell studies remains to be explored. Besides, in future studies, advanced technologies such as extrusion, light‐based, and inkjet (bio)printing^[^ ^39^ ^]^ could enhance ECM complexity by enabling precise control over cellular spatial organization, hydrogel architecture, and stiffness or chemokine gradients, thereby better mimicking the properties of lamina propria and draining lymph nodes.

In our study, the MEW scaffolds were used solely for IECs, however, promising results from other research suggest their potential for immune‐related applications as well. For example, small‐pore, box‐shaped MEW geometries enhanced M2 macrophage differentiation and phenotype,^[^ ^40^ ^]^ rhombus‐shaped structures promoted anti‐inflammatory cytokine production in macrophages,^[^ ^41^ ^]^ and small fiber spaced and small‐pore MEW meshes improved T cell expansion for cell therapies.^[^ ^42^ ^]^ The potential advantages of hydrogel structuring within the immunocompetent IoC system, however, remain to be explored and could be a promising future direction.

OoC systems often integrate immune cells without compartmentalization, typically using PBMC mixtures,^[^ ^28^, ^43^ ^]^ without replicating immunological cascades step by step.^[^ ^1^ ^]^ While our system does not yet fully mimic intestinal inflammation or allergic sensitization, it serves as a foundation by incorporating the immunological cascade.^[^ ^11^ ^]^ The first step is epithelial activation, marked by type 2‐driving alarmins (IL33, TSLP, CCL22) and IL8 production.^[^ ^11^, ^19^, ^20^ ^]^ We previously showed OVA activates IECs and moDCs, driving type 2 T cell responses,^[^ ^19^, ^20^ ^]^ with HT‐29 cells being more responsive than Caco‐2.^[^ ^21^ ^]^ Here, Caco‐2 cells co‐cultured with moDCs in IoCs and TWs did not result in IL33 or TSLP elevations upon allergen exposure. Slight, non‐significant increases in CCL20, a DC‐attracting chemokine,^[^ ^44^ ^]^ were observed for allergens or toxin A combined with LPS, and CCL22 tended to increase for Ara h 2 and BLG. These findings suggest that, under the current conditions, the epithelial‐immune co‐culture model does not initiate a strong type 2‐signature in immune markers, but may exhibit early chemokine signaling indicative of mild immune activation. MoDC co‐stimulatory marker CD86, but not OX40L or CD80, showed a trend toward increased expression upon OVA exposure in IoCs, consistent with its role in allergic sensitization.^[^ ^19^, ^45^ ^]^ Additionally, increased IL8 production in toxin A and LPS exposed IEC/moDC co‐cultures in TWs, with a similar trend in IoCs, aligns with its role in host defense or inflammatory intestinal diseases, such as IBD, where it correlates with active inflammation^[^ ^46^ ^]^ and neutrophil recruitment to further amplify the inflammation cascade.^[^ ^47^ ^]^ Alongside, disrupted intestinal barriers in IoCs upon toxin A combined with LPS exposure, with a similar pattern in TWs, confirmed another characteristic of intestinal inflammation,^[^ ^10^ ^]^ and was also previously observed in the toxin A‐exposed Caco‐2 fiber‐oC model.^[^ ^12^ ^]^ These disrupted barriers allow bacterial components such as LPS to enter the lamina propria. Furthermore, DC migration toward draining lymph nodes requires CCR7 expression,^[^ ^48^ ^]^ which showed a tendency to increase in IoCs after toxin A combined with LPS exposure, further confirming moDC activation in these 3D hydrogel embedded conditions.

Next, to study the function of DCs co‐cultured with IEC under the diverse conditions, the moDC were collected and added to naive CD4 T cells. MoDC/T cell co‐culture in 3D hydrogels, using moDCs derived from TWs, showed a tendency toward increased IL13 secretion, a key Th2 cytokine,^[^ ^49^ ^]^ upon Ara h 2 exposure, with similar inclined patterns for OVA and BLG, consistent with previous findings after OVA exposure in 2D HT29‐OVA in vitro models.^[^ ^19^, ^20^ ^]^ Comparable to prior observations for OVA,^[^ ^20^ ^]^ IFNγ secretion tended to increase upon BLG exposure in TWs. The Th2/regulatory balance, calculated by the IL13/IL10 ratio, indicated a tendency toward Th2 skewing upon OVA exposure in TWs. Moreover, exposure to toxin A combined with LPS in TWs suggested a type 1‐skewed response in T cells, reflected by the reduced IL13/IFNγ ratio. These results indicate that the TW‐based co‐culture system, also when using Caco‐2 and culturing moDC and T cells in hydrogel conditions, allows the study of allergen‐induced type 2 activation, while balancing the T cell response toward type 1 under conditions of toxin A/LPS. However, further optimization is required to allow more robust immune signatures, for example, by enhancing the number of immune cells to optimize the chance of interaction. This also applies to the IoC system, which did show modulation in DC markers, but not in DC function as studied by monitoring T cell responses. Inherent variability between (healthy) immune cell donors could not be avoided, as individual differences in basal cytokine release can be attributable to biological variation. Furthermore, since donors did not fully overlap between IoC and TW, no direct comparison between IoC and TW can be performed. Although the IoC model was fully validated, the incorporation of immune cells remains exploratory. Importantly, this study represents an initial step toward the development of immune‐enhanced OoC systems. Demonstrating the feasibility of culturing immune cells within hydrogels in both IoC and TW formats marks a significant advancement. These findings lay the basis for future studies aimed at developing more complex immune cell co‐cultures, and further refining and expanding the model's applicability to a broader range of (stronger) stimuli and experimental conditions while enhancing the number of independent donors.

One of the main limitations of this study is the low responsiveness of Caco‐2 cells to relatively mild stimuli, such as the food allergens OVA, Ara h 2, and BLG. To enhance sensitivity to mild stimuli like allergens, the incorporating of HT‐29 cells could enhance sensitivity to allergens, but their leakiness favors Caco‐2 cells for barrier integrity studies. A Caco‐2/HT‐29 co‐culture may balance permeability and stimulus response. Modifying the Caco‐2 model is promising, but allergen exposure alone may not be sufficient to disrupt gut homeostasis. Caco‐2 IEC barriers in IoC and TW models are relatively resistant to mild stimuli. Recent studies highlight the exposome, including air pollutants, chemicals, and food additives, which impact epithelial (for both gut and skin) barrier permeability, allergy sensitization, inflammation, and microbiome balance.^[^ ^50^ ^]^ These findings point to possible future adaptations of our Caco‐2 IoC 3D model, such as incorporating various pollutants or toxins as adjunct stimuli to better mimic environmental triggers of inflammation and food allergen sensitization. Furthermore, we focused on establishing and optimizing the in vitro IoC model and its co‐culture with immune cells. As an initial step, we employed well‐characterized, non‐living stimuli (e.g., allergens, enterotoxins) to assess immune responsiveness under controlled conditions. Future studies could explore the inclusion of live microbes to further investigate epithelial–immune crosstalk, potentially enhancing the model's physiological relevance for host–microbe interaction research.

In our newly developed model, active and direct cell–cell communication and interactions were facilitated. Future studies should focus on further validation and compartmentalizing the IoC,^[^ ^1^, ^8^ ^]^ allowing immune cells to start in appropriate subunits. MoDCs and naive CD4+ T cells should be in separate compartments, enabling moDC activation before migrating to the T cell compartment at the inductive site, mimicking the lymph node. Here, DCs instruct naive T cells to differentiate into either regulatory or effector T cells, which then home back to the effector site in the intestinal mucosal tissue via the bloodstream. The next step would be to incorporate naive B cells with T cells to enhance physiological relevance.^[^ ^1^ ^]^ In our assay, we used allogeneic moDC–T cell co‐cultures, which makes it challenging to identify T cells that differentiate into allergen specific effector/memory cells. Therefore, incorporating immune cells in subsequent experiments from allergic donors and performing autologous moDC–T cell assays would be valuable, as seen in a 2D in vitro assay for peanut allergic patients.^[^ ^51^ ^]^ This would enable full HLA matching and allergen‐specific T cell activation. Future studies should explore whole protein content, adjuvants, and diverse allergens. Including gastrointestinal digestion effects with digesta samples could enhance physiological relevance but adds complexity due to potential IEC toxicity.^[^ ^52^ ^]^

## 4 Conclusion

With a rising food allergy incidence and the introduction of novel food proteins, advanced models are needed to assess allergenic potential. Our MEW‐based IoC model provides a valuable starting point for future studies, not only on the sensitizing potential of new food proteins, but also on gut inflammation, diseases such as IBD, and drug testing. By integrating epithelial cells, moDCs, and T cells in a dynamic 3D hydrogel system, the model holds great promise to replicate key events in allergic sensitization, as it responds to external stimuli and shows immune activation. To extend the model beyond the gut, we are developing complementary 3D skin models and a skin‐on‐a‐chip system based on MEW scaffolds, representing another key site of sensitization and exposure site of environmental factors. Ultimately, linking both models will enable the establishment of a gut–immune–skin axis, creating a comprehensive in vitro platform to study mechanisms such as food allergy and to support the safety assessment of novel food proteins and other compounds.

## 5 Experimental Section

For details and antibody lists, the readers are referred to the Supporting Information.

### Half‐Pipe Design and Melt Electrowriting

MEW scaffolds were designed with a length of 12.583, 1.5 mm radius, 60° winding angle, and 0.589 mm^2^ pore size. Half‐pipe scaffolds were designed using a 270° open tubular structure rather than a fully enclosed 360° tube. The MEW process utilized a custom‐built device featuring a rotating brass mandrel (⌀ 3 mm) on an *x*‐*y* axis and a custom print head on a movable *z*‐axis. *X*‐*Y*‐*Z* axes control was facilitated by an advanced motion controller (MC403, Trio Motion Technology Ltd.). Medical grade polycaprolactone (PCL, Purasorb PC 12, Corbion) was electrically preheated in a 3 mL glass syringe and extruded through a 27G size metallic nozzle (length of nozzle ≈6.2 mm) at 90 °C under 1.5–3 bar pressure (pressure regulator VPPE‐3‐1‐1/8‐2‐010‐E1, Festo AG). PCL melt was then extruded and accelerated using 6–6.2 kV by a high‐voltage source (LNC 30000–5 POS, Heinzinger electronic GmbH) across a collecting distance of 4 mm. 30 layers of MEW fibers were printed at an effective speed in range 3–4 mm s^−1^. Post‐fabrication, scaffold removal from the mandrel was facilitated through ethanol immersion and subsequent evaporation.

### MEW Imaging and Structural Characterization

Half‐pipe scaffolds microstructure (winding angle, and pore size) was analyzed by stereomicroscopy (Olympus SZ61) and by scanning electron microscopy (Phenom Pro, Phenom World‐ThermoFischer Scientific, Waltham, MA, USA). Scanning electron microscopy was operated at an acceleration voltage of 10–15 kV. Image J (National Institutes of Health) software was employed for image analysis.

### Fabrication and Assembly of IoC System

The fabricated IoC centers around the holder for the MEW half‐pipe scaffold, which was connected to two media reservoirs, enabling fluid flow. Below and to both sides of the scaffold holder was the basolateral compartment. The apical and basolateral compartments were connected via an opening in the scaffold holder, allowing the direct interaction between intestinal epithelium and immune cells. Fusion 360 software (Autodesk) was used for design and 3D printing of PLA filament based IoCs was performed by using Ultimaker 3/S3 (⌀ 2.85 mm white PLA filament, MakerPoint; 0.4 mm nozzle, 0.1 mm (fine) resolution, 100% infill) or Bambu Lab P1S/X1 Carbon (⌀ 1.75 mm white PLA, PolyLite; 0.4 mm nozzle, 0.20 mm resolution, 100% infill). All the printing parameters were set in Ultimaker Cura (Ultimaker) or OrcaSlicer (Bambu Lab).

### Sterilization and Biofunctionalization of IoC and TW Materials

Post‐printing and before assembly of the IoC, 3D printed materials and assembly materials were sterilized before assembly using UV light exposure for 30 min and post‐assembly using 70% ethanol for 30 min, washed with 1x Dulbecco's Phosphate Buffered Saline without calcium and magnesium (PBS, Sigma, D8537) with 5% penicillin and streptomycin (p/s; Sigma–Aldrich) and left to dry for at least 30 min under UV light exposure. The central IoC compartment and TW inserts (ThinCert 24 well, Greiner Bio‐One, 662641) were coated with sterile L‐3,4‐ dihydroxyphenylalanine (L‐DOPA; 2 mg mL^−1^) (Sigma–Aldrich, D9626‐5G) in tris(hydroxyethyl)aminomethane (10 mm) buffer pH 8.5, resulting in the full submersion of the MEW scaffold and TW filter at 37 °C, 5% CO_2_. After 5 h, IoCs and TWs were washed twice.

### Collagen Casting in IoC System and TWs

Collagen I hydrogels (1.7 mg mL^−1^) were created as described previously.^[^ ^23^ ^]^ In short, Bicarbonate (Gibco, 25080–094), 10X Minimum Essential Medium (GibcoTM, 11430030), and PureCol Type I Collagen Solution (Advanced Biomatrix, 5005) were mixed and further diluted with cell culture medium. Collagen hydrogel (60–70 µL) was added to the interior walls and middle of the IoC device, as well as evenly deposited on top of the TW filter (25 µL). Collagen hydrogels were polymerized at 37 °C for 35 min, 5% CO_2_, after which hydrogels were submerged in culture medium, supplemented with 5% Penicillin‐Streptomycin (p/s; Gibco, 15140163) (5% p/s medium) until cell seeding.

### Caco‐2 Cell Culture

Human intestinal epithelial cells (Caco‐2; colorectal adenocarcinoma origin) from ATCC (HTB‐37) were grown in Dulbecco's Modified Eagle's Medium (DMEM) (Gibco, 42430‐025), supplemented with 1% p/s, 10% Fetal Bovine Serum (FBS) (Biowest, S1810‐1‐500U, S00Q3), 2 mm L‐glutamine (Gibco, 25030081) and 1% non‐essential amino acids solution (Gibco, 11140050), at 37 °C, 5% CO_2_. Media was refreshed every 2–3 days, and cells were passaged at 80–90% confluency. For experiments Caco‐2 cells at passage numbers between 32–45 were used. Caco‐2 cell cultures were tested negative for mycoplasma using MycoAlert PLUS Mycoplasma Detection Kit (Lonza, LT07‐710).

### Cell Seeding and Cell Culture in the IoC System and TW

Caco‐2 cells (150 000) were seeded on top of the scaffold in 5% p/s medium, after which the entire system was tilted 60° on its side (Figure S4D, Supporting Information) at 37 °C for 1 h, 5% CO_2_. Then, another round of Caco‐2 seeding (150 000 cells) was performed, and the system was tilted on its other side at 60° at 37 °C for 1 h, 5% CO_2_. The IoC was left in an upright position overnight. For TWs, Caco‐2 cells (59 000) were seeded on top of the collagen hydrogel cast in 24‐well TW filters. After a maximal 24 h, medium was changed for both IoC and TW to regular Caco‐2 cell culture medium (1% p/s) and were cultured for one or three weeks at 37 °C, 5% CO_2_ with media refreshments every 2–3 days. At day 14, flow was applied to IoCs by a 2D rocking platform (VWR, Breda, The Netherlands) with a speed rate of 1 rotation per minute at a 10° angle or were left static until day 21.

### Immune Cell Isolation and Cell Culture

Buffy coats were obtained from the Dutch blood bank (Sanquin, The Netherlands). All donors were healthy volunteers who provided written informed consent for the use of their materials in scientific research (contract number: NVT0286.00). Human PBMCs were isolated by density‐gradient isolation using Leucosep tubes (Greiner‐Bio, 227288). CD14^+^ monocytes (Miltenyi Biotec, 130‐050‐201) and naive CD4^+^ T cells (Miltenyi Biotec, 130‐094‐131) were isolated via Magnetic Activated Cell Sorting (MACS) according to the manufacturer's protocol. CD14^+^ monocytes were differentiated into monocyte‐derived dendritic cells (moDCs) in 6 days using Granulocyte‐macrophage colony‐stimulating factor (GM‐CSF; 60 ng mL^−1^) (Prospec, CYT221) and IL4 cytokine (Prospec, CYT211; 100 ng mL^−1^) in RPMI 1640 (Lonza, Switzerland) culture medium supplemented with 10% FBS and 1% p/s with media changes every other day. Isolated naive T cells were frozen (−80 °C) in FBS containing 10% DMSO until further use. Naive T cells were cultured in Iscove's Modified Dulbecco's Medium (IMDM) with GlutaMAX (Gibco, 31980‐022) containing 5% FBS, 1% p/s, apo‐transferrin (Sigma–Aldrich, T1147‐100MG; 20 µg mL^−1^) and β‐mercaptoethanol (Gibco, 31350‐010; 50 µm). Purity of CD14^+^ and naive CD4^+^ T cells isolated was determined (Figures S2 and S15, Supporting Information). For all experiments, independent donors were used, with N = 4 in the IoC system and N = 3 in the TW system (N = 2 overlapped in both systems).

### Immunogenicity of IoC Materials

MoDCs were exposed to polydimethylsiloxane (PDMS), PLA, glass, glass with glue, L‐DOPA coated or uncoated MEW half‐pipe scaffolds, and L‐DOPA coated or uncoated wells and were tested on immunogenicity. MoDCs (500 000) in culture medium were pipetted on top of the different materials or were stimulated with a type 2 polarizing DC2 mix (1 µg mL^−1^ prostaglandin E2 (Prospec, P0409‐1MG), 10 ng mL^−1^ IL6 (Prospec, CYT‐213), 25 ng mL^−1^ IL1β (Peptrotech, 200–01b‐10ug), 50 ng mL^−1^ tumor necrosis factor‐α (Prospec, CYT‐223‐a)), with type 1 polarizing lipopolysaccharide (LPS) (a component of the outer membrane of the Gram‐negative bacterium *Escherichia coli* O111:B4, Invivogen; 100 ng.mL^−1^), or were left unstimulated. After 48 h, moDCs were collected and processed for flow cytometry.

### Protein Purification Procedures

Proteins were provided by Wageningen Food & Biobased Research, Wageningen University & Research, The Netherlands. In short, the hen's egg allergen ovalbumin (OVA) was purified over a 3600 mL Source 15Q (Pharmacia) column using a NaCl (0–0.6 m) salt gradient. The collected ovalbumin was dialyzed against demineralized water and freeze dried. Previously described procedures were used to purify the peanut allergen Ara h 2,^[^ ^53^ ^]^ cow's milk allergen β‐lactoglobulin (BLG),^[^ ^54^ ^]^ and the hypo‐allergenic protein Rubisco from spinach.^[^ ^55^ ^]^

### Protein Content Measurements

Ara h 2 and OVA were purified to near‐complete protein purity (data not shown) using the above‐indicated multi‐step chromatographic and dialysis procedures,^[^ ^53^ ^]^ while β‐lactoglobulin (BLG) exhibited a purity exceeding 94% as determined by size‐exclusion chromatography.^[^ ^54^ ^]^ For these respective allergens no additional protein quantifications were performed. Rubisco protein content was tested by Pierce BCA Protein Assay Kit (Thermo Scientific, 23227). LPS content (Table S1, Supporting Information) was determined using the Pierce LAL Chromogenic Endotoxin Quantitation Kit, according to the manufacturer's instructions.

### IEC/moDC Co‐Culture and Exposure to Stimuli

After three weeks, IECs were co‐cultured with moDCs (500 000) embedded in a collagen I hydrogel (1.7 mg mL^−1^) and were added in the basolateral compartment of IoCs and TWs (300 µL). After polymerization, moDC hydrogels were submerged with additional RPMI culture medium (300 µL). IECs were apically exposed to OVA (100 µg mL^−1^), Ara h 2 (100 µg mL^−1^) or BLG (100 µg mL^−1^), hypo‐allergen Rubisco (100 µg mL^−1^), or exposed to toxin A derived from gram‐positive bacterium *C. difficile* (toxin A, List Biological Labs, Inc, 152C; 0.065 µg mL^−1^) combined with LPS (100 ng mL^−1^). MoDC only controls included DC2 mix, LPS (100 ng.mL^−1^), LPS of the highest LPS containing stimuli (Rubisco: 6.08478 ng mg^−1^), or were left untreated. After 48 h, moDC hydrogels were dissolved by Collagenase (Sigma, C0130‐500MG; 1000 units mL^−1^). Supernatant was taken and cells were collected for moDC/T cell co‐culture as well as for flow cytometry.

### MoDC/T Cell Co‐Culture

Naive CD4+ T cells were co‐cultured with allogenic moDCs in a 10:1 ratio, respectively. MoDC/T cell co‐cultures were embedded in collagen I hydrogel (250 µL; 1.7 mg mL^−1^). After polymerization, the hydrogels were submerged with additional T cell medium (250 µL). Co‐cultures were kept for 5 days, without medium refreshments, accompanied by IL2 (Prospec, CYT‐209; 5 ng mL^−1^) and Purified NA/LE Mouse Anti‐Human CD3 (BD Pharmingen, 555336; 150 ng mL^−1^) stimulation. Controls included T cells stimulated with IL2 and anti‐CD3 or left unstimulated. After 5 days, hydrogels were digested as described above, supernatant was collected and T cells were restimulated using PMA (Sigma–Aldrich, 79346‐1MG; 5 ng mL^−1^), Ionomycin (Sigma–Aldrich, I0634‐1MG; 750 ng mL^−1^) in the presence of GolgiPlug (BD Biosciences, 555029) (1 µL mL^−1^) in T cell medium for 5 h at 37 °C 5% CO_2_. T cells were analyzed by flow cytometry.

### Flow Cytometry

MoDCs and T cells were stained for viability, blocked and then extracellular and/or intracellular (after fixation and permeabilization) stained for 30–45 min (Tables S2–S5, Supporting Information). All steps were performed in the dark at 4 °C. Cells were measured using BD FACS Canto II (Becton Dickinson, USA) using BD FACS Diva software (BD Biosciences) for purity of immune cell isolates and CytoFLEX LX (Beckman Coulter, Inc. Brea, CA, USA) using CytExpert software (Beckman Coulter, Inc. Brea, CA, USA) for IEC/moDC and moDC/T cell assays. Data was analyzed using the FlowLogic software (Inivai Technologies, Australia).

### Barrier Integrity by Transepithelial Electrical Resistance (TEER)

TEER values were measured using the Millicell ERS‐2 Volt‐ohmmeter (Millipore) on days 7 and 21 as well as before and after 48 h of allergen exposure. The samples were kept undisturbed in the incubator at least 1 h before measurement.

### Apparent Permeability by FITC‐Dextran Leakage

IEC permeability was assessed using 4 kDa Fluorescein isothiocyanate‐dextran (FITC‐dextran, Sigma, 46944‐100G‐F). 3D printed PLA blocks were placed in the media chambers of the IoC (Figure S4C, Supporting Information) to prevent FITC‐dextran leakage. After 15, 30, 60, 120, and 240 min of FITC‐dextran incubation apically, samples were taken from the basolateral compartment and measured. Apparent permeability was calculated (Equation S1, Supporting Information).

### IEC Cell Performance

Natural cell death of IECs was determined from apical supernatant by using a Cytotoxicity Detection KitPLUS (LDH; Roche, 04744926001) assay. Metabolic activity was measured using PrestoBlue cell viability reagent (Thermo Fisher, A13261). Brush border enzyme activity was subsequently determined by alkaline phosphatase activity, which was quantified using the Amplite Colorimetric Alkaline Phosphatase Assay Kit Yellow Color (AAT Bioquest, 11950).

### Brightfield Microscopy and Immunofluorescent Staining

Brightfield images of fully IEC‐grown half‐pipes and DAPI‐stained cross‐sections were obtained by Nikon Eclipse Ts2, accompanied by a color camera Nikon DS‐Fi3. Collagen I coverage of MEW half‐pipes and morphological IEC characteristics were studied using confocal microscopy (Leica TCS SP8 X, Wetzlar, Germany) (Table S6, Supporting Information). After fixation of IECs, permeabilization, and blocking, cells were stained with primary antibodies targeting the tight junction protein zonula occludens‐1 (ZO‐1). Cells were then stained by secondary antibody, Phalloidin‐iFluor 488 Reagent, and DAPI. Images were analyzed using Leica Application Suite X software or ImageJ.

### Enzyme‐Linked Immunosorbent Assay (ELISA)

Basolateral samples from IEC/moDC and moDC/T cell co‐cultures were analyzed for cytokine and chemokine secretion. IFNγ (Invitrogen, 88–7316), IL4 (Invitrogen, 88–7046), IL10 (Invitrogen, 88–7106), IL12p70 (Invitrogen, 88–7126), IL13 (Invitrogen, 88–7439), TSLP (Invitrogen, 88–7497), IL5 (Biolegend, 430401), CCL20 (R&D, DY360‐05), CCL22 (R&D, DY366), IL8 (R&D, DY208), IL33 (R&D, DY3625B) were measured according to manufacturer's protocol, with minor adjustments: all volumes in the protocol used were halved compared to the original volume (except for the blocking buffer step).

### Statistical Analysis

Results were depicted as mean ± standard error of the mean (SEM). Statistical analysis was performed in GraphPad Prism (version 10.4.1). Data were first tested for normality. When data followed a normal distribution, paired comparisons between two conditions were analyzed using a paired *t*‐test, while datasets with more than two paired conditions were analyzed using a repeated measures (RM) one‐way ANOVA followed by Dunnett's or Tukey's multiple comparisons post‐hoc test. If variances were not equal, data were transformed (sqrt(Y) or log(Y)) and retested. If variances remained unequal, the Geisser‐Greenhouse correction was applied. In case of missing values, a mixed‐effects model with Dunnett's multiple comparisons test was used instead of the RM one‐way ANOVA. For non‐normally distributed data, transformation (sqrt(Y) or log(Y)) was applied to achieve normality. If normality could not be restored, two paired conditions were analyzed using the Wilcoxon signed‐rank test, while more than two paired conditions were analyzed using the Friedman test followed by Dunn's multiple comparisons post‐hoc test. A *p*‐value of ≤ 0.05 was considered statistically significant. ^*^ = *p* ≤ 0.05; ^**^ = *p* ≤ 0.01; ^***^ = *p* ≤ 0.001; ^****^ = *p* ≤ 0.0001.

## Conflict of Interest

The authors declare no conflict of interest.

## Author Contributions

H.S.S. and C.M.L.N. contributed equally to this work. R.J. designed the in vitro study and wrote the manuscript. R.J., H.S.S., and C.M.L.N. performed the experiments and analyzed the data. A.H. supported in MEW printing and printing optimalisation. G.A.H.J. isolated and provided food allergens OVA and Ara h 2 and hypo‐allergen Rubisco. M.G.V. and A.M.G. supported in study design with regard to MEW printing and data interpretation. J.M., S.B‐N, L.E.M.W., and R.M. supported in study design. S.B‐N, L.E.M.W., and R.M, reviewed and edited the manuscript. All authors read and contributed to the final version of the manuscript.

## Supporting information



Supporting Information

Supporting Information

## Data Availability

The data supporting the conclusions of this article will be made available by the authors upon reasonable request.
