# Supplementary material for: Half‐Pipe Melt Electrowritten Scaffolds Support Engineering of an Immunocompetent Hydrogel‐Embedded Intestine‐on‐a‐Chip
Source: Adv Sci (Weinh). 2025 Jul 10;12(37):e07132. doi: 10.1002/advs.202507132 (PMC12499499; doi:10.1002/advs.202507132)
Supplement: Supplementary file 1 — Supporting Information [file ADVS-12-e07132-s001.pdf]

ADVANCED SCIENCE
Open Access

Supporting Information

for *Adv. Sci.*, DOI 10.1002/advs.202507132

Half-Pipe Melt Electrowritten Scaffolds Support Engineering of an Immunocompetent Hydrogel-Embedded Intestine-on-a-Chip

*Robine Janssen, Henrike S. Schulze, Claire M. L. Nelissen, Marta G. Valverde, Andrei Hrynevich, Govardus A. H. de Jong, Anne Metje van Genderen, Jos Malda, Shanna Bastiaan-Net, Linette E. M. Willemsen and Rosalinde Masereeuw**

Biological and Biomedical Sciences Data Reporting Checklist

At Wiley, we wish to improve the reproducibility and data quality of all our published content according to the FAIR principles. Please fill our this form as part of the submission process for all original research articles reporting investigations within the fields of biological and biomedical sciences.

For further information, please see Wiley's data sharing policy.

1. Materials, Systems, and Methods

In the following table, please select all of the materials/methods that are relevant to your work in the left column (by checking the box) . Then, for each of these checked boxes in the left column, please provide all the requested information in the right column.

What materials, methods, systems are used?	Please fill in all that apply
☐ Newly created materials	The structure, synthesis methods, characterization of the compounds with sufficient experimental details to ensure reproducibility is provided ☐ in section:
☐ Nanomaterials, nanoparticles	Size/dimensions and shape (before and after cargo loading, if appropriate):<br>Size dispersity or aggregation:<br>Zeta potential: Density:<br>Max. Cargo loading amount: Max. release amount:<br>Targeting performance is provided ☐ in section:<br>Label intensity per nanoparticle/nanomaterial unit:<br>Characterization was performed in a solution that mimics a biological environment ☐
☐ Therapeutic agents, Drugs, drug-like compounds, probes, inhibitors	Toxicity analysis is provided ☐ in section:<br>Selectivity analysis is provided ☐ in section:<br>Formulation, including concentration and vehicle
☐ Pan-Assay Interference Compounds (PAINs)	This manuscript reports a potential PAIN ☐ The results of an appropriate control analysis have been provided in section:
☐ DNA, RNA, or peptide sequences	Sequences, accession or catalogue numbers of all DNA and RNA molecules, including plasmids, used are provided ☐ in section
☑ Proteins	Sufficient information to identify the proteins studied (e.g. protein name, organism of origin, accession number, and any modifications) are provided ☑ in section: Short in Experimental Section, detailed in Supportin

☑ **Antibodies**	Supplier name, lot number, clone name, and catalogue number for commercially available antibodies are provided ☑ in section: Supporting Information Table<br><br>The validation of each newly created antibody for the species and application, including any validation statements, relevant citations, and antibody profiles in online databases or repositories are provided ☐ in section:
☐ **Prokaryotes and fungi**	Source, including supplier name and catalogue number for commercially available prokaryotes and fungi are provided ☐<br><br>Genus, species, strain, or RRID are provided ☐
☑ **Eukaryotic cell lines and primary cell cultures**	The cell line source (e.g. supplier name, catalogue number are provided ☑<br><br>Species, strain, clone number, or RRID are provided, where applicable ☑<br><br>Culture history (passage number if available) ☑ sex of origin ☐<br><br>genotype ☐ genetic-modification status ☐ are provided, if applicable<br><br>Cell line is not listed on the ICLAC Register of Misidentified Cell Lines<br><br>For primary cultures only!<br>Details of most recent mycoplasma testing are provided ☑<br><br>The above stated cell line information is provided in section:<br>Experimental Section
☐ **Animals or animal-derived materials**	The ethical approval from the relevant national or local authorities was obtained prior to the research ☐<br>An ethics statement that includes the name of the authority and the approval or accreditation number, if applicable, of the laboratory, project, or investigator is provided in section:<br><br>Ethical approval was not required for the animal experiments reported here ☐<br><br>This study complies with the ARRIVE guidelines ☐<br><br>This study complies with the PHS Policy on Humane Care and Use of Laboratory Animals ☐<br><br>The species, strain, sex, age, developmental stage, genetic-modification status, genotype, and original source, where applicable, are provided ☐ in section:<br><br>The accession number or supplier name (including commercially available or biobank samples), catalogue number, clone number, or RRID are provided ☐<br><br>in section:<br><br>The methods/medication administered to minimize suffering during the experiments, the methods of sacrifice, sample collection, anesthesia, housing, sustenance, and storage conditions are provided ☐ in section:<br><br>Volume, method and location of administration of the drug/probe<br><br>A timeline of the experiments is provided ☐ in section:

☐ **Human research participants**	Ethical approval from the relevant national or local authorities was obtained prior to the research ✔ Please provide an ethics statement that includes the name of the authority and the approval or accreditation number, of the laboratory, project, or investigator: The Buffy coats were purchased from the Dutch blood b[illegible]<br>A statement explaining why ethical approval was not required, including the name of the authority that granted this exemption is provided ☐
☐ **Clinical trials**	This study complies with the WMA Declaration of Helsinki – Ethical Principles for Medical Research Involving Human Subjects ✔<br>This study complies with the Case Report guidelines (CARE) ☐<br>Please provide the clinical-trial registration number and a link to the registration website
☐ **Human tissue samples**	Phase 2 and 3 clinical trials are reported in accordance with the CONSORT guidelines ☐<br>Tumor-marker studies are reported in accordance with the REMARK guidelines ☐<br>Human biospecimens studies are reported in accordance with the BRISQ guidelines ☐
✔ **Human-derived materials** (including blood, urine, feces, and serum; including the testing of sensor or wearable technologies)	This study complies with the NIH Policy for Research involving Human Embryos ☐<br>This manuscript maintains the anonymity of the study participants ✔<br>Relevant demographic variables of the study participants, such as age, sex, gender, and ethnicity are provided ☐ in section:<br>The methods used to assign demographic variables to the study participants are provided ☐ in section:
☐ **Human embryos and/or stem cells**	Terminology that might stigmatize the study participants was avoided ✔<br>Informed, written consent was obtained from all participants or their next of kin ✔<br>Please provide the source of human material used Blood (Buffy Coat)

2. Experimental Design, Data Analysis, and Presentation

In the following table, please select all that apply to your study

	**Please check all that apply**
**Images of gels and/or blots**	Lanes have not been spliced together ☐<br>Lanes from the same gel or blot have been spliced together, and the splicing is indicated in the figure legend and by a line on the figure ☐<br>The corresponding molecular weights are indicated on gels and blots, wherever possible ☐<br>Loading controls were run on the same gel ☐<br>Staining or exposure protocol is reported ☐

**Figures** (general)	All Figures are presented with the highest clarity and resolution ✔<br>All Figures accurately reflect the unprocessed image and unprocessed images will be made available upon request ✔<br>Permissions to reproduce have been obtained for all copyrighted images ✔<br>No identifiable images of persons have been used ✔<br>Identifiable image of persons have been used and a consent to publish was obtained ☐
**Charts/graphs**	Individual data points are shown (whenever possible) ✔<br>Statistical significance is shown ✔<br>Error bars are shown ✔<br>Data-presentation format shows data distribution clearly ✔
**Experimental design**:<br>e.g. Randomization, Blinding, Data Exclusion	Criteria upon which the samples/organisms/participants were allocated into experimental groups are provided ☐ in section:<br>If this allocation was random, the measures used to control covariates are provided ☐ in section:<br>Investigators were blinded to the group allocation during data collection and/or analysis ☐<br>Blinding was not possible or not relevant ✔<br>Criteria for the inclusion or exclusion of experimental units or data points are provided ✔ in section: Supporting Information<br>There were no criteria for the inclusion or exclusion of experimental data ☐<br>No experimental units or data points were excluded from the analysis ☐
**Statistics**	Sample size (n) for each experimental group in each experiment is provided ✔<br>Number and type (i.e. biological or technical) of replicates performed in each experiment and relevant information on the successful attempts at replication is provided ✔<br>An explanation for all findings that were not replicated or cannot be reproduced is provided ☐<br>The name(s) of the statistical test(s) used Mentioned in figure legends and in<br>The statistical test(s) is (are) one-sided ☐ two-sided ✔ both ☐<br>The name and version of the software used to analyze significance GraphPad Prism (version 10.4.1)<br>Significance is defined for each experiment ✔
**Figure legends**<br>(please check whether the required information is provided for all figures where applicable)	The number and type of replicates ✔ Sample size ✔<br>Definition of error bars (i.e. confidence interval, standard deviation, or standard error or the mean) ✔<br>Statistical test used ✔ Definition of significance symbol(s) ✔<br>Definitions of box-and-whisker plot elements ✔ Scale bar size ✔<br>Magnification ✔ Laser/gain/exposure time ✔

This Biological and Biomedical SciencesData Reporting Checklist is made available under the Creative Commons Attribution (CC-BY) License, which permits use, distribution and reproduction in any medium, provided the original checklist is properly cited. Please consult the related article for the license terms applicable to the article.
