# Supplementary material for: Half‐Pipe Melt Electrowritten Scaffolds Support Engineering of an Immunocompetent Hydrogel‐Embedded Intestine‐on‐a‐Chip
Source: Adv Sci (Weinh). 2025 Jul 10;12(37):e07132. doi: 10.1002/advs.202507132 (PMC12499499; doi:10.1002/advs.202507132)
Supplement: Supplementary file 2 — Supporting Information [file ADVS-12-e07132-s002.docx]

Supporting Information

**Half-Pipe Melt Electrowritten Scaffolds Support Engineering of an Immunocompetent Hydrogel-Embedded Intestine-on-a-Chip**

Robine Janssen, Henrike S. Schulze, Claire M. L. Nelissen, Marta G. Valverde, Andrei Hrynevich, Govardus A.H. de Jong, Anne Metje van Genderen, Jos Malda, Shanna Bastiaan-Net, Linette E. M. Willemsen, Rosalinde Masereeuw*

**Detailed Experimental Section 1: Assembly and seeding of the intestine-on-a-chip (IoC****)**

*Sterilization and biofunctionalization of IoC and TW materials:* Post-printing, IoC devices and glass cover slips (Menzel Gläser, 631-0853) were sterilized via UV light for 30 minutes. The IoC devices were bonded to the glass cover slips using sterile medical device epoxy adhesive, Loctite (Henkel, EA M-31CL) and cured overnight, at room temperature (RT). Before cell seeding, polycaprolactone (PCL) half-pipe scaffolds were sterilized using 70% ethanol for 30 minutes and inserted into an ethanol filled sterile IoC device. Excess ethanol was aspirated and the IoC device and the MEW scaffold were washed with 1x Dulbecco’s Phosphate Buffered Saline without calcium and magnesium (PBS, Sigma, D8537) with 5% penicillin and streptomycin (p/s; Sigma-Aldrich) and left to dry for at least 30 minutes under UV light exposure. L-3,4- dihydroxyphenylalanine (L-DOPA, Sigma-Aldrich, D9628-5G; 2 mg mL^-1^) was dissolved in tris(hydroxyethyl)aminomethane (Tris; 10 mM) pH 8.5 buffer at 37°C for 45 minutes. The central compartment (scaffold holder + basolateral compartment) of the IoC system was submerged in sterile filtered (Ministar NML, 16534K, 0.2 μm pore size) L-DOPA (500 µL), resulting in the full submersion of the MEW scaffold. Transwell-like filters (TW) (ThinCert^®^ - 24 well, pores 0.4 µm, transparent PET Membrane, Greiner Bio-One, 662 641) in a 24 wells plate (Greiner Bio-One, 662102) were apically coated with sterile L-DOPA (200 µL). Both systems were coated at 37°C for 5 hours, 5% CO_2_ and washed twice with PBS afterward.

*Collagen casting in IoC system and TWs:* Collagen I hydrogels (1.7 mg mL^-1^) were created as described previously [1]. In short, bicarbonate (Gibco, 25080-094), 10X Minimum Essential Medium (GibcoTM, 11430030), and PureCol^®^ Type I Collagen Solution (Advanced Biomatrix, 5005) were mixed. Subsequently, the collagen I mixture was diluted with regular cell culture medium at a 2:1 ratio (final collagen I concentration of 1.7 mg mL^-1^, pH ~7.4). Upon aspirating all liquids from both the IoC device and the scaffold, collagen I hydrogel (60-75 µL) was applied to the scaffold (most commonly 3 × 25 μL: 2 × via interior IoC device walls, 1 × in the middle of the scaffold), allowing the hydrogel to fully cover the scaffold. In the case of the 24-well TW insert, all liquid was removed and collagen I hydrogel (25 µL) was evenly deposited on top of the filter. Following polymerization at 37°C for approximately 35 min, 5% CO_2_, the hydrogel was submerged in culture medium, supplemented with 5% p/s (5% p/s medium) until cell seeding, to assure sterility after handling.

*Cell seeding and cell culture in the IoC system and TW: Caco*-2 cells were washed, trypsinized and resuspended in 5% p/s medium. 5% p/s medium (450 µL) was added into the central compartment of the IoC device. Then Caco-2 cells (150,000 cells in 100 µL) were added on top of the scaffold and the entire system was tilted 60° on its side with a specific holder (**SF 4D)** at 37°C, 5% CO_2_, for 1 hour. Afterwards, additional Caco-2 cells (150,000 cells in 100 µL) (were kept on ice in the meantime) were added on top of the scaffold and the system was tilted 60° on its other side at 37°C, 5% CO_2,_ for 1 hour. The fully seeded IoC was left in an upright position over night at 37°C, 5% CO_2_. For TWs, Caco-2 cells (59,000 cells in 200 µL) were pipetted on top of the collagen cast 24 wells TW filters while the basolateral compartment was filled with 5% p/s medium (600 µL). Seeded TWs were incubated over night at 37°C, 5% CO_2_. The next day, culture medium was changed for both IoC and TW to regular cell culture medium (1% p/s) and both IoCs and TWs were cultured for one or three weeks at 37 °C, 5% CO_2_. Apical medium of IoCs (1050 µL) and TW (200 µL) and basolateral medium of IoCs (150 µL) and TWs (600 µL) were refreshed every 2–3 days. At day 14, flow was applied to IoCs by incubating them on a 2-dimensional rocking platform (VWR, Breda, The Netherlands) with a speed rate of 1 rotation per minute at a 10° angle or were left static until day 21. After TEER and 4 kDa FITC-dextran measurements (see below), IEC MEW half-pipes were carefully removed. The IEC MEW half-pipe was cut into pieces (length ≈ 2 mm), and TW filters were punched out with a biopsy puncher (⌀ 3 or 4 mm). Both IEC MEW half-pipe slices and IEC TW punch outs were transferred to 96 well plates, washed once with PBS and used for further IEC readout assays.

*Immunogenicity of IoC materials:* All the different 3D printed and assembly materials were prepared for a 24 wells (suspension) culture plate: polydimethylsiloxane (PDMS, SYLGARD™ 184 Silicone Elastomer Kit, Dow, Midland, MI, USA) (2 mm height, ⌀ 15 mm) and glass (Menzel Gläser, ⌀ 15 mm) were autoclaved while PLA (1 mm height, ⌀ 15 mm) and glue (glass slide with a ~ 2 mm wide diagonal line of Loctite medical device epoxy adhesive) were sterilized, by immersion in 70% ethanol for 30 min, followed by 30 minutes of UV light exposure. Afterwards all materials were assembled in the 24 wells plate and the entire plate was sterilized again by 30 minutes UV exposure. MoDCs in RPMI 1640 (Lonza, Switzerland) supplemented with 1% p/s and 10% FBS (500,000 cells in 500 µL; differentiated from frozen PBMCs) were pipetted on top of PDMS, PLA, Glass, and Glass with glue, in 24 wells (suspension) plate. Besides, sterilized and L-DOPA coated or uncoated MEW half-pipe scaffolds (half-pipe pieces of ~0.5 cm in length) and uncoated or L-DOPA coated wells (24 wells suspension plate) were tested on immunogenicity (500,000 moDCs in 500 µL per condition; differentiated from freshly isolated PBMCs). Additional wells for negative control (no stimulation) and positive controls: type 2 stirring DC2 mix (1 µg mL^-1^ prostaglandin E2 (Prospec, P0409-1MG), 10 ng mL^-1^ IL6 (Prospec, CYT-213), 25 ng mL^-1^ IL1β (Peptrotech, 200-01b-10ug), 50 ng mL^-1^ tumor necrosis factor-α (Prospec, CYT-223-a)) or a type 1 stirring lipopolysaccharide (LPS) (100 ng mL^-1^) stimulation were included. After 48h, moDCs were resuspended and collected. Afterwards the wells were washed once with cold PBS to collect the last moDCs. MoDCs were spun down at 300 × g, 4°C for 10 minutes and kept on ice until further processing for flow cytometry.

**Detailed Experimental Section 2: Allergen exposure, IEC/moDC co-culture and moDC/T cell co-culture**

*Protein purification procedures:* Allergens were purified using optimized protocols developed at Wageningen Food & Biobased Research, The Netherlands. The hen’s egg allergen ovalbumin (OVA) was purified with the following procedure: The egg white of 8 eggs was collected, and 10 times diluted with Tris (20 mM, pH 8.0). The egg white suspension was centrifuged (45 min, 17000 × g) and the supernatant was filtered over a paper filter and applied to a 3600 mL Source 15Q (Pharmacia) column (diameter 20 cm, height 12 cm) previously equilibrated with Tris, pH 8.0 (20 mM; loading buffer). After washing with loading buffer, the column was eluted with a 40 L salt gradient of 0–0.6M NaCl in loading buffer (at a flow of 100 mL.min^-1^). Fractions of 400 mL were collected and analyzed for ovalbumin content and purity. The collected ovalbumin was dialyzed against demineralized water and freeze dried. All buffers used were filtered through 0.45 mm Durapore membranes (Millipore, Bedford, MA, USA). The peanut allergen Ara h 2 was purified according to the procedure described before (from runner market-type cultivar) [2]. Cow’s milk allergen β-lactoglobulin (BLG) was purified according to the procedure described previously [3]. The hypo-allergenic protein Rubisco from spinach was purified according to the procedure described before [4]. Based on the stringency of the above-described purification methods, both Ara h 2 and OVA were of near-complete purity with respect to protein content. Consequently, no further protein quantification assays were performed for these two allergens. The purity of Ara h 2 and OVA was evaluated by SDS-PAGE (data not shown), confirming the absence of detectable contaminating proteins. The purity and protein content of the isolated BLG exceeded 94%, as determined by size-exclusion chromatography, in accordance with the methodology described before [3]. The purity of Rubisco was estimated to be higher than 95%, based on SDS PAGE (data not shown) and protein content was determined using a BCA assay (Pierce™ BCA Protein Assay Kit, Thermo Scientific, 23227).

*IEC/moDC co-culture and exposure to stimuli:* After three weeks of IEC culture in either IoCs or TWs, IECs were co-cultured with moDCs embedded in a collagen 1 hydrogel. Hydrogels were prepared as described above. MoDC suspension (500,000 cells in 100 µL) were mixed with collagen I hydrogel (200 µL) and embedded in the basolateral compartment (final collagen I concentration when mixed with moDCs= 1.7 mg mL^-1^). During one iteration of the IoC-moDC experiment and two iterations of the TW-moDC experiment, fewer moDCs were available, necessitating an adjustment in cell concentration (150,000 cells in 100 µL, mixed with 200 µL of collagen I hydrogel). Given that our IEC/immune cell co-culture represents a preliminary pilot study, and the observed cytokine and chemokine production levels were within the same range as those obtained with higher seeding concentrations, we decided to include all iterations in the subsequent analysis. After 35 minutes polymerization at 37C, 5% CO_2_, moDC hydrogels were submerged with moDC culture medium (300 µL). Subsequently, IECs were apically exposed by OVA (100 µg mL^-1^), Ara h 2 (100 µg mL^-1^) or BLG (100 µg mL^-1^) allergens, or Rubisco (100 µg mL^-1^) as hypo-allergen. In addition, IECs were exposed to toxin A from *C. difficile* (toxin A, List Biological Labs, Inc, 152C; 0.065 µg mL^-1^) in combination with lipopolysaccharide (LPS, *E. coli* O111:B4, Invivogen; 100 ng mL^-1^) to distinguish a type 1 immune response. IEC/moDC co-cultures were exposed for 48 hours, at 37 °C, 5% CO_2_. After 48 hours, TEER was measured (see below) and IEC MEW half-pipes were carefully detached from the basolateral moDC containing hydrogel using a sterile (by dipping first in 70% ethanol and subsequently in sterile PBS) thin wire. After removal of the IEC MEW half-pipe and after transferring TW inserts to a new 24 wells plate, basolateral moDC hydrogels were dissolved by Collagenase (Sigma, C0130-500MG; 1000 units.mL^-1^), 30 minutes incubation at 37 °C, 5% CO_2_. Degraded hydrogels were spun down 10 min, 300 × g at 4 °C. Supernatant was collected and stored at -20 °C until further cytokine and chemokine measurements by ELISA. MoDCs were collected and kept on ice until moDC/T cell co-culture as well as for flow cytometry. In addition, one part (50%) of the IEC MEW half-pipe was digested by collagenase to collect possible moDCs in the hydrogel just below the IEC MEW half-pipe to measure by flow cytometry. The other part of the IEC MEW half-pipe was cut into further pieces (length ≈ 2 mm), and TW filters were punched out with a biopsy puncher (⌀ 3 or 4 mm). Both IEC MEW half-pipe slices and IEC TW punch outs were transferred to 96 well plates, washed once with PBS and used for further IEC readout assays.

*MoDC/T cell co-culture:* Naive CD4^+^ T cells were thawed and were co-cultured with allogenic moDCs in a 10:1 ratio (1,000,000 : 100,000 in a 24 wells suspension plate (Greiner, 662102)), respectively. When fewer cells were available (either moDCs or T cells), the volumes were adjusted proportionally while ratios were kept the same (500,000 T cells : 50,000 moDCs in a 48-well suspension plate (Greiner, 677102)). MoDC/T cell co-cultures were embedded in hydrogel (1.7 mg mL^-1^ collagen I; 250 µL), followed by 35 minutes polymerization at 37 °C 5% CO_2_, and subsequently submerged with T cell medium (250 µL). Co-cultures were kept for 5 days, without medium refreshments, accompanied by IL2 (Prospec, CYT-209; 5 ng mL^-1^) and Purified NA/LE Mouse Anti-Human CD3 (BD Pharmingen™, 555336; 150 ng mL^-1^) stimulation. After 5 days, hydrogels were dissolved by Collagenase (1000 units mL^-1^). After 30 minutes incubation at 37 °C 5% CO_2_, degraded hydrogels were centrifuged (300 × g, 10 minutes, 4°C) and supernatant was collected and stored until further measurements by ELISA. T cells were collected and restimulated using PMA (Sigma Aldrich, 79346-1MG; 5 ng mL^-1^), Ionomycin (Sigma Aldrich, I0634-1MG; 750 ng mL^-1^) in the presence of BD GolgiPlug™ Protein Transport Inhibitor (BD Biosciences, 555029) (1 μL mL^-1^) in T cell medium (1 µL/1000 cells) for 5 hours, at 37 °C, 5% CO_2_. After 5 hours of restimulation, T cells were centrifuged (300 × g, 10 min, 4°C), resuspended in fresh medium and added to a 96-well U bottom FACS plate (Falcon, 353910). Cells were kept on ice until further analysis by flow cytometry.

**Detailed Experimental Section 3: Read-outs**

*Flow cytometry:* MoDCs and T cells were transferred to a FACS 96 wells plate (Falcon, 353910), washed with cold PBS (300 × g, 5 min, 4 °C) and stained with Fixable Viability Dye (Thermofisher, 65-0865-14) (1:2000) in PBS for 30 minutes in the dark at 4°C. Subsequently, cells were washed (300 × g, 5 min, 4 °C) with FACS buffer (PBS supplemented with 1% Bovine Serum Albumin Fraction V (BSA, Roche, 10735094001) and UltraPure 0.5M EDTA (2 mM; Invitrogen, 15575-038), followed by a blocking step with Human Fc block (BD Pharmingen, 564220) for 10 minutes at 4 °C in the dark. Then cells were washed again (300 × g, 5 min, 4 °C) and were extracellular stained for 30 minutes at 4 °C in the dark, washed using FACS buffer (300 × g, 5 min, 4 °C), followed by resuspension of cells in FACS buffer. For intracellular staining of T cells (after extracellular staining), T cells were fixed using Intracellular Fixation Buffer (Life Technologies, 00-8222-49) and incubated overnight at 4°C protected from light. Fixed cells were centrifuged (600 × g, 5 min, 4 °C) and washed with Permeabilization Buffer 1X (Life Technologies, 00-8333-56). After centrifugation (600 × g, 5 min, 4 °C) and discarding supernatant, cells were intracellular blocked by Human Fc block in 1x Permeabilization Buffer. After 10 minutes (4°C , protected from light), cells were washed with 1x Permeabilization Buffer (600 × g, 5 min, 4 °C) and subsequently intracellularly stained for 30 minutes (4°C, protected from light). Cells were then washed using FACS buffer (600 × g, 5 min, 4 °C), followed by resuspension of cells in FACS buffer. For all antibodies, corresponding isotype controls were included. Measurements were performed using the BD FACS Canto II (Becton Dickinson, USA) flow cytometry for purity of immune cell isolates and CytoFLEX LX (Beckman Coulter, Inc. Brea, CA, USA) for IEC/moDC/T cell assays. Data was analyzed using the FlowLogic software (Inivai Technologies, Australia). See **Table S2-5** for antibodies and isotype controls.

*Barrier integrity by transepithelial electrical resistance (TEER):* TEER values were measured using the Millicell® ERS-2 Volt-ohmmeter (Millipore) at day 7 and 21. The samples were kept undisturbed in the incubator at least 1 hour before measurement. First, the background resistance was subtracted and then the average resistance of an empty system (IoC or TW with just collagen I hydrogel) was subtracted. Finally, the resistance values were multiplied by surface area (A_TW_= 0.33 cm^2^; A_IoC_= 0.8306 cm^2^) to present the TEER in Ω·cm^2^.

*Apparent permeability by FITC-dextran leakage:* To quantify permeability, a leakage assay using 4 kDa Fluorescein isothiocyanate-dextran (FITC-dextran, Sigma, 46944-100G-F) was conducted. Negative controls with IoCs and TWs containing only collagen I hydrogel were included. 3D printed PLA blocks were placed in the apical media side chambers of the IoC **(SF 4C)**. Caco-2 culture medium was replaced with DMEM without phenol red (Gibco, 21041033) containing the same supplements as the standard Caco-2 medium. The systems were allowed to equilibrate at 37°C for at least 1 hour. Then, 4 kDa FITC-dextran solution was added to the apical compartment of the IoCs (V_Apical_= 150 µL, V_basolateral_= 350 µL; 4 kDa FITC-dextran apical concentration=1.07 mg mL^-1^) and TW (V_Apical_= 200 µL, V_basolateral_= 600 µL; 4 kDa FITC-dextran apical concentration=1.6 mg mL^-1^). After 15, 30, 60, 120, and 240 minutes of incubation, samples were taken from the basolateral compartment (and refilled) and transferred into a black flat bottom 96-well assay plate (Costar, 3915). The fluorescence of the samples was measured with excitation at 475 nm and emission at 520 nm (emission filter 500-550). The Apparent permeability ($P_{app} (cm s^{-1})$; Equation 1) was calculated where $dQ$ (nmol) is the amount of 4 kDa FITC-dextran present in the basolateral compartment as a function of $dt$ which represents the time (s), $A$ is the cell growth surface area (cm^2^) and $C_{0}$ is the apical initial concentration (nmol L^-1^) of the 4 kDa FITC-dextran. Basolateral concentrations were corrected for cumulative dilution due to sampling over time. Negative $P_{app}$ values were set to zero in the final figures. In cases where structural defects, such as holes in the half-pipe channels, were observed after (one or) three weeks of culture, the affected samples were excluded from the experiment, and their data were omitted from the final analysis. Although collagen distribution often appeared uniform upon visual inspection and microscopy, the true quality of the coating and cell seeding could only be reliably assessed during cell culture, when contrast under the microscope revealed subtle imperfections. Minor defects in the collagen layer occasionally led to incomplete monolayer formation, allowing cells to migrate through the half pipe and transfer to the basolateral compartment. To preserve the integrity of the dataset, such chips were systematically excluded from analysis.

$P_{app} (cm s^{-1})=\frac{dQ}{dt}\times\frac{1}{A\times C_{0}}$ (1)

*Cell viability by LDH assay:* Media samples were collected after 48 hours of culture at the three-week timepoint, after TEER measurements. Positive controls were obtained by incubating a slice of the half-pipe scaffold with Triton X-100 (Merck, 1.08603.1000), at a 100x dilution, for 1 hour. All samples were frozen at -80°C until use. The Cytotoxicity Detection KitPLUS (LDH; Roche, 04744926001) was used following the manufacturer's instructions. In short, the supernatant (25 µL) was combined with LDH detection solution (25 µL) and incubated for 15 minutes at RT protected from light. Absorbance was measured according to the manufacturer’s protocol, or measured at 405 nm. After subtracting the blank, the values were adjusted to account for both cell growth area (A_TW_= 0.33 cm^2^, A_IoC_= 0.8306 cm^2^) and volume difference in the apical compartment between IoC and TW (V_IoC_ = 1050 µL, V_TW_ = 200 µL).

*Metabolic activity by PrestoBlue^TM^ assay:* The samples in the 96-well plate were incubated in phenol red-free DMEM (100 µL) with PrestoBlue^TM^ reagent (Thermo Fisher, A13261) (10x dilution), according to the manufacturer's protocol. For the negative control, Triton X-100 (Merck, 1.08603.1000) was added to the mix, at a 100x dilution. After 1 hour of incubation at 37°C and 5% CO_2_, samples (75 µL) were collected and fluorescence was measured at an excitation wavelength of 520 nm and an emission wavelength of 590 nm (emission filter 580-640). After subtracting the blank, the values were normalized to the cell growth area, which was calculated based on the sizes of TW biopsy punches and MEW half-pipe segments. The surface area (A) was determined for MEW half-pipe segments using Equation (2):

$A=0.75 \times(2\pi rh)$ (2)

Where for MEW half-pipes the *r* = 1.5 mm, ℎ represents the width of the MEW half-pipe segment, and the factor 0.75 accounts for the non-complete circular geometry of the scaffold. For TWs biopsy punches, Equation (3) was used to calculate surface area (A):

$A=\pi r^{2}$ (3)

*Brush border enzyme activity by alkaline phosphatase activity assay:* Alkaline phosphatase activity was used as an enterocyte differentiation marker and was quantified using the Amplite® Colorimetric Alkaline Phosphatase Assay Kit Yellow Color (AAT Bioquest, 11950), according to the manufacturer's protocol with slight modifications. In short, samples were incubated (in a total volume of 100 µL), consisting of a 1:1 mixture of Hank’s Balanced Salt Solution (HBSS; Gibco, 14025-100) and alkaline phosphatase working solution (prepared by mixing pNPP stock solution with assay buffer at a ratio of 1:100), at 5% CO_2_, 37°C for 30 minutes. Subsequently, supernatant was transferred to a new 96-well plate and absorbance was measured at 405 nm. Samples were corrected for surface area of MEW half-pipe segments and TW biopsy punches, as described above for PrestoBlue^TM^.

*Microplate Reader:* Absorbance, fluorescence and optical densities were measured using GloMax® Discover Microplate Reader (Promega, USA). If applicable, final concentrations were calculated from calibration curves in each plate using GloMax® software.

*Brightfield microscopy and immunofluorescent staining:* Brightfield images of fully IEC-grown half-pipes and DAPI-stained cross-sections were obtained by Nikon Eclipse Ts2 (at 4x magnification), accompanied by a color camera Nikon DS-Fi3. As the samples were too large to capture in a single image, multiple images were taken and subsequently merged into one overview image. To investigate IEC-layer integrity, phenotypical and morphological characteristics, an immunofluorescence staining was performed and imaged using confocal microscopy (Leica TCS SP8 X, Wetzlar, Germany) (at 4x and 10x magnification). Laser, gain and exposure time were kept similar between conditions. Cells were fixed using Formaldehyde 4% (VWR International, 9713.1000) for 20 minutes at RT and permeabilized (0.3% Triton X-100 in PBS) for 10 minutes at RT. To prevent non-specific binding of antibodies, cells were incubated with block solution (2% BSA in PBS). Cells were incubated with primary antibodies, diluted in block solution, at RT for 1 hour. Primary antibodies targeting collagen I (to test for collagen coverage of the MEW scaffolds) and tight junction protein, zonula occludens-1 (ZO-1) were used. This was followed by 1 hour at RT secondary antibody incubation. During this step also Phalloidin-iFluor 488 Reagent was added to stain the actin filaments. This was followed by staining of the nuclei, by using DAPI for 8 minutes. Between each step the samples were washed twice with washing buffer (PBS with 1:1000 Tween-20). Images were further analyzed using Leica Application Suite X software or ImageJ (National Instruments). For all antibodies used, see **Table S6**.

**Table S1.** LPS content results of allergens and hypo-allergens measured by the Pierce LAL Chromogenic Endotoxin Quantitation Kit.

| **Name** | **LPS (EU.mg^-1^ protein)** | **LPS (ng.mg^-1^ protein)** |
| --- | --- | --- |
| Hen’s egg allergen Ovalbumin (OVA) | 3.01E-01 | 3.01E-02 |
| Cow’s milk allergen β-lactoglobulin (BLG) | 1.28E+01 | 1.28E+00 |
| Peanut allergen Ara h 2 | 5.17E-03 | 5.54E-04 |
| Rubisco (from spinach) | 6.08E+01 | 6.08E+00 |

**Table S2.** List of moDC antibodies that were used.

| **Antibody name** | **Target** | **Fluorophore** | **Brand** | **Cat. nr.** | **Dilution** |
| --- | --- | --- | --- | --- | --- |
| Fixable Viability Dye eFluor™ 780 | Dead cells | APC-Cy7 | Thermo Fisher | 65-0865-14 | 2000× |
| CD14 Monoclonal Antibody (61D3), PerCP-Cyanine5.5 | CD14 | PerCP-Cyanine5.5 | Thermo Fisher | 45-0149-42 | 80× |
| V450 Mouse Anti-Human HLA-DR | HLA-DR | V450 (Pacific Blue) | BD Biosciences | 561359 | 80× |
| APC Mouse Anti-Human CD209 | CD209 | APC | BD Biosciences | 551545 | 80× |
| CD80 (B7-1) Monoclonal Antibody (2D10.4) FITC | CD80 | FITC | Thermo Fisher | 11-0809-42 | 80× |
| CD86 (B7-2) Monoclonal Antibody (IT2.2), PE-Cyanine7 | CD86 | PE-Cyanine7 | Thermo Fisher | 25-0869-42 | 1280× |
| PE Mouse Anti-Human OX40 Ligand (CD252) | OX40L | PE | BD Biosciences | 558164 | 80× |
| Brilliant Violet 510™ anti-human CD197 (CCR7) Antibody | CCR7 | BV510 (AmCyan) | BioLegend | 353231 | 20× |

**Table S3.** List of isotype antibodies that were used, corresponding to the moDC antibody list.

| **Isotype name** | **Target** | **Fluorophore** | **Brand** | **Cat. nr.** | **Dilution** |
| --- | --- | --- | --- | --- | --- |
| Mouse IgG1 kappa Isotype Control (P3.6.2.8.1), PerCP-Cyanine5.5 | N.A. | PerCP-Cyanine5.5 | Thermo Fisher | 45-4714-82 | 160× |
| V450 Mouse IgG2a, κ Isotype Control | N.A. | V450 (Pacific Blue) | BD Biosciences | 560550 | 80× |
| APC Mouse IgG2b κ Isotype Control | N.A. | APC | BD Biosciences | 555745 | 38,8× |
| Mouse IgG1 kappa Isotype Control (P3.6.2.8.1), FITC | N.A. | FITC | Thermo Fisher | 11-4714-81 | 200× |
| Mouse IgG2b kappa Isotype Control (eBMG2b), PE-Cyanine7 | N.A. | PE-Cyanine7 | Thermo Fisher | 25-4732-81 | 10240× |
| PE Mouse IgG1, κ Isotype Control | N.A. | PE | BD Biosciences | 555749 | 20× |
| Brilliant Violet 510™ Mouse IgG2a. κ Isotype Ctrl Antibody | N.A. | BV510 (AmCyan) | BioLegend | 400267 | 10× |

**Table S4.** List of CD4+ T cell antibodies that were used.

| **Antibody name** | **Target** | **Fluorophore** | **Brand** | **Cat. nr.** | **Dilution** |
| --- | --- | --- | --- | --- | --- |
| Fixable Viability Dye eFluor™ 780 | Dead cells | APC-Cy7 | Thermo Fisher | 65-0865-14 | 2000× |
| CD4 Monoclonal Antibody (OKT4 (OKT-4)), PerCP-Cyanine5.5, eBioscience™ | CD4 | PerCP-Cy5.5 | Thermo Fisher | 45-0048-42 | 80× |
| BD Pharmingen™ Alexa Fluor® 488 Mouse Anti-Human CD183 | CD183 (CXCR3) | AF488 (FITC) | BD Biosciences | 558047 | 40× |
| CD294 (CRTH2) Monoclonal Antibody (BM16), APC, eBioscience™ | CD294 (CRTH2) | APC | Thermo Fisher | 17-2949-42 | 10× |
| Brilliant Violet 421™ anti-human IL10 Antibody | IL10 | BV421 (Pacific Blue) | BioLegend | 501421/501422 | 160× |
| PE/Cyanine7 anti-human IFNγ Antibody | IFNy | PE-Cy7 | BioLegend | 502527 | 20× |
| IL13 Monoclonal Antibody (85BRD), PE, eBioscience™ | IL13 | PE | Thermo Fisher | 12-7136-42 | 80× |
| Brilliant Violet 510™ anti-human IL4 Antibody | IL4 | BV510 (AmCyan) | BioLegend | 500835 | 20× |

**Table S5.** List of isotype antibodies that were used, corresponding to the CD4^+^ T cell antibody list.

| **Isotype name** | **Target** | **Fluorophore** | **Brand** | **Cat. nr.** | **Dilution** |
| --- | --- | --- | --- | --- | --- |
| Mouse IgG2b kappa Isotype Control (eBMG2b), PerCP-Cyanine5.5, eBioscience™ | N.A. | PerCP-Cy5.5 | Thermo Fisher | 45-4732-82 | 320× |
| Mouse IgG1 kappa Isotype Control (P3.6.2.8.1), Alexa Fluor™ 488, eBioscience™ | N.A. | AF488 (FITC) | BD Biosciences | 53-4714 | 40× |
| Rat IgG2a kappa Isotype Control (eBR2a), APC, eBioscience™ | N.A. | APC | Thermo Fisher | 17-4321-81 | 20× |
| Brilliant Violet 421™ Rat IgG1, κ Isotype Ctrl Antibody | N.A. | BV421 (Pacific Blue) | BioLegend | 400429 | 200× |
| PE/Cyanine7 Mouse IgG1. κ Isotype Ctrl Antibody | N.A. | PE-Cy7 | BioLegend | 400125 | 80× |
| Mouse IgG2b kappa Isotype Control (eBMG2b), PE, eBioscience™ | N.A. | PE | Thermo Fisher | 12-4732-81 | 640× |
| Brilliant Violet 510™ Rat IgG1. κ Isotype Ctrl Antibody | N.A. | BV510 (AmCyan) | BioLegend | 400435 | 20× |

*Immunofluorescent (primary and secondary) antibodies:*

**Table S6.** Confocal microscopy primary and secondary antibodies.

| **Name** | **Target** | **Fluorophore** | **Brand** | **Cat. nr.** | **Dilution** |
| --- | --- | --- | --- | --- | --- |
| Anti-ZO1 tight junction protein antibody (host species Rabbit) | Zonula occludens-1 (ZO-1) | - | Abcam | ab216880 | 100× |
| Collagen I Monoclonal Antibody (COL-1) (host species Mouse) | Collagen I | - | Invitrogen | MA1-26771 | 100× |
| Donkey anti-Mouse IgG (H+L) Highly Cross-Adsorbed Secondary Antibody, Alexa Fluor™ 594 | Mouse | Alexa Fluor™ 594 | Invitrogen | A-21203 | 200× |
| Goat anti-Rabbit IgG (H+L) Highly Cross-Adsorbed Secondary Antibody, Alexa Fluor™ 647 | Rabbit | Alexa Fluor™ 647 | Invitrogen | A-21245 | 300× |
| Phalloidin-iFluor 488 Reagent | Actin filaments |  | Abcam | ab176753 | 1000× |
| DAPI | DNA |  | Invitrogen | D3571 | 1000× |

**Supporting Figures**


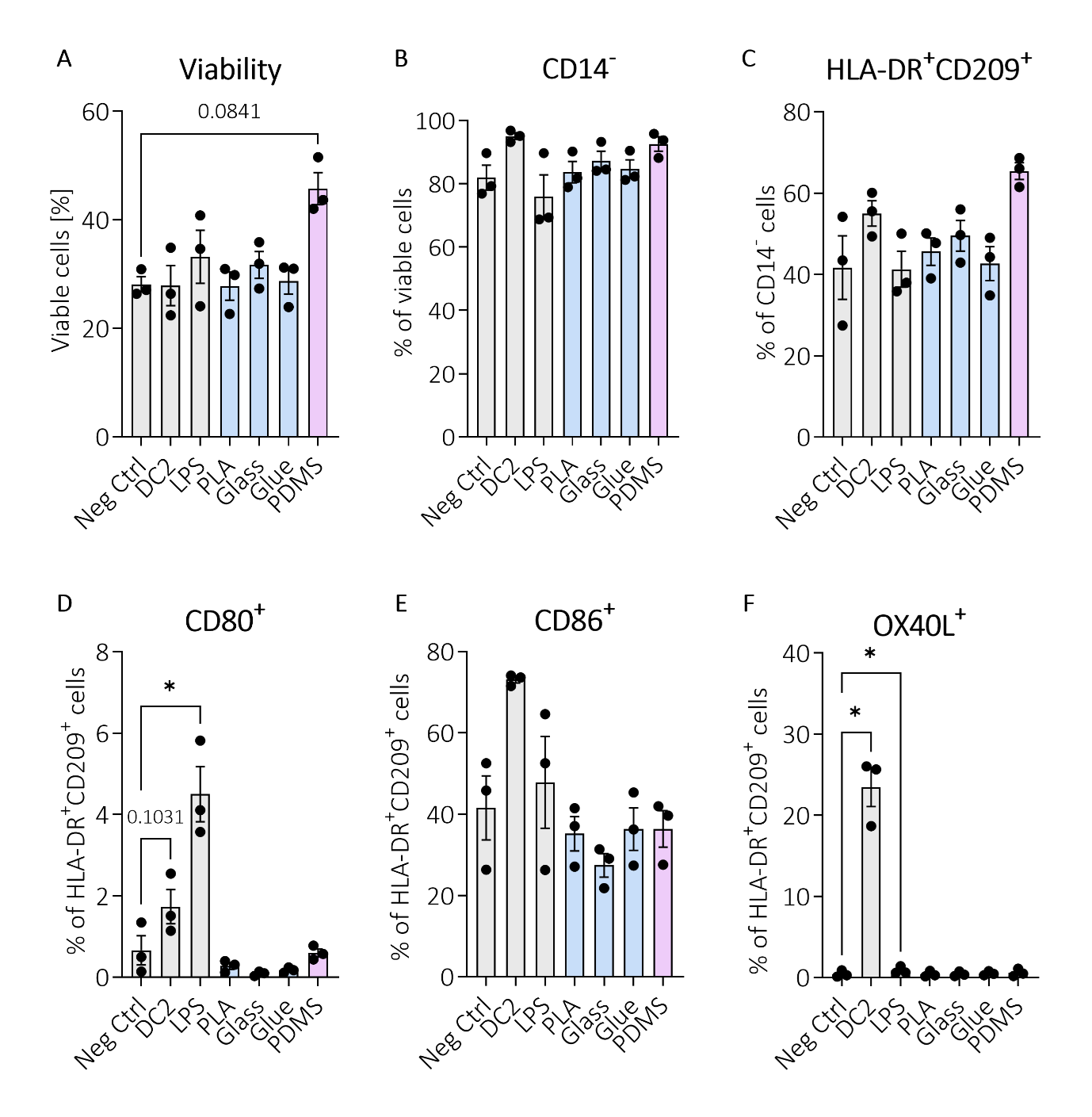
**Figure S1.** Effect of all tested IoC materials, including PDMS, on moDC activation. CD14^+^ monocytes were isolated from frozen PBMCs of three independent donors (N=3). Monocytes were differentiated in 6 days to monocyte-derived dendritic cells (moDCs) and subsequently exposed to DC2, LPS, one of the IoC materials or were left untreated for 48 hours. (A) MoDC viability, (B) CD14^-^ cells, (C) HLA-DR^+^CD290^+^ cells, and activation markers (D) CD80, (E) CD86, and (F) OX40L were measured by flow cytometry. Neg Ctrl= negative control, DC2= media with type 2 stirring DC2 mix (TNFα + IL1β+ IL6 + prostaglandin E2) supplementation, LPS = media with type 1 stirring lipopolysaccharide supplementation, PLA = Polylactic Acid (PLA) filament of the IoC device, Glass = Glass coverslip as base for the IoC, Glue = Loctite adhesive on glass coverslip. PDMS = Polydimethylsiloxane, taken along as PDMS is widely used in organ-on-a-chip systems. Data was statistically tested using repeated measures one-way ANOVA with Geisser-Greenhouse correction with Dunnett's post hoc, or Friedman's test with Dunn's post hoc. All conditions were tested against the negative control only. Error bars represent mean ± SEM, * = p ≤ 0.05.

**
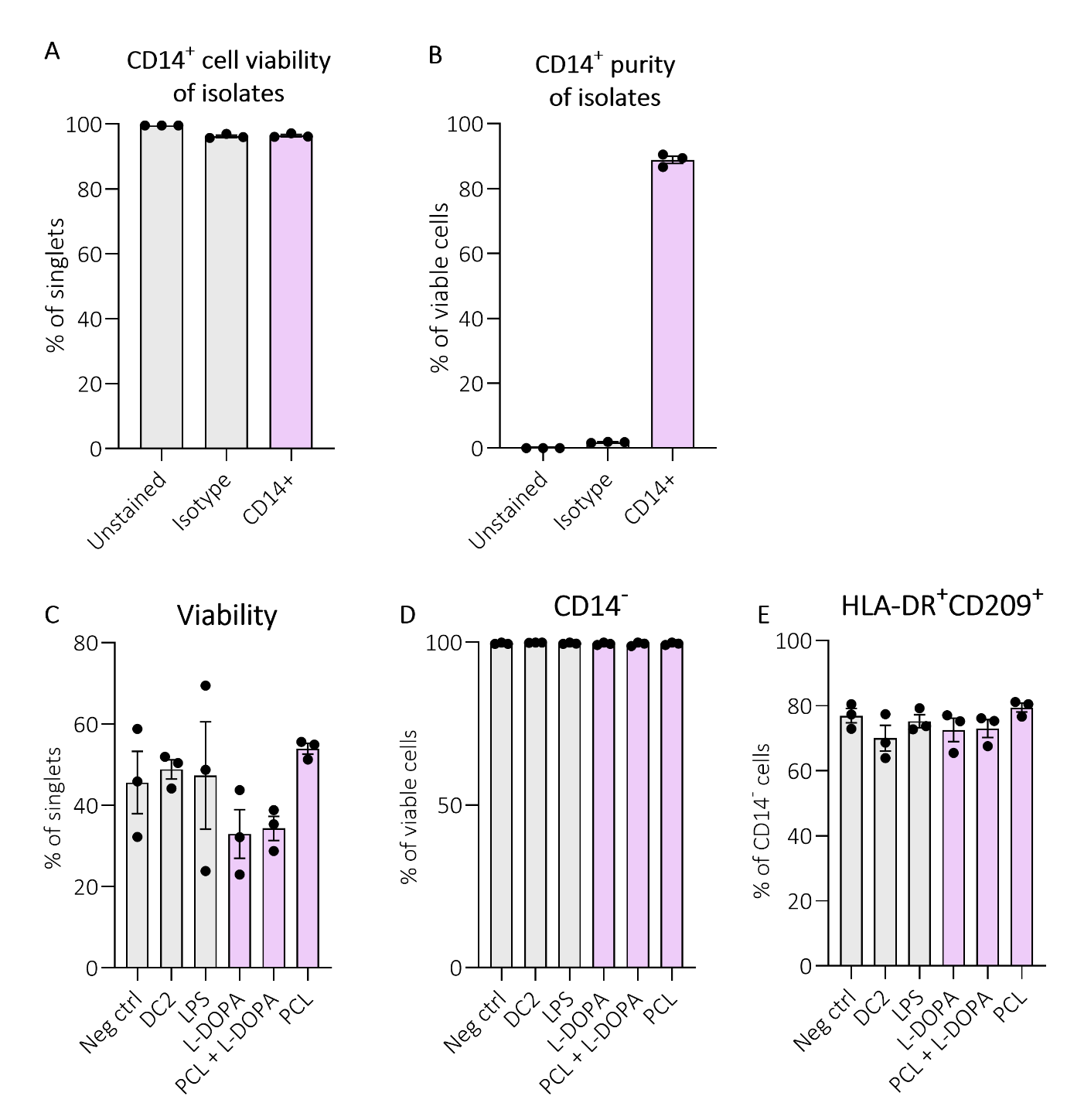
Figure S2**. Effect of PCL fibers and biofunctionalization of PCL fibers on moDC activation. CD14^+^ monocytes were isolated from freshly isolated PBMCs of three independent donors (N=3). Monocytes were differentiated in 6 days to monocyte-derived dendritic cells (moDCs) and subsequently exposed to DC2, LPS, PCL fibers, L-DOPA coated PCL fibers, L-DOPA coated wells or were left untreated. (A) Viability of the CD14^+^ monocyte isolates and (B) CD14^+^ purity of the CD14^+^ monocyte isolates after MACS sorting measured by flow cytometry. (C) Viability of moDCs after 48 hours of exposure to the different materials or stimuli, (D) CD14^-^ cells and (F) HLA-DR^+^CD290^+^ cells assessed by flow cytometry. Neg Ctrl = negative control, DC2 = media with type 2 stirring DC2 mix (TNFα + IL1β + IL6 + prostaglandin E2) supplementation, LPS = media with type 1 stirring lipopolysaccharide supplementation, PLA = Polylactic Acid (PLA) filament of the IoC device, Glass = Glass coverslip as base for the IoC, Glue = Loctite adhesive on glass coverslip. Data (C-E) was statistically tested using repeated measures one-way ANOVA with Dunnett's post hoc. All conditions were tested against the negative control only. Error bars represent mean ± SEM.

**
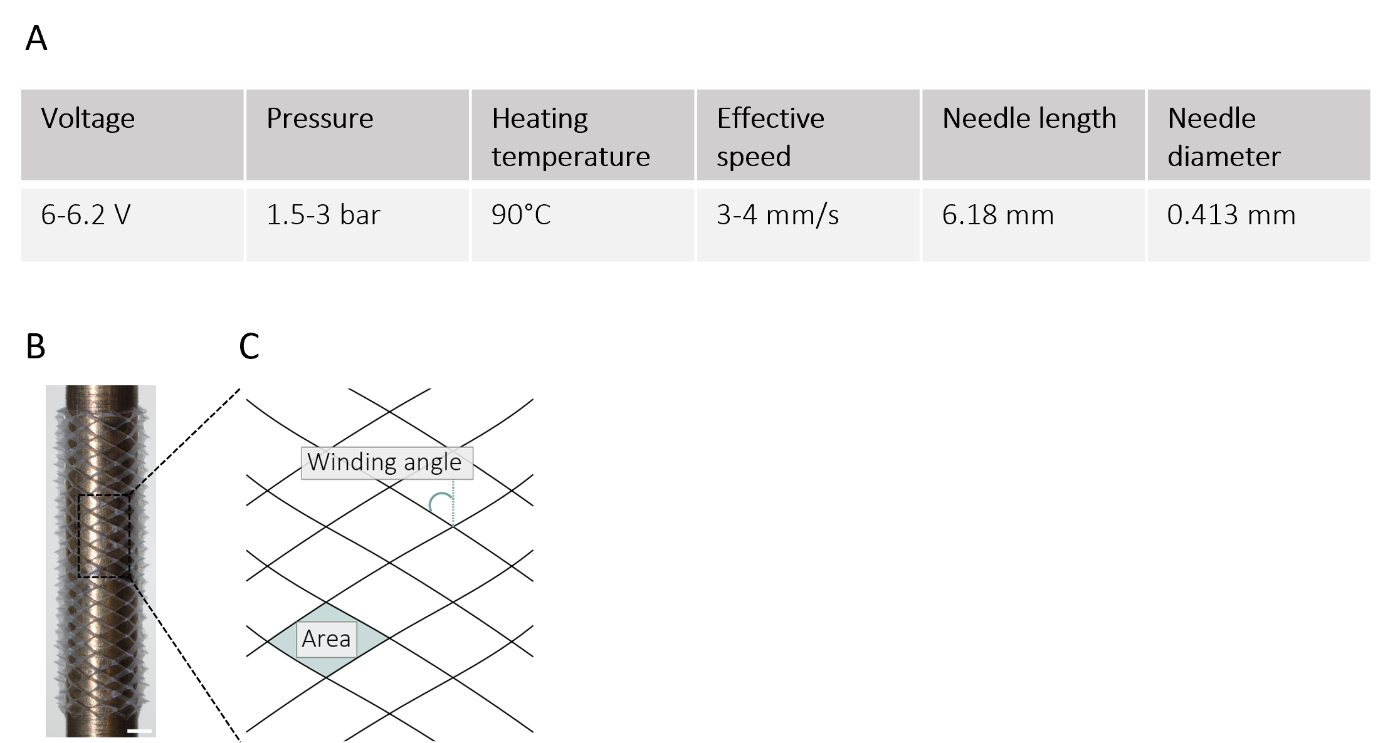
Figure S3.** MEW printing and characterization. (A) Table including the used MEW printing parameters to create half-pipe structures. (B) Representative stereomicroscopy image showing the top view of a MEW half-pipe printed on a 3 mm-wide mandrel (scale bar = 1 mm). This image was previously presented in Figure 2B. (C) Schematic overview of the parameters (winding angle and pore size) characterized in the printed MEW half-pipe scaffolds.


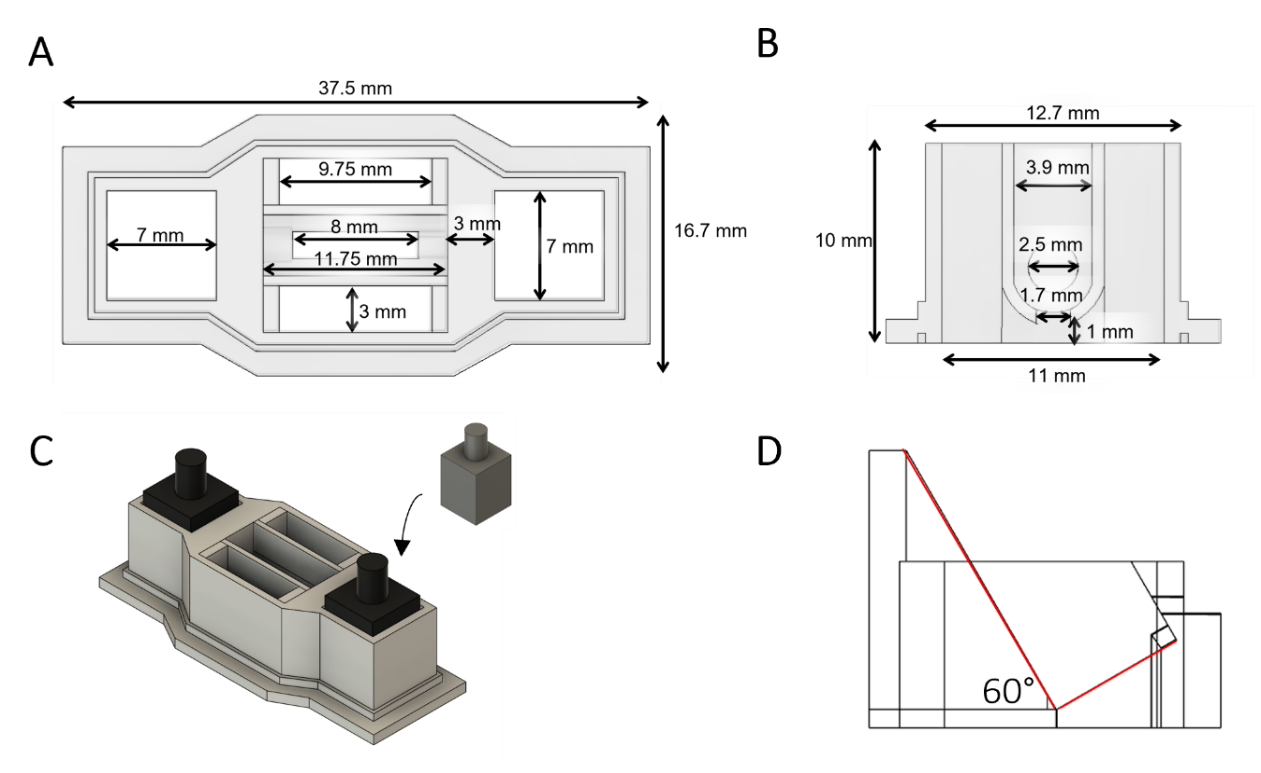


**Figure S4**. IoC system line drawing (A) top view and (B) cross section with dimensions. (C) 3D digital rendering of the IoC (angled top view) and digital rendering of the blocks inserted in the media chambers, to facilitate apparent permeability measurements of IoC. Blocks were printed from PLA with the same settings as IoCs. (D) Schematic drawing of the PLA holder utilized to tilt the IoC at 60° angle during cell seeding in a petri dish. The holder was designed in Fusion 360 (Autodesk) and printed from PLA with 20% infill and 0.3 mm (fast) resolution. The holder was used during cell seeding to allow the IoC devices inside of sterile petri dishes (⌀ 9 cm) to be tilted at a 60° angle (each side) so the cells could settle on (both) the side and not just in the middle of the half-pipe scaffold, allowing a full monolayer to form within one week.


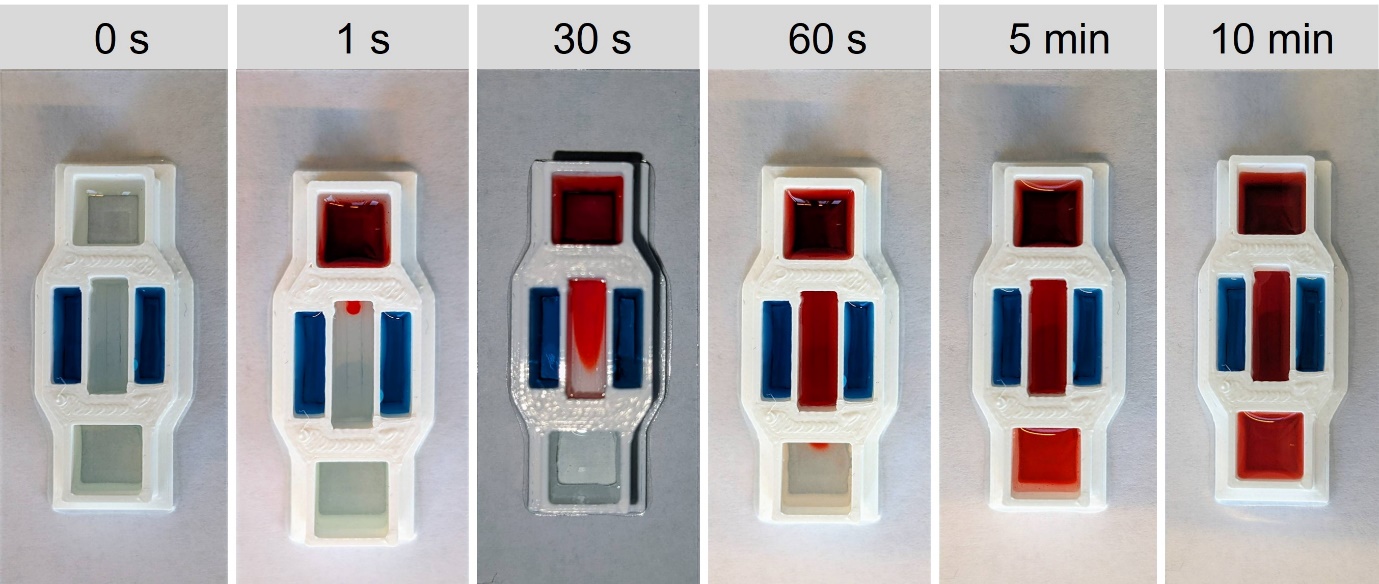


**Figure S5.** Passive fluid flow simulation in the IoC. Fluid flow in the PLA IoC system, visualized by food color (HQ online, 12_PACK_BBBBFGOPRRW). To observe fluid flow in the apical compartment the connection to the basolateral compartment was sealed with a PLA layer during printing. The IoC was placed on a 2-dimensional rocking platform with a speed of 1 rotation per minute at an angle of 10° to initiate fluid flow. Images were taken at 0 s, 1 s, 30 s, 60 s, 5 minutes and 10 minutes (with a Pixle 4a (Google)). The basolateral compartment was filled with blue food color for better contrast. The experiment was performed once (N=1).


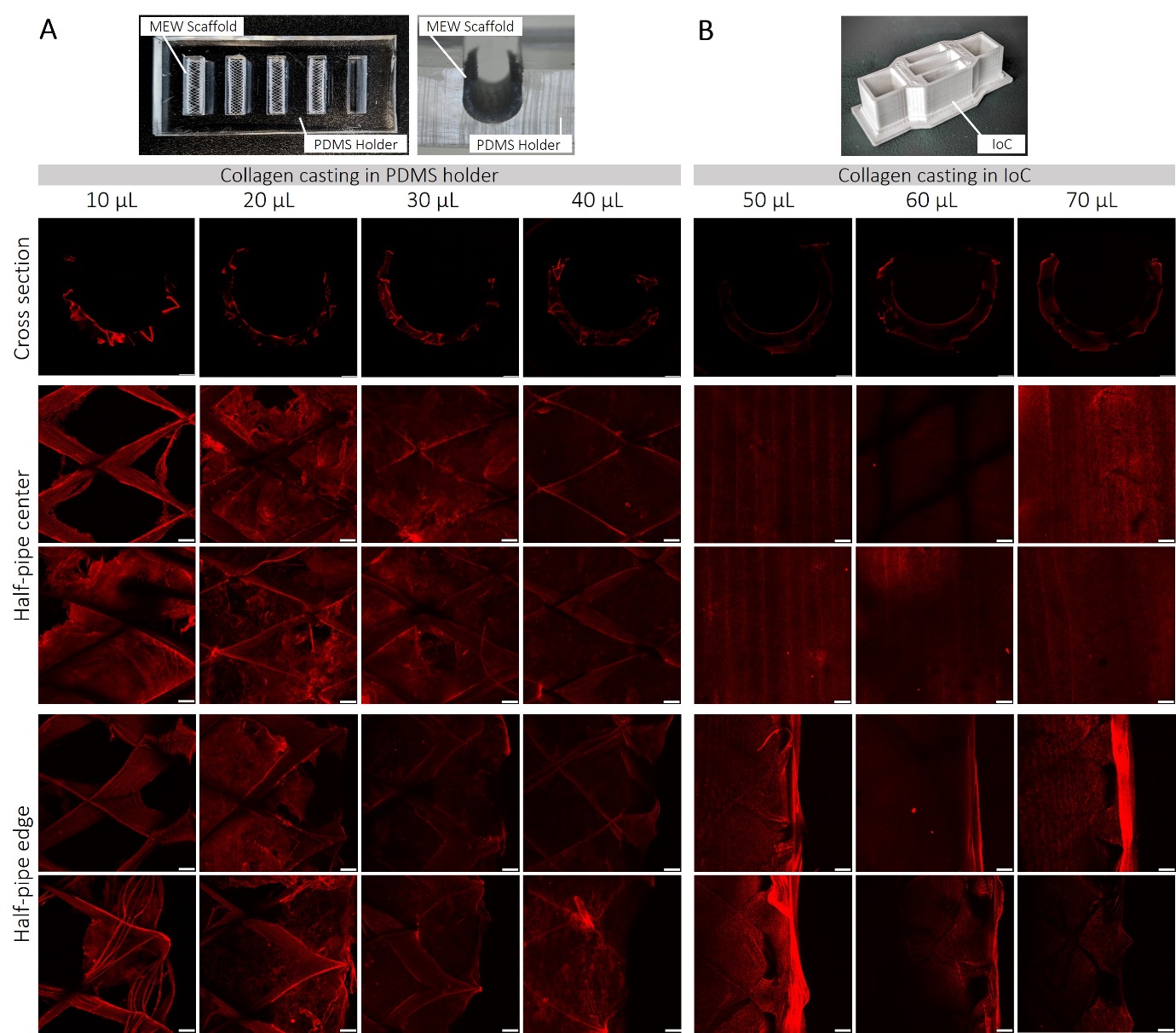


**Figure S6.** Optimization of different amounts of collagen casting in the PDMS holder or IoC. The initial collagen casting experiments (10-40 µL) were conducted in a specifically designed PDMS holder. The PDMS holder was optimized to perfectly fit the MEW scaffold in length width and curvature and created by casting PDMS in a custom-made 3D printed mold. The curvature of the mold is identical to the curvature of the IoC. (A) Macroscopic photos of, on the left, the PDMS scaffold holder top view with inserted MEW scaffolds and on the right the PDMS holder cross section with inserted MEW scaffold. (B) Collagen casting experiments conducted directly in the IoC (50, 60 and 70 µL of collagen). Collagen coverage was assessed by confocal microscopy (z-stacks) after anti-collagen I antibody staining of collagen cast MEW half-pipe with different collagen volumes (10, 20, 30, 40, 50, 60 and 70 µL). (A-B) Top to bottom: cross section of the collagen cast MEW half-pipe (compiled images, each at 4x magnification), duplicates of the bottom view of the scaffold center (at 10x magnification), and duplicates of the bottom view of the edge of the scaffold (at 10x magnification). As samples to obtain cross sections of the half-pipe were larger than the microscopic viewing area, multiple images were taken (at 4x magnification) and compiled into one image. Scale bars cross sections half-tube = 500 µm, scale bars half-pipe center and edge = 100 µm. Cross sections represent representative images of the technical duplicates, and images of the center and edge show both technical duplicates.

**
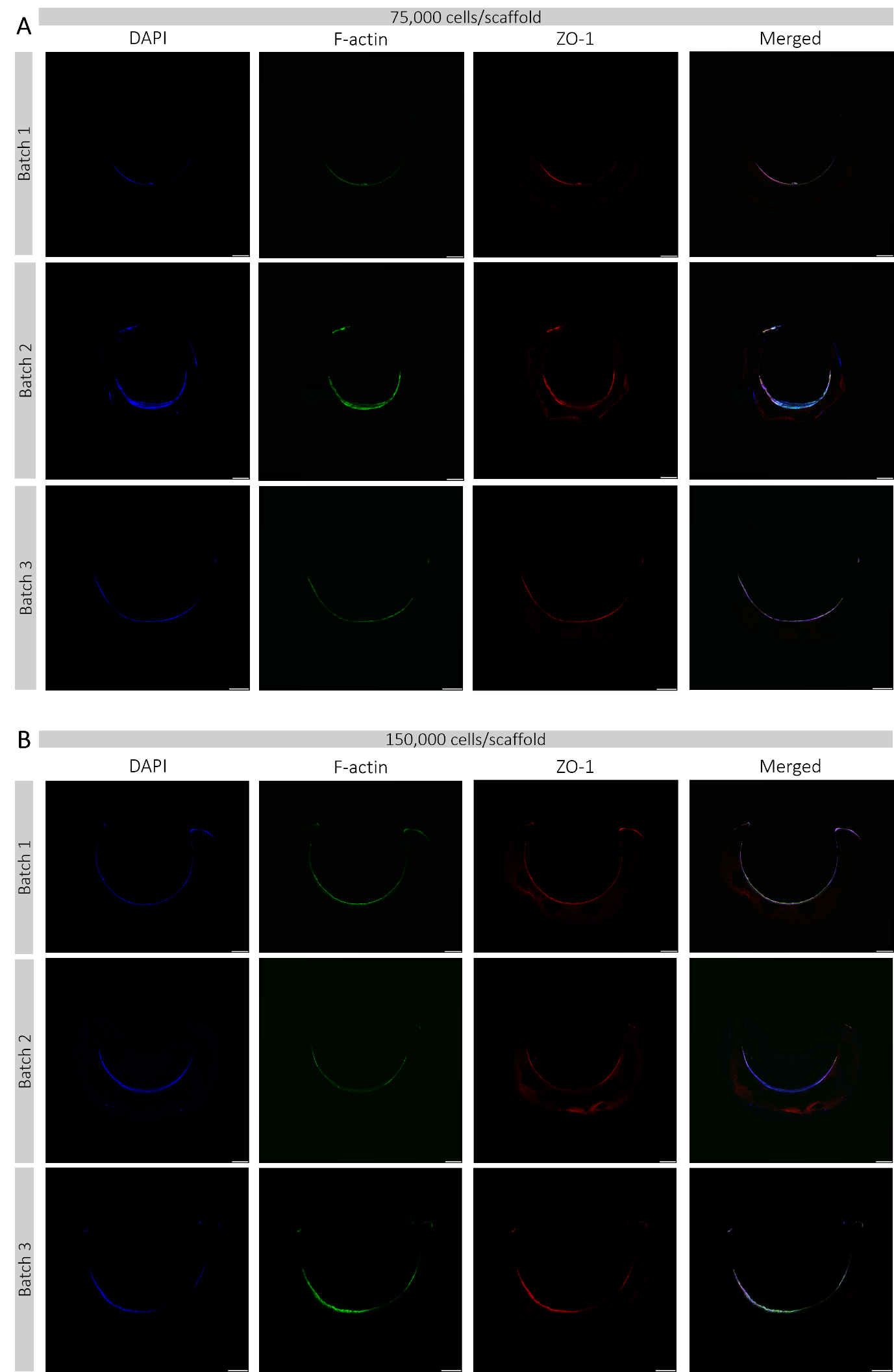

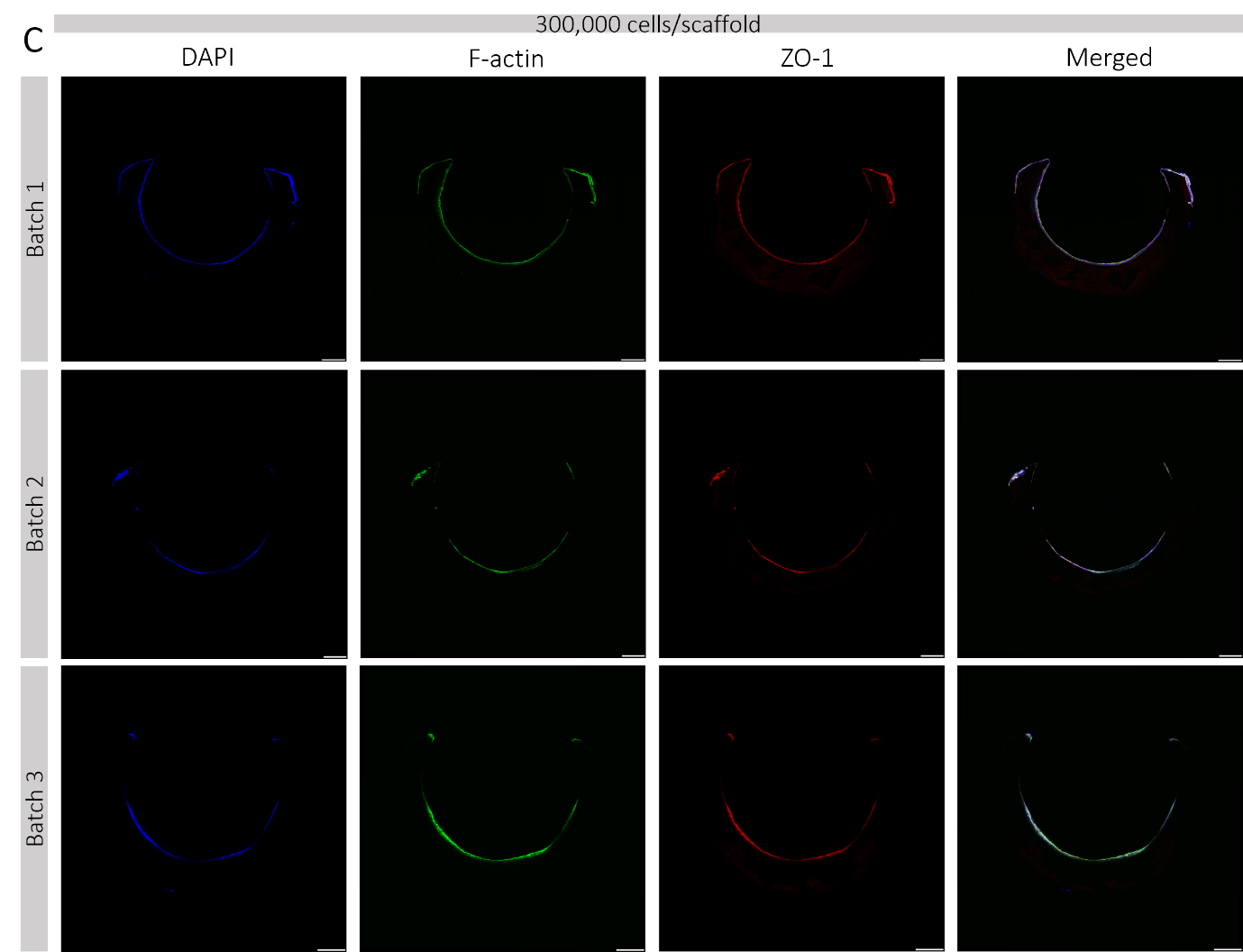
Figure S7.** Evaluation of different Caco-2 cell seeding concentrations after one week; Cross sections. Three different concentrations were tested in cell seeding, without any tilting. Confocal microscopy was used to evaluate cross sections (z-stacks) of (A) 75,000 cells/scaffold, (B) 150,000 cells/scaffold and (C) 300,000 cells/scaffolds. As samples were larger than the microscopic viewing area, multiple images (at 4x magnification) were taken and compiled into one image. 150,000 cells/scaffolds correspond to the same cell number per area as used in the TW system. These cell seeding experiments were conducted in the PDMS holder (with 90 μL of collagen) (SF 6A). Caco-2 cells were stained with (from left to right) DAPI (blue), F-actin (green) and ZO-1 (red). N=3 (three biological replicates, each with one technical replicate). Scaffold = MEW printed half-pipe. Scale bars = 500 μm.

**
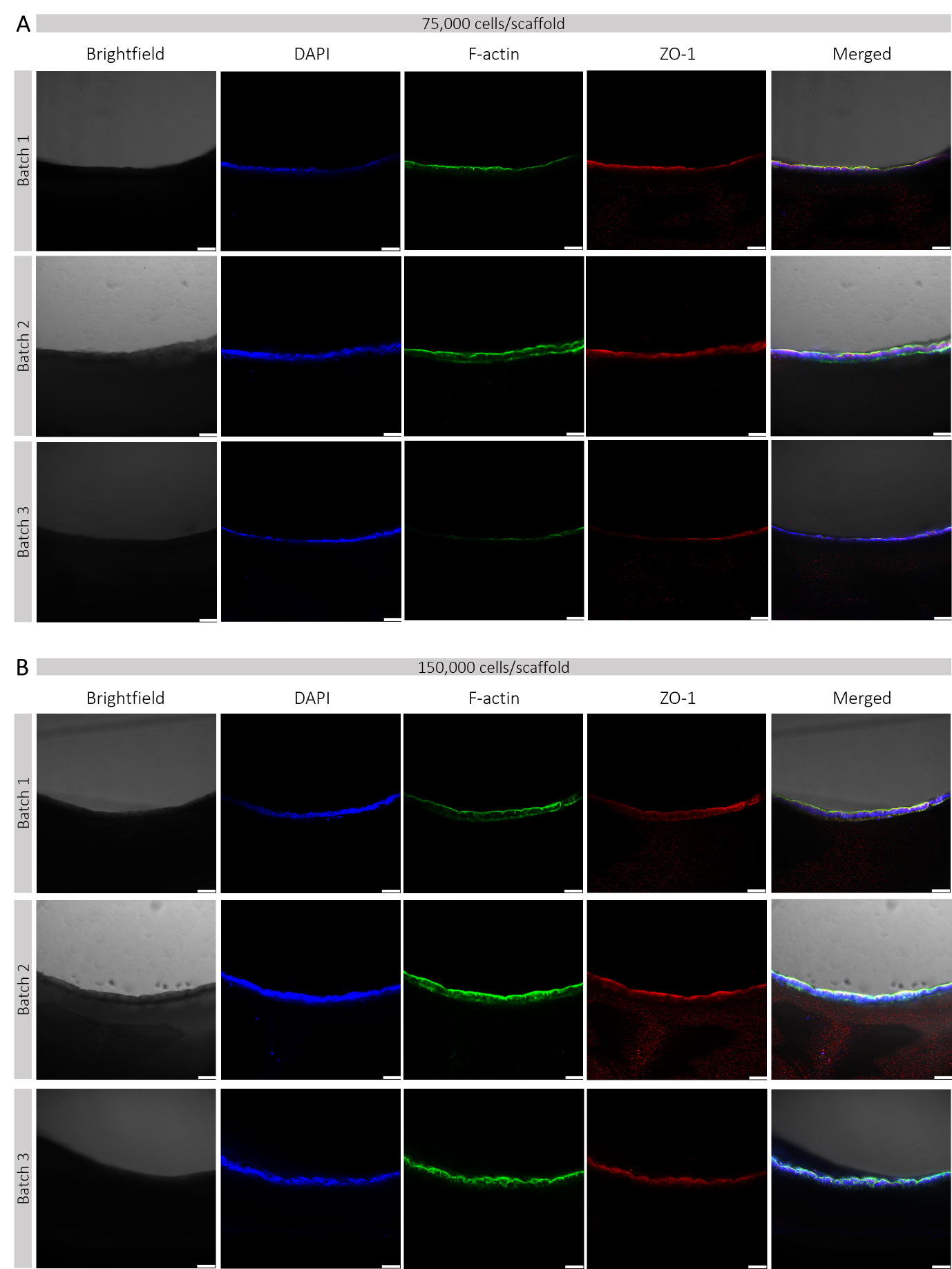

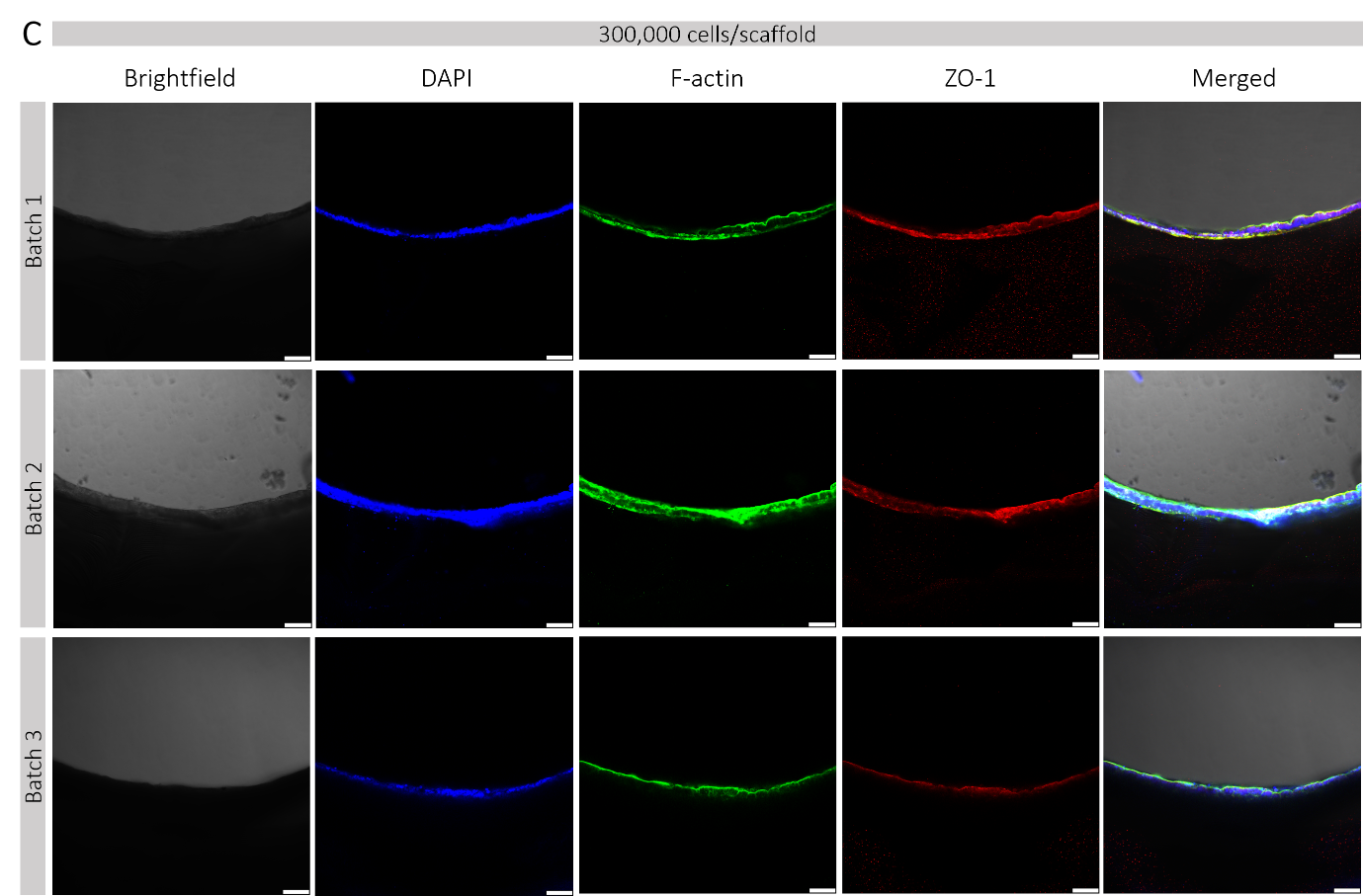
Figure S8.** Evaluation of different Caco-2 cell seeding concentrations after one week; Close-up. Three different concentrations were tested in cell seeding, without any tilting. Confocal microscopy was used to evaluate cross sections (z-stacks, at 10x magnification) of (A) 75,000 cells/scaffold, (B) 150,000 cells/scaffold and (C) 300,000 cells/scaffolds. 150,000 cells/scaffolds correspond to the same cell number per area as used in the TW system. These cell seeding experiments were conducted in the PDMS holder (with 90 μL of collagen) (SF 6A). Caco-2 cells were stained with DAPI (blue), F-actin (green) and ZO-1 (red). N=3 (three biological replicates, each with one technical replicate). Scaffold = MEW printed half-pipe. Scale bars = 100 μm.

Since none of these concentrations resulted in full cell coverage of the entire scaffold within one week. 150,000 and 300,000 cells/scaffold were used for further cell seeding experiments in which tilted cell seeding was optimized. Only a combination of tilting the IoCs at a 60° angle and a concentration of 300,000 cells/scaffold as described in the methods resulted in a full monolayer after one week. Smaller tilting angles of 10-30° and lower cell concentrations (data not shown) did not result in a full monolayer after one week.

**
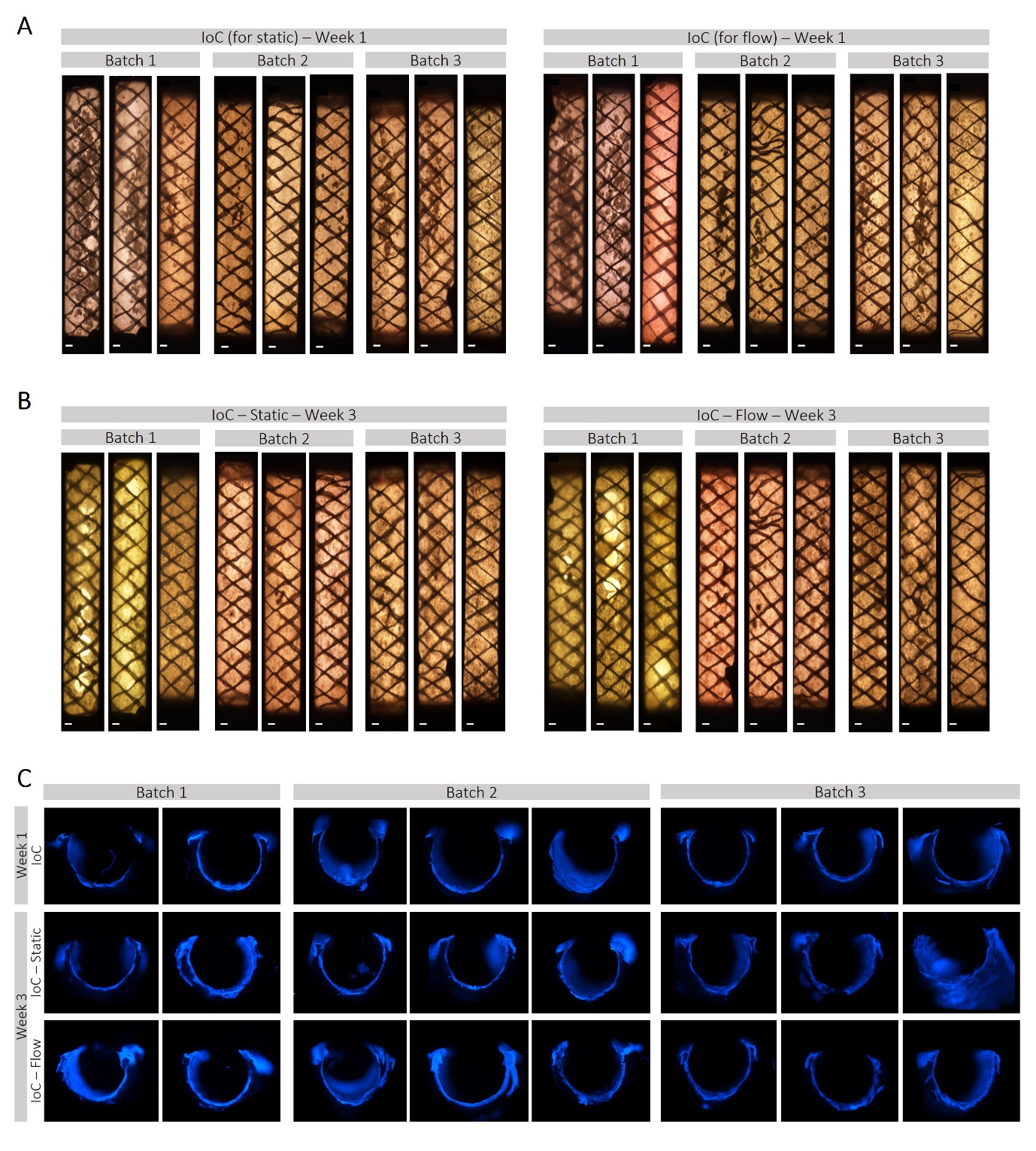
**

**Figure S9.** Evaluation of Caco-2 cell seeding (300,000 cells/IoC) after one and three weeks, with tilting during seeding. Brightfield and fluorescent microscopy images (compiled images at 4x magnification) showing morphology and cell coverage of intestinal epithelial cells on the collagen cast (60-70 µL) MEW half-pipe scaffold in the IoC (flow and static condition) for one and three weeks in comparison to a standard TW model, both biological triplicates (N=3), and each in technical duplicates/triplicates. (A-B) Brightfield overview images of full half-pipe scaffolds in the IoC. The same tubes were imaged at the (A) one-week time point and then at the (B) three-week time point. MEW half-pipes with clear holes in the Caco-2 monolayer after three weeks of culture, were excluded from further assays and analysis. Scale bar = 500 µm (C) Compiled images generated by a fluorescent microscope from a cross section of the IEC-half-pipe (⌀ = 3 mm) after one and three weeks of culture in a static or dynamic (applied flow) conditions. Only cell nuclei were stained by DAPI (blue).

**
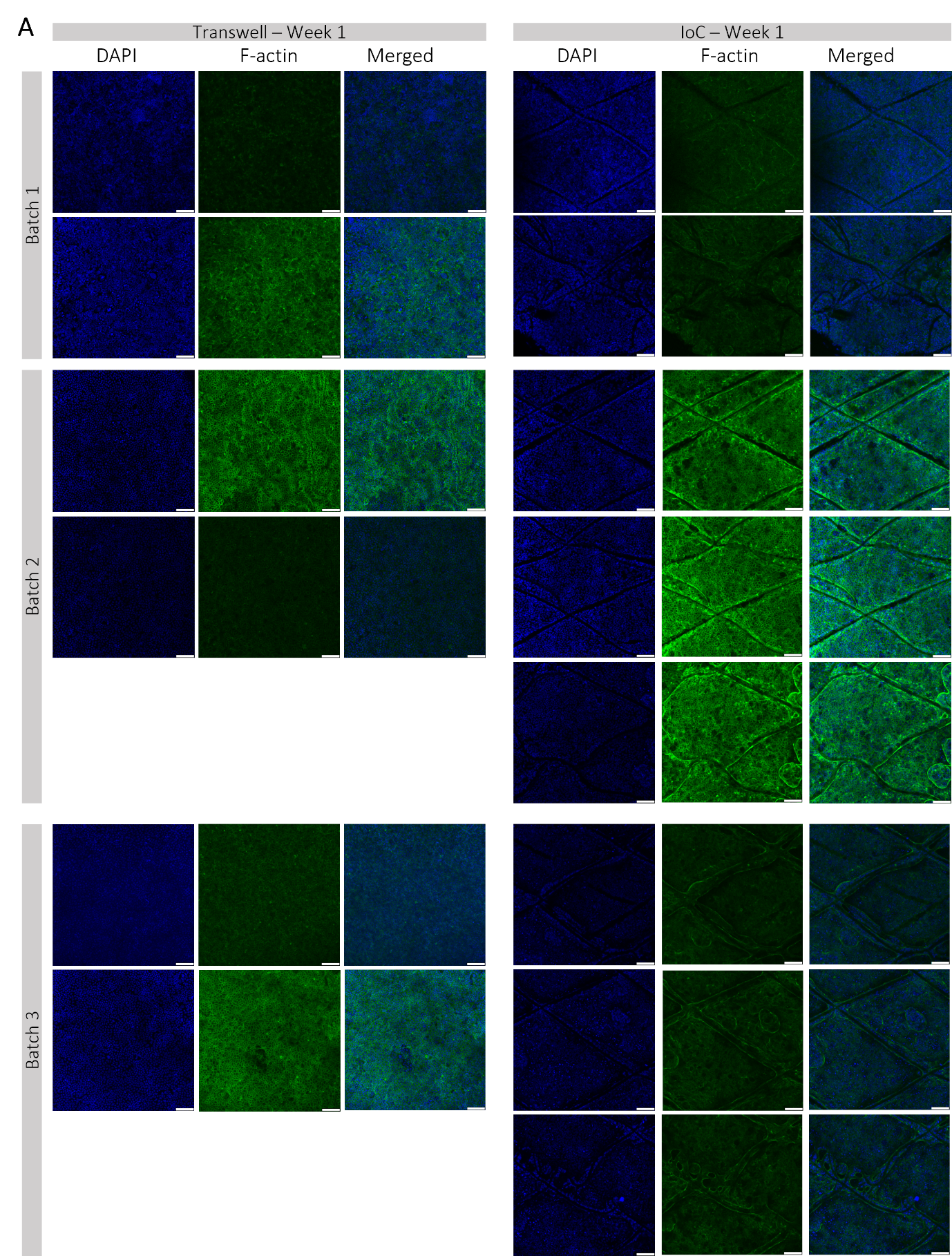

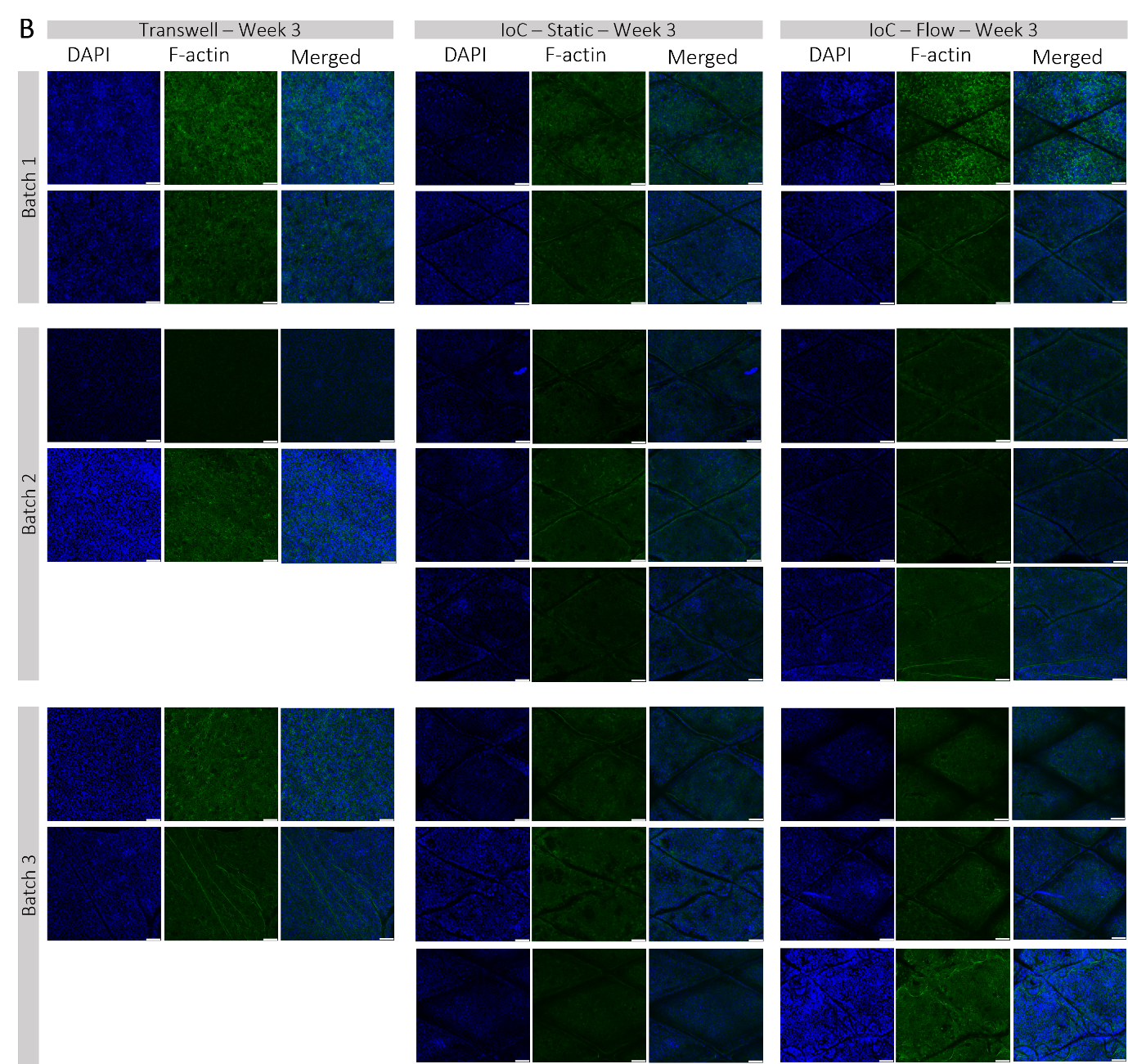
Figure S10.** Evaluation of Caco-2 cell seeding (300,000 cells/IoC) by confocal microscopy (top view), after one and three weeks with tilting during seeding. Top view of confocal images (z-stacks, at 10x magnification) showing morphology and cell coverage of intestinal epithelial cells on the collagen cast (60-70 µL) MEW half-pipe scaffold in the IoC for (A) one week (static) and (B) three weeks (flow and static condition) in comparison to a standard TW model, both in biological triplicates (N=3), and each in technical duplicates/triplicates. These MEW half-pipe batches correspond with the brightfield top view and fluorescent microscopy cross section images of SF9. Caco-2 cells were stained with DAPI (blue) and F-actin (green). Scale bar = 100 μm.

**
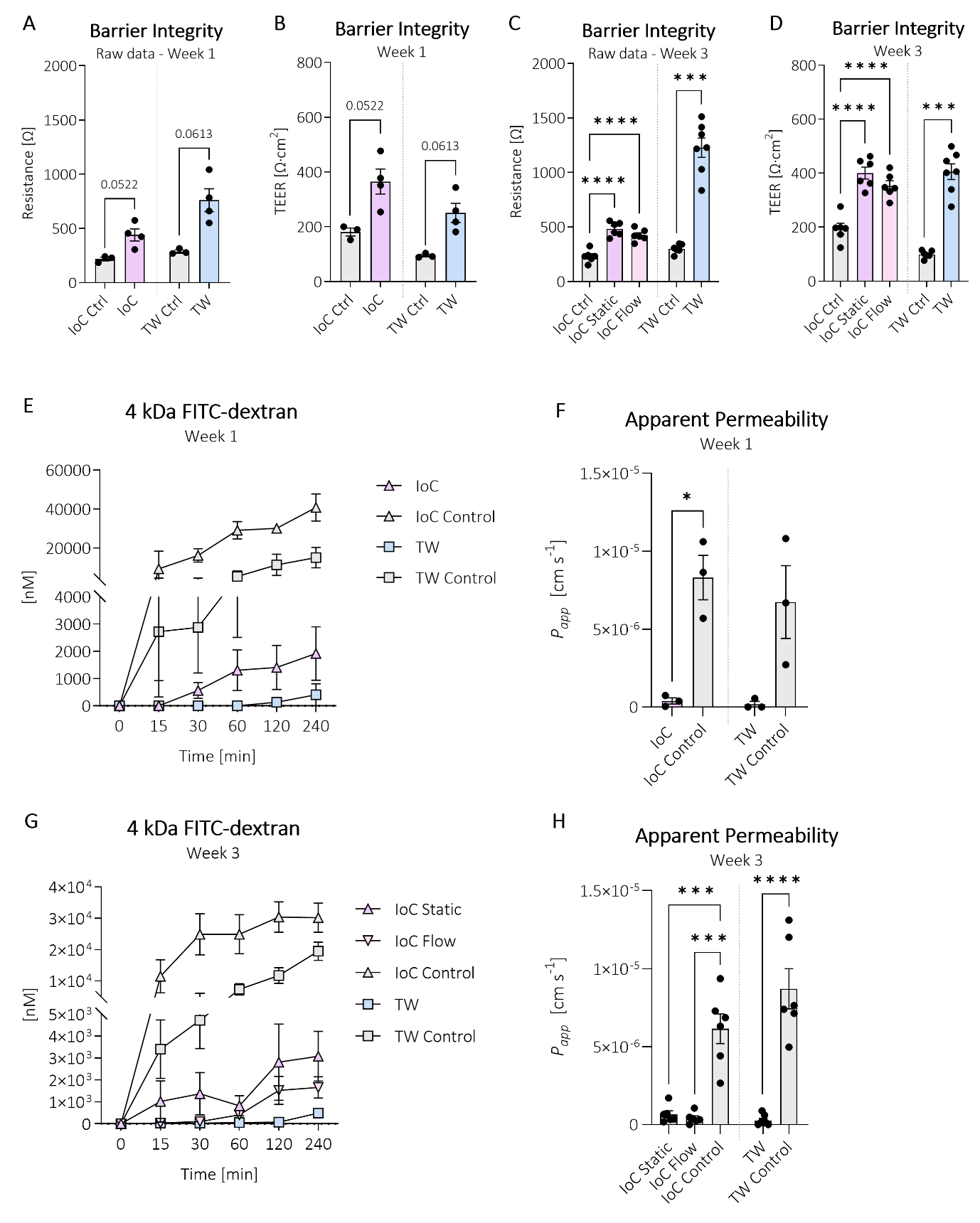
Figure S11.** Functionality of intestinal epithelial cells on the collagen coated MEW half-pipe scaffolds in the IoC in comparison to a standard TW model; unprocessed data and detailed comparisons of the Caco-2 barrier. (A-D) Barrier integrity measured by transepithelial electrical resistance (TEER); (A) Ohm (Ω) measurements and (B) ohm (Ω) measurements corrected for IoC and TW cell growth area (Ω·cm^2^) after one week. (C) Ohm (Ω) measurements and (D) ohm (Ω) measurements corrected for IoC and TW cell growth area (Ω·cm^2^) after three weeks. After one and three weeks of IECs culture in both systems, apparent permeability ($P_{app}$) was determined by the 4 kDa FITC-dextran assay. Unprocessed data of 4 kDa FITC-dextran (nM) over a four hour time period after (E) one week and (G) three weeks (no statistical tests were performed). $P_{app}$ (cm s^-1^) values after (F) one week and after (H) three weeks of IEC culture in IoC or TW, both over a time period of 240 minutes of 4 kDa FITC-dextran exposure. IoC = intestine-on-a-chip, TW = transwell-like system (ThinCert^®^). TW or IoC control means only collagen hydrogel, without cells. Data was statistically tested using paired t-test, Wilcoxon matched-pairs signed-rank test, or mixed-effects ANOVA with Tukey's post hoc was performed. All conditions were tested against the negative control (IoC or TW control) only. Dotted lines represent datasets that were statistically analyzed separately. Datapoints within bars represent at least three different biological replicates, and, if possible, each performed in technical duplicates/triplicates. Error bars represent mean ± SEM. * = p ≤ 0.05; ** = p ≤ 0.01; *** = p ≤ 0.001; **** = p ≤ 0.0001.

**
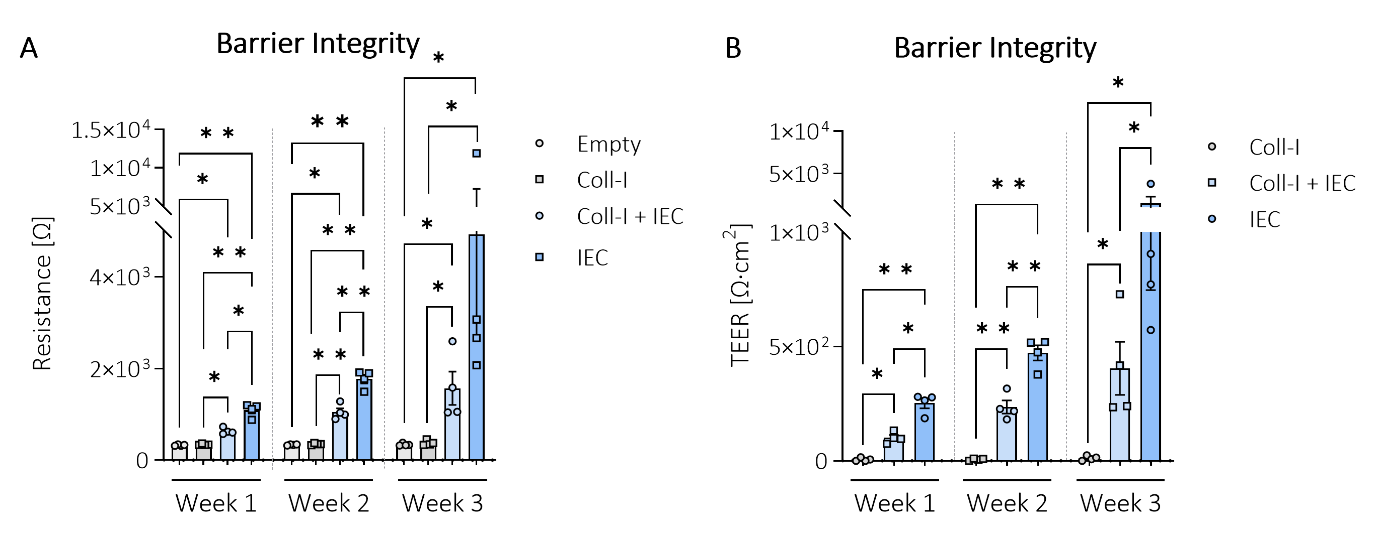
Figure S12.** Transepithelial electrical resistance in TW cultures of Caco-2, seeded with and without a collagen-I hydrogel below the Caco-2 cells. To investigate the effect observed in IoCs and TW with lower transepithelial electrical resistance (TEER) values than expected, an experiment was performed to assess the effect of the collagen-I hydrogel below the Caco-2 cells. TWs were first loaded with a thin collagen-I hydrogel or were left untreated. After polymerization, Caco-2 cells were seeded on top of the hydrogel or directly onto the (pre-wetted) TW filter. TEER was measured after three weeks of culture and shown as (A) unprocessed TEER values (Ω) and (B) TEER corrected for empty TW filter and cell growth area (Ω·cm^2^). Empty = TW filter only, Coll-I = TW insert with a collagen-I hydrogel layer, IEC = intestinal epithelial cell (Caco-2). Error bars represent mean ± SEM, four different biological replicates of Caco-2 cells (N=4), * = p ≤ 0.05; ** = p ≤ 0.01.

**
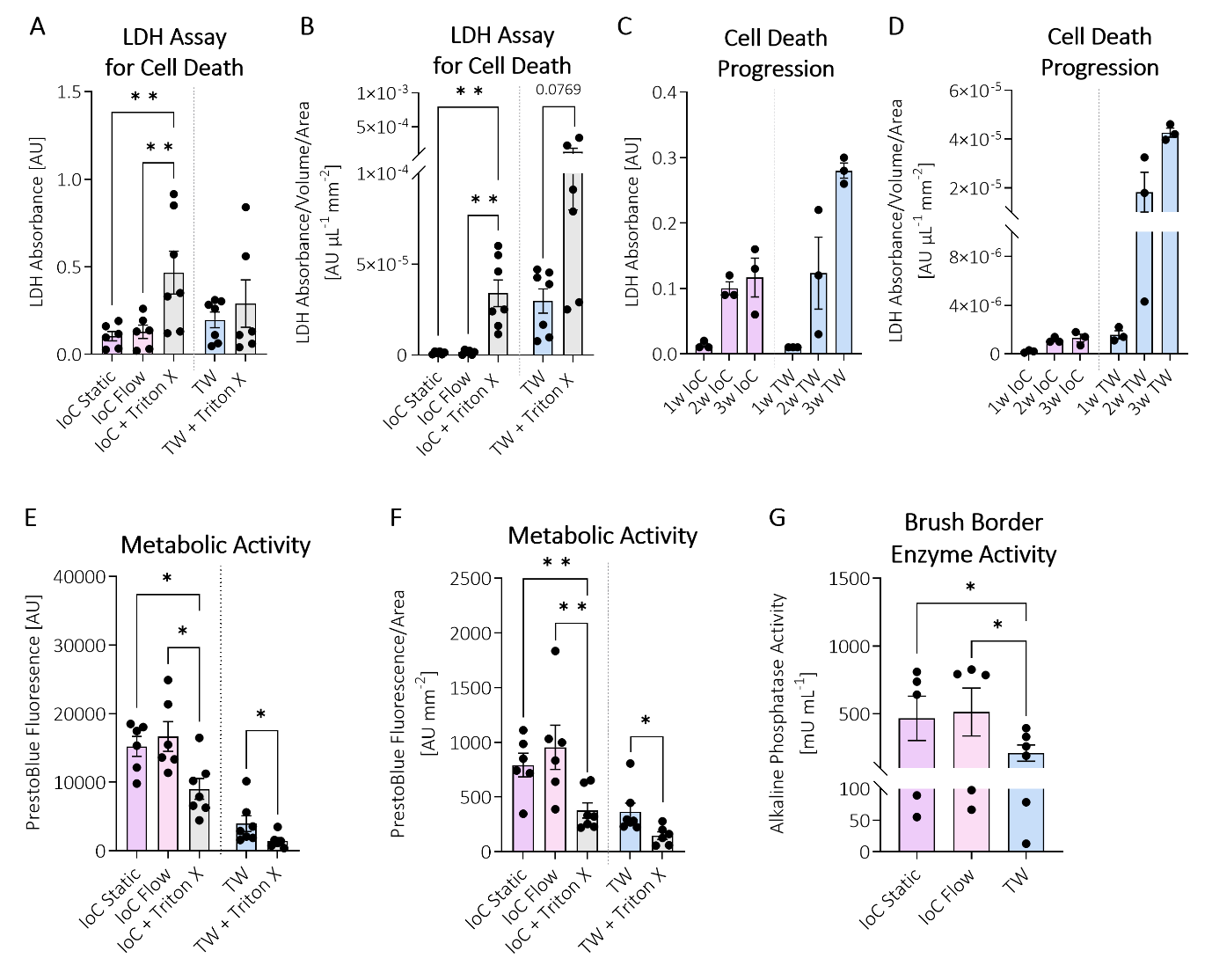
Figure S13.** Intestinal epithelial cells on the collagen coated MEW half-pipe scaffolds in the IoC in comparison to a standard TW model; unprocessed data and comparisons of the Caco-2 functionality compared to controls. After three weeks of IECs culture in the IoCs and TWs, cell functionality was assessed by (A-D) LDH absorbance to measure natural cell death, (E-F) PrestoBlue^TM^ to assess metabolic activity, and (G) alkaline phosphatase enzyme activity assay to measure brush border enzyme activity. LDH assay; (A) Unprocessed LDH absorbance values and (B) LDH absorbance values corrected for volume (µL) and area of the cell growth area (mm^2^). (C) LDH absorbance and (D) absorbance corrected for volume and cell growth area were measured after one week, two weeks and after three weeks, to observe the LDH progression over time (N=1, showing 3 technical replicates). Metabolic activity; (E) Unprocessed PrestoBlue^TM^ fluorescence measurements and (F) PrestoBlue^TM^ fluorescence corrected for cell growth area (mm^2^). Brush border enzyme activity; (G) Unprocessed data of alkaline phosphatase enzyme activity assay measurements. IoC = intestine-on-a-chip, TW = transwell-like system (ThinCert^®^). Data was statistically tested using a paired t-test or mixed-effects ANOVA with Tukey's post hoc. Dotted lines represent datasets that were statistically analyzed separately. IoC and TW conditions were compared to their corresponding control conditions (Triton X, at a 100x dilution), (G) all conditions were compared to each other, or (C+D; N=1, technical replicates) no test was performed. Datapoints within bars represent at least five different biological replicates, and, if possible, each performed in technical duplicates/triplicates. Error bars represent mean ± SEM. * = p ≤ 0.05; ** = p ≤ 0.01.

**
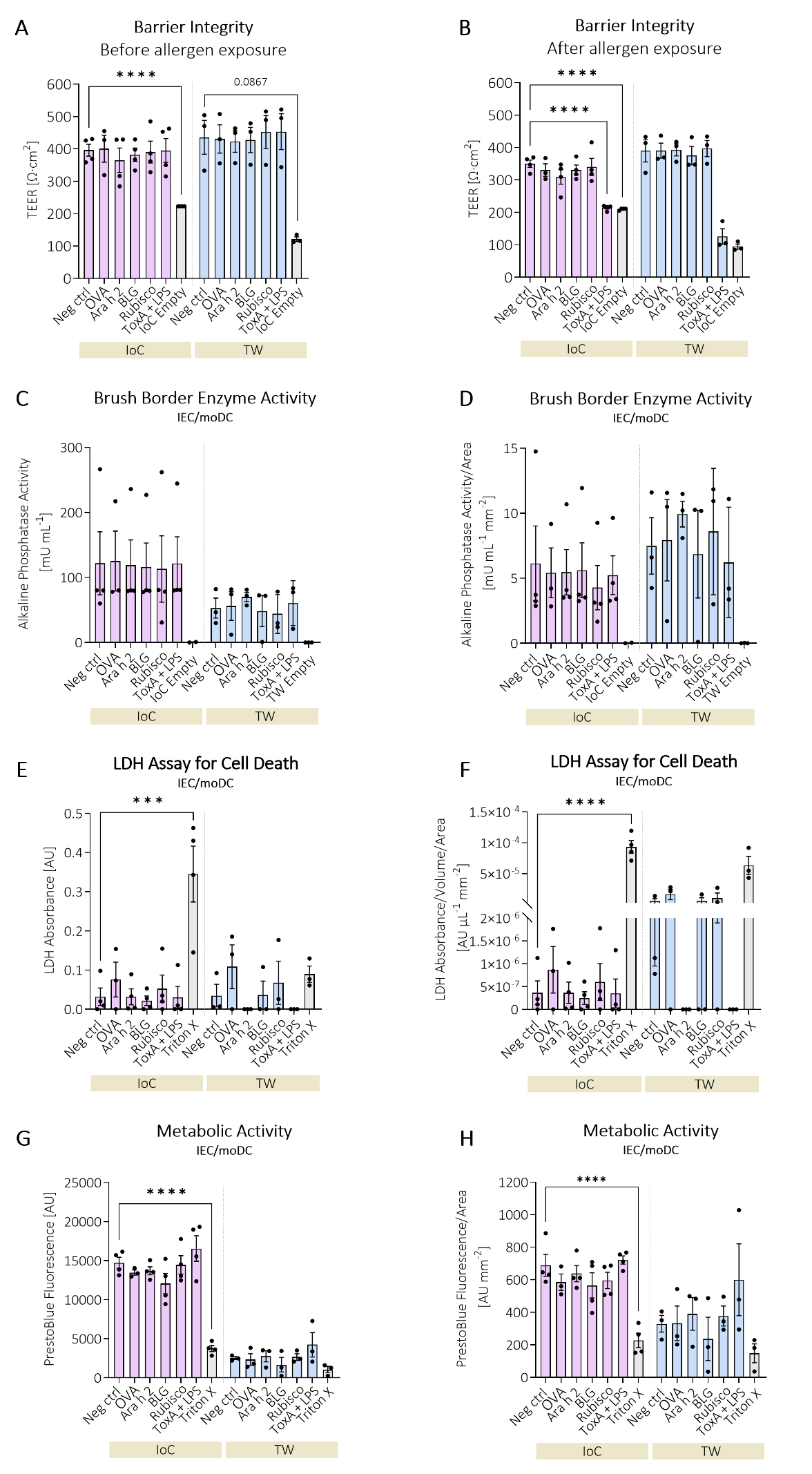
**

**Figure S14.** IEC barrier function of IEC/moDC co-cultures IoCs and TWs). IECs were grown for three weeks in the IoC device or TW system. After three weeks, moDCs were basolateral co-cultured with the IECs for 48 hours, while the IEC were exposed to allergens, non-allergens or toxin A + LPS. After 48 hours, moDCs were retrieved from the hydrogel and the IECs were tested for their barrier integrity measured by transepithelial electrical resistance (TEER); Ohm (Ω) measurements (A) before allergen exposure and (B) after 48 hours of exposure, including IoC and TW empty controls (IoC and TW without cells). Ohm (Ω) measurements were corrected for IoC and TW cell growth area (Ω·cm^2^). Alkaline phosphatase enzyme activity assay: (C) unprocessed values of brush border enzyme activity and (D) brush border enzyme activity corrected for cell growth area. LDH assay; (E) Unprocessed LDH absorbance values and (F) LDH absorbance values corrected for volume (µL) and area of the cell growth area (mm^2^). Metabolic activity; (G) Unprocessed PrestoBlue^TM^ fluorescence measurements and (H) PrestoBlue^TM^ fluorescence corrected for cell growth area (mm^2^). IoC = intestine-on-a-chip, TW= transwell-like system (ThinCert^®^), IEC = intestinal epithelial cell, moDC = monocyte-derived dendritic cell, Neg ctrl = negative control, ToxA = toxin A, LPS = lipopolysaccharide. Allergens: OVA = ovalbumin (allergen from egg), Ara h 2 (allergen from peanut) and BLG = β-lactoglobulin (allergen from milk). Non-allergen Rubisco. Data represents three (N=3) or four (N=4) biologically different immune cell donors, each co-cultured with a different biological replicate of Caco-2 cells. Dotted lines represent datasets that were statistically analyzed separately. Data was statistically tested using repeated measures one-way ANOVA with Dunnett's post hoc, repeated measures one-way ANOVA with Geisser-Greenhouse correction with Dunnett's post hoc, mixed-effects ANOVA with Dunnett's post hoc, or Friedman's test with Dunn's post hoc. All conditions were tested against the negative control. Error bars represent mean ± SEM. *** = p ≤ 0.001; **** = p ≤ 0.0001.

**
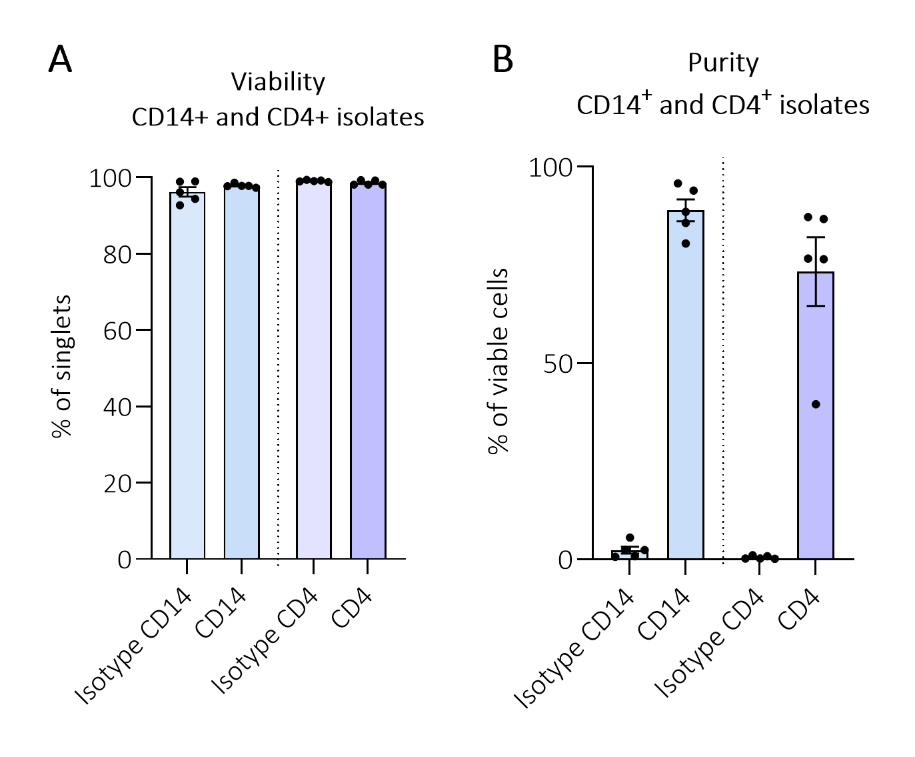
**

**Figure S15.** Purity and viability of CD14^+^ and CD4^+^ MACS sorting isolates. CD14^+^ monocytes and CD4^+^ naive T cells were isolated from freshly isolated PBMCs from 5 independent donors (N=5). After isolation, the CD14^+^ and CD4^+^ isolates we tested on (A) viability and (B) purity by flow cytometry. Viability was stained for using Fixable Viability Dye eFluor™ 780 (APC-Cy7; 65-0865-14). CD14^+^ monocytes were stained using CD14 Monoclonal Antibody (61D3), PerCP-Cyanine5.5 (45-0149-42) or Mouse IgG1 kappa Isotype Control (P3.6.2.8.1), PerCP-Cyanine5.5 (45-4714-82). CD4^+^ naive T cells were stained using CD4 Monoclonal Antibody (OKT4 (OKT-4)), PerCP-Cyanine5.5, eBioscience™ (45-0048) or Mouse IgG2b kappa Isotype Control (eBMG2b), PerCP-Cyanine5.5, eBioscience™ (45-4732-82). Error bars represent mean ± SEM. Both datasets were not statistically tested.

**
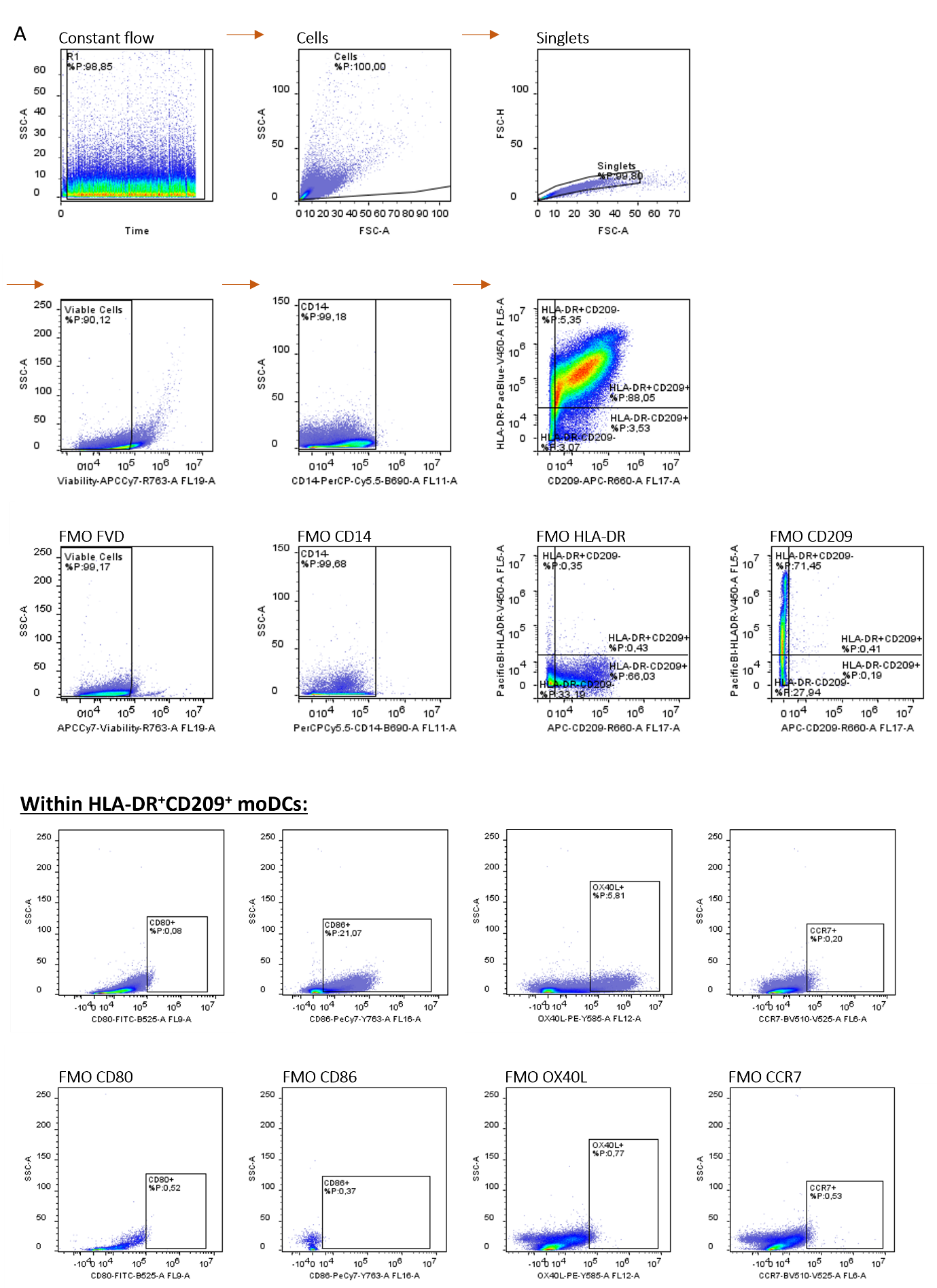

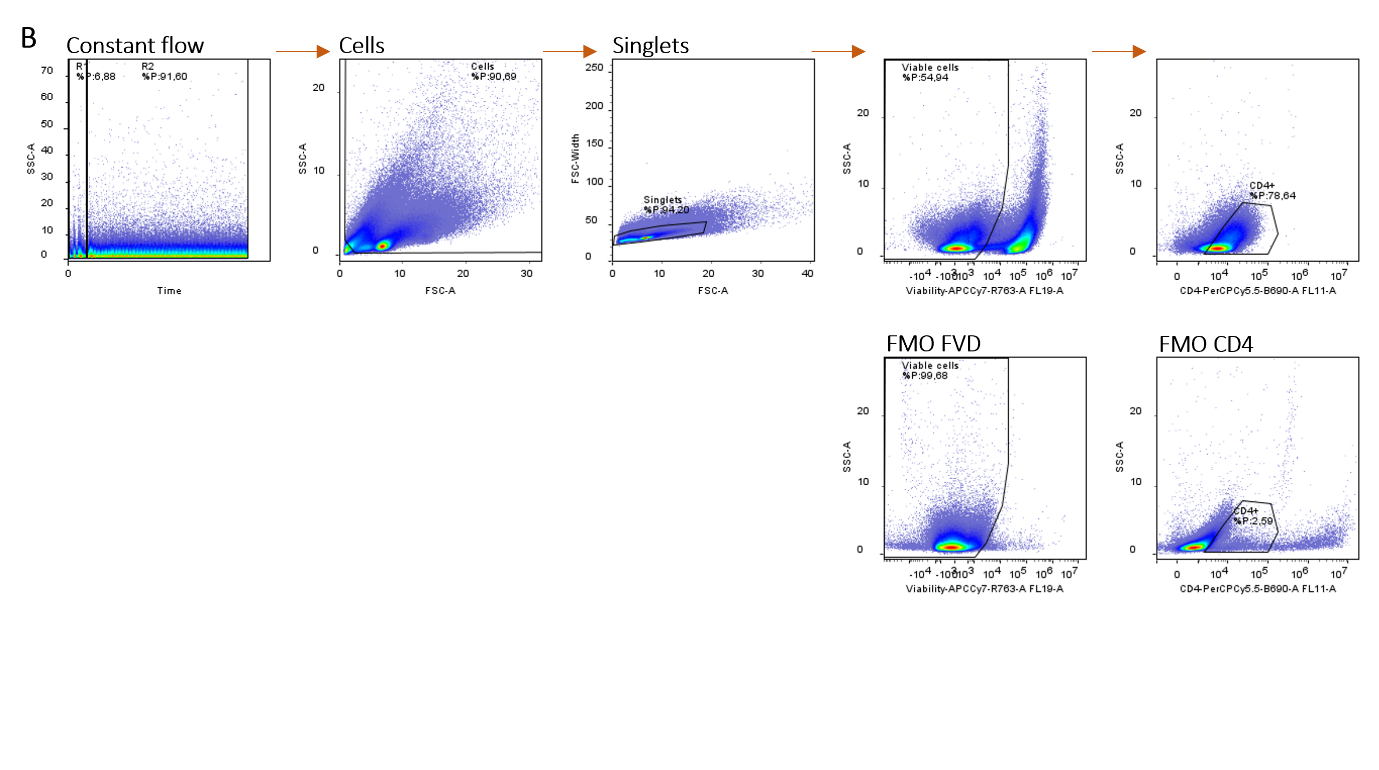

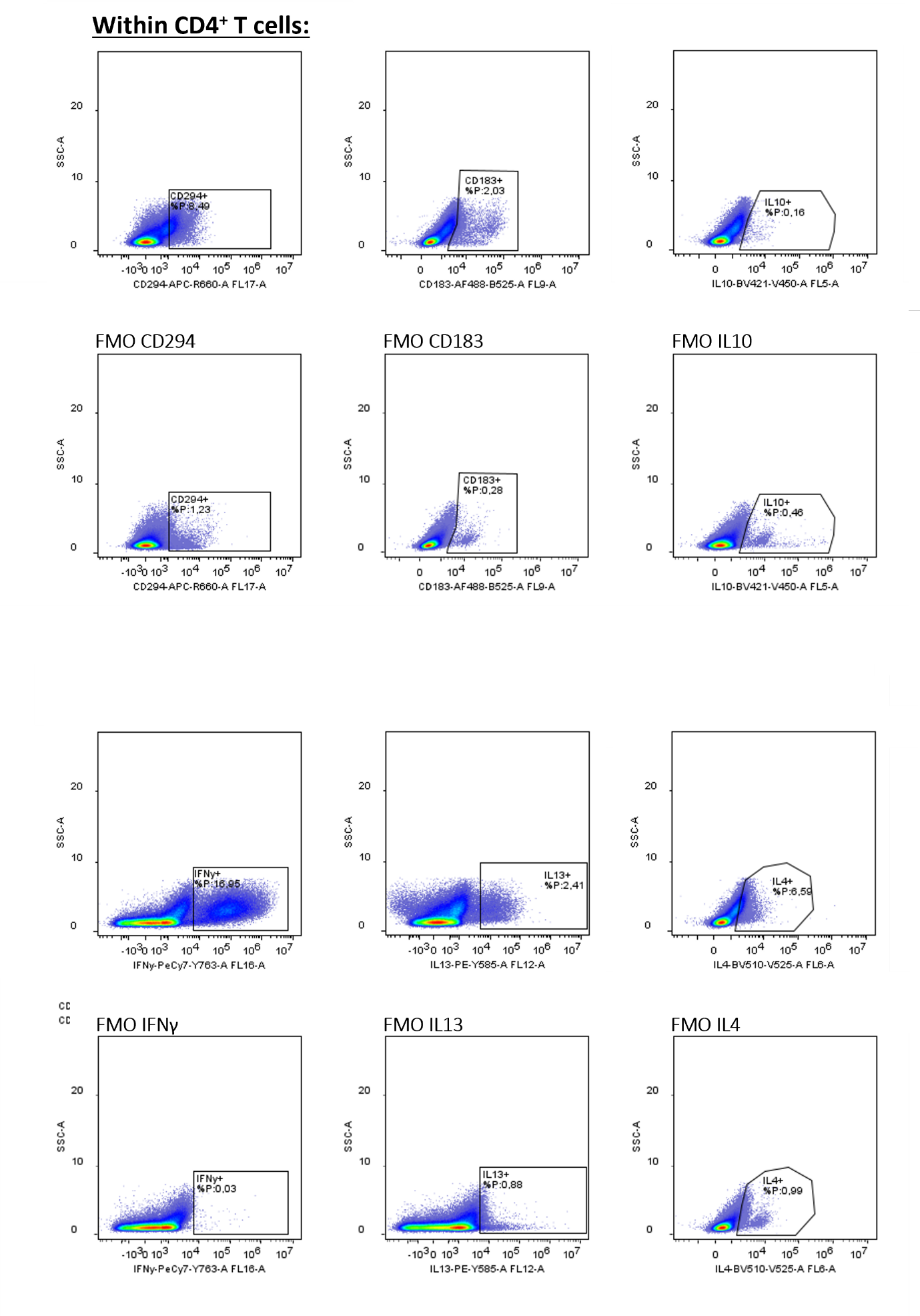
Figure S16.** Gating strategy for flow cytometry analysis for moDCs and CD4^+^ T cells. (A) MoDC gating strategy and corresponding FMOs (fluorescence minus one) including the corresponding isotypes. (B) CD4^+^ T cell gating strategy and corresponding FMOs (fluorescence minus one) including the corresponding isotype.

**
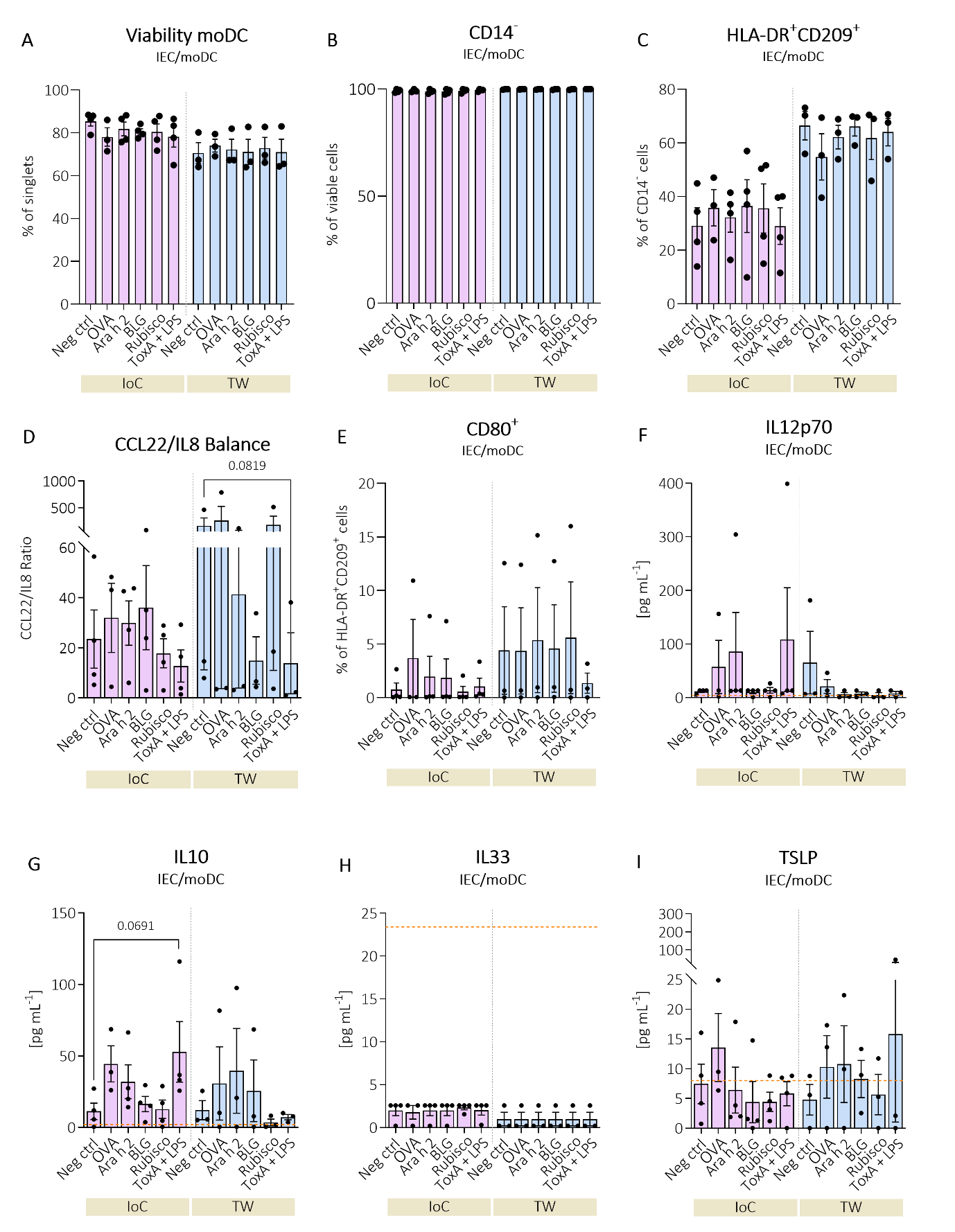
Figure S17.** Phenotype and cytokine secretion of moDCs co-cultured with IECs (from moDC/IEC co-cultures in IoC/TW). IECs were grown for three weeks in the IoC device or TW system. After three weeks, moDCs were basolaterally co-cultured with the IECs for 48 hours, while the IEC were exposed to allergens, non-allergens or toxin A + LPS. After 48 hours, moDCs were retrieved from the hydrogel and (A) viability, (B) CD14^-^ and (C) HLA-DR^+^CD209^+^ cells were measured by flow cytometry. A piece of the IEC loaden MEW half-pipe was also taken along into hydrogel digestion of the other moDCs, to take along possible infiltrated moDCs into the MEW half-pipes. Therefore, moDC HLA-DR^+^CD209^+^ expression was measured in lower ranges as both Caco-2 as well as moDCs were still present. (D) CCL22/IL8 balance. (E) Co-stimulatory marker CD80 was measured by flow cytometry. Supernatant from IEC/moDC co-cultures was measured by ELISA for (F) IL12p70, (G) IL10, (H) IL33, and (I) TSLP secretion. IoC = intestine-on-a-chip, TW = transwell-like system (ThinCert^®^), IEC = intestinal epithelial cell, moDC = monocyte-derived dendritic cell, Neg ctrl = negative control, ToxA = toxin A, LPS = lipopolysaccharide. Allergens: OVA= ovalbumin (allergen from egg), Ara h 2 (allergen from peanut) and BLG = β-lactoglobulin (allergen from milk). Non-allergen Rubisco. Grey dotted lines represent datasets that were statistically analyzed separately. Orange dotted lines represent detection lower limits of ELISA assay kits. Repeated measures one-way ANOVA with Dunnett's post hoc, mixed-effects ANOVA with Dunnett's post hoc, or Friedman's test with Dunn's post hoc was performed. All conditions were tested against the negative control only. Data represents three (N=3) or four (N=4) independent immune cell donors, each co-cultured with a different biological replicate of Caco-2 cells. Error bars represent mean ± SEM.

**
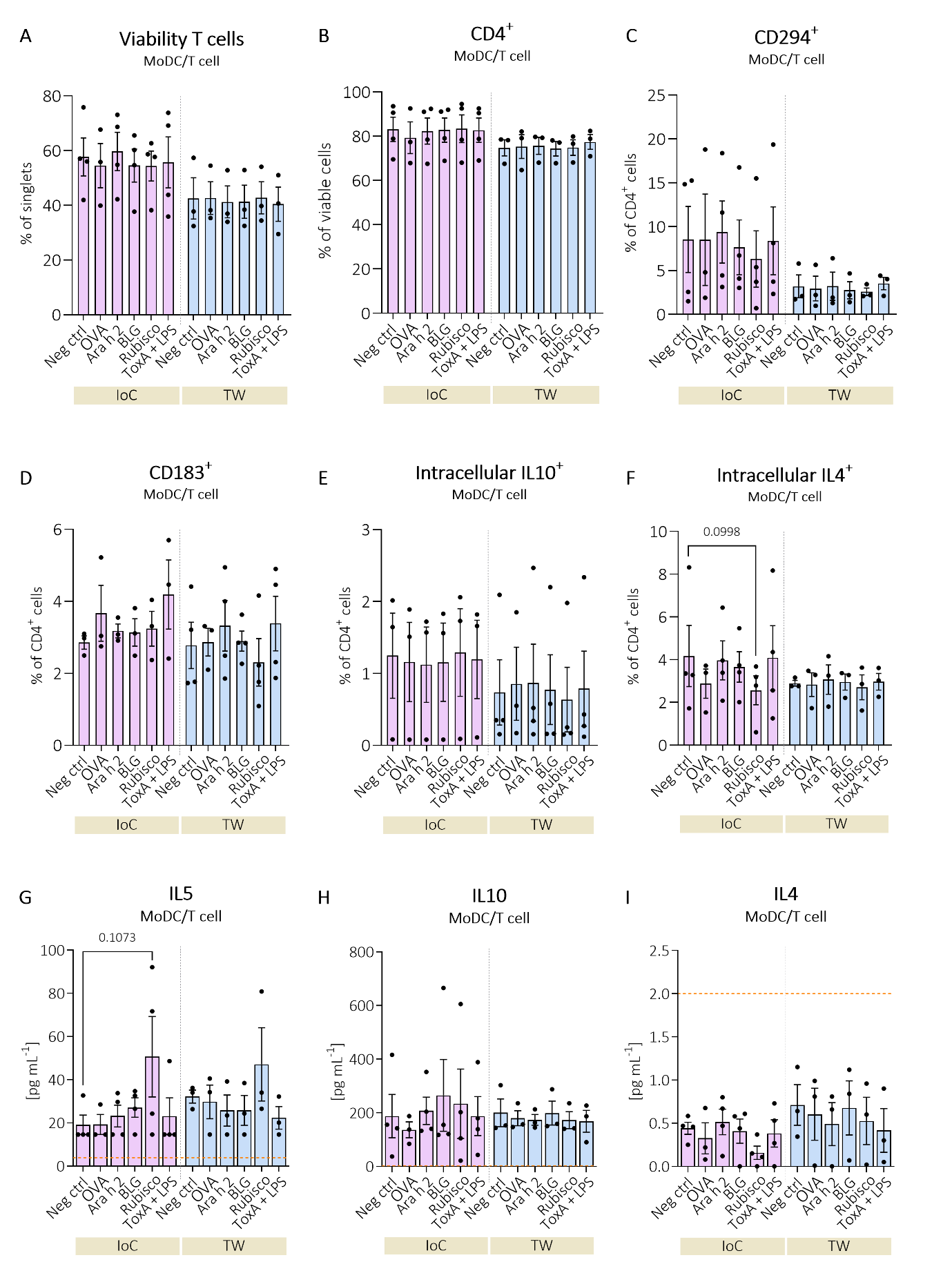
Figure S18.** Phenotype and cytokine secretion of T cells co-cultured with moDCs (from moDC/IEC co-cultures in IoC/TW). IECs were grown for three weeks in the IoC device or TW system. After three weeks, moDCs were basolateral co-cultured with the IECs for 48 hours, while the IEC were exposed to allergens, non-allergens or toxin A + LPS. After 48 hours, moDCs were retrieved from the hydrogels and co-cultured with naive CD4^+^ T cells for 5 days in hydrogel. After 5 days, T cells were retrieved from the hydrogels, restimulated and (A) viability, (B) CD4, (C) CD294, (D) CD183 and intracellular (E) IL10, and (F) IL4 were measured by flow cytometry. Supernatant from non-restimulated T cells was measured by ELISA for (G) IL5, (H) IL10, and (I) IL4 secretion. IoC= intestine-on-a-chip, TW = transwell-like system (ThinCert^®^), IEC = intestinal epithelial cell, moDC= monocyte-derived dendritic cell, Neg ctrl = negative control, ToxA = toxin A, LPS = lipopolysaccharide. Allergens: OVA = ovalbumin (allergen from egg), Ara h 2 (allergen from peanut) and BLG = β-lactoglobulin (allergen from milk). Non-allergen Rubisco. Grey dotted lines represent datasets that were statistically analyzed separately. Orange dotted lines represent detection lower limits of ELISA assay kits. Repeated measures one-way ANOVA with Dunnett's post hoc, mixed-effects ANOVA with Dunnett's post hoc, or Friedman's test with Dunn's post hoc was performed. All conditions were tested against the negative control only. Data represents three (N=3) or four (N=4) independent immune cell donors. Error bars represent mean ± SEM.

**References**

[1] J. Slaats, E. Wagena, D. Smits, et al. “Adenosine A2a Receptor Antagonism Restores Additive Cytotoxicity by Cytotoxic T Cells in Metabolically Perturbed Tumors.” Cancer Immunol Res Name 10, no. 12 (2022): 1462. 10.1158/2326-6066.CIR-22-0113; B. Weigelin, P. Friedl. “A three-dimensional organotypic assay to measure target cell killing by cytotoxic T lymphocytes.” Biochem Pharmacol Name 80, no. 12 (2010): 2087. 10.1016/j.bcp.2010.09.004

[2] S. J. Koppelman, G. A. de Jong, M. Laaper-Ertmann, et al. “Purification and immunoglobulin E-binding properties of peanut allergen Ara h 6: evidence for cross-reactivity with Ara h 2.” Clin Exp Allergy Name 35, no. 4 (2005): 490. 10.1111/j.1365-2222.2005.02204.x

[3] Y. Deng, C. Govers, S. Bastiaan-Net, et al. “Hydrophobicity and aggregation, but not glycation, are key determinants for uptake of thermally processed beta-lactoglobulin by THP-1 macrophages.” Food Res Int Name 120, no. (2019): 102. 10.1016/j.foodres.2019.01.038

[4] A. H. Martin, O. Castellani, G. A. de Jong, L. Bovetto, C. Schmitt. “Comparison of the functional properties of RuBisCO protein isolate extracted from sugar beet leaves with commercial whey protein and soy protein isolates.” J Sci Food Agric Name 99, no. 4 (2019): 1568. 10.1002/jsfa.9335
